# Autoimmunity: Molecular Mechanisms, Biomarkers, and Therapeutic Opportunities

**DOI:** 10.1002/mco2.70905

**Published:** 2026-09-03

**Authors:** Jialong Kuang, Yin Zhu, Fang Xu, Yongjing Liu, Yuan Li, Ling Yuan, Liqin Wei, Bo Huang, Yan Chen, Pei Huang, Zuochen Du

**Affiliations:** ^1^ Department of Pediatrics Affiliated Hospital of Zunyi Medical University Zunyi China; ^2^ Guizhou Children's Hospital Zunyi China; ^3^ Zunyi Medical and Pharmaceutical College Zunyi China; ^4^ Guizhou Biomanufacturing Laboratory Affiliated Hospital of Zunyi Medical University Zunyi China

**Keywords:** autoimmune diseases, biomarkers and precision therapy, epigenetics, functional reprogramming, immunometabolism, posttranscriptional regulation

## Abstract

Autoimmune diseases (ADs) arise from the breakdown of immune tolerance, yet their clinical course, organ involvement, and treatment responses vary markedly across systemic and organ‐specific autoimmune disorders. This diversity cannot be explained by a single pathogenic mechanism or by conventional serological categories alone. Here, we synthesize evidence that autoimmunity is shaped by interacting molecular and cellular regulatory layers, including immunometabolic rewiring, epigenetic remodeling, posttranscriptional regulation, and functional reprogramming of immune and tissue‐resident cells. These processes influence pathogenic T‐cell and B‐cell differentiation, myeloid activation, stromal and epithelial dysfunction, tissue‐specific inflammation, and intercellular communication. We further discuss how multidimensional readouts, including metabolites, methylation and chromatin signatures, RNA‐based markers, and reprogrammed immune‐cell phenotypes, may support diagnosis, disease‐activity assessment, prognosis, and therapeutic stratification. From a therapeutic perspective, this framework supports a shift from broad immunosuppression toward mechanism‐matched interventions targeting metabolic vulnerabilities, epigenetic regulators, RNA–control pathways, and pathogenic cellular states, including emerging cell‐based approaches. We propose that ADs can be viewed as dynamic network disorders in which molecular mechanisms, biomarkers, and therapeutic opportunities are closely connected. Integrating longitudinal multiomics, single‐cell and spatial profiling, and standardized biomarker validation may help translate autoimmune heterogeneity into actionable precision‐medicine strategies for more individualized care.

## Graphical abstract

Major types of immunometabolism, epigenetics, posttranscriptional regulation, and functional reprogramming involved in autoimmune diseases. The roles of immunometabolism, epigenetics, posttranscriptional regulation, and functional reprogramming in autoimmune diseases have been a major research focus in recent years. Understanding the classifications within these four domains is essential for comprehending their contributions to shaping disease heterogeneity. OXPHOS, oxidative phosphorylation; PPP, pentose phosphate pathway; miRNAs, microRNAs; lncRNAs, long noncoding RNAs; circRNAs, circular RNAs; m^6^A, N^6^‐methyladenosine; RNA, ribonucleic acid; DNA, deoxyribonucleic acid; T cell, T lymphocyte; B cell, B lymphocyte. Created in BioRender. L, K. (2026) https://BioRender.com/u1iksg0

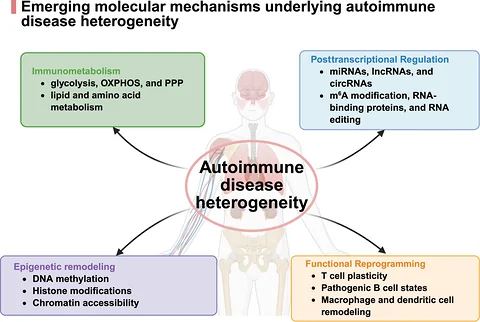

## 1 Introduction

Autoimmune diseases (ADs) develop when immune tolerance to self‐antigens fails, and this loss of tolerance can sustain inflammation, damage tissues, and impair organ function [1]. This disease category includes both systemic and organ‐specific disorders, with systemic lupus erythematosus (SLE), rheumatoid arthritis (RA), Type 1 diabetes (T1D), and multiple sclerosis (MS) representing commonly discussed examples. Their heterogeneity is evident at several levels, including the organs involved, the immune pathways that dominate, the course of disease, and the degree of treatment response. From an epidemiological perspective, ADs are also becoming a more prominent public health concern. In an analysis based on the Global Burden of Disease 2019 dataset, the four ADs examined did not follow the same incidence trend: the global age‐standardized incidence rate increased for RA, but declined for inflammatory bowel disease, MS, and psoriasis between 1990 and 2019 [2]. Even so, the projected number of incident cases is expected to rise for all four diseases through 2040 [2].

At the pathogenic level, ADs are characterized by inappropriate recognition of self‐antigens and by failure of tolerance mechanisms that normally keep autoreactive lymphocytes under control [1]. In healthy immune regulation, central tolerance in the thymus and bone marrow is complemented by peripheral mechanisms that restrain escaped autoreactive lymphocytes, including regulatory T‐cell (Treg)‐mediated suppression, tolerogenic antigen‐presenting cell (APC)‐mediated restraint, T‐cell quiescence, ignorance, anergy, exhaustion, senescence, and cell death/deletion, as well as B‐cell anergy, follicular exclusion, reduced survival, clonal ignorance, and germinal‐center tolerance [3, 4, 5]. When genetic susceptibility, environmental exposure, and immune dysregulation converge, these safeguards may become insufficient [1]. Autoreactive T and B cells can then persist or expand, with downstream inflammatory cascades, autoantibody production, and tissue injury [1]. The coexistence of different ADs within the same patient also argues against viewing each autoimmune phenotype as entirely distinct; at least some of them may share pathogenic mechanisms or predisposing factors [6].

Despite advances in glucocorticoids, conventional immunosuppressants, biologic agents, and emerging cell‐based therapies, current AD management still relies on immunosuppression and symptom control [1]. Consequently, many patients still do not achieve complete disease control, and treatment‐related adverse effects remain a major concern [1]. Diagnosis and monitoring also often rely on autoantibody profiles, inflammatory indices, and assessment of organ damage [1]. Reliable biomarkers for early detection, patient stratification, and prediction of treatment response are still limited [7]. The induction of remission reported after cluster of differentiation 19 (CD19)‐targeted chimeric antigen receptor T‐cell (CAR‐T) therapy in refractory SLE provides a concrete example of how mechanism‐based intervention may change treatment possibilities [8]. However, it remains unclear whether this approach will prove safe over the long term or broadly applicable across different AD subsets [1, 8]. These limitations make it necessary to look beyond conventional clinical and serological readouts and to define biomarkers and therapeutic targets within deeper molecular regulatory networks.

In this context, autoimmune heterogeneity can be examined through four interrelated regulatory dimensions: immunometabolic rewiring, epigenetic remodeling, posttranscriptional fine‐tuning, and immune‐cell functional reprogramming. These layers are unlikely to operate as isolated processes. Rather, they intersect in the way immune cells sense environmental cues, maintain pathogenic or regulatory states, handle disease‐relevant transcripts, and develop distinct functional phenotypes. Metabolic rewiring affects T‐cell proliferation, differentiation, and effector programs [9, 10]. Epigenetic remodeling can stabilize transcriptional states over time and thereby contribute to the persistence of autoimmune inflammation, while epigenetic signatures may support patient stratification [11]. Posttranscriptional regulation further shapes transcript fate and inflammatory output [12, 13]. Functional reprogramming, as exemplified in Treg cells, can reflect the phenotypic consequences of upstream perturbations within specific cellular and tissue contexts [14]. Together, these dimensions provide a conceptual framework for understanding how shared immune dysregulation can generate diverse disease phenotypes, biomarker patterns, and therapeutic vulnerabilities.

Many recent reviews have examined autoimmune heterogeneity from the perspective of individual diseases, specific immune‐cell subsets, or single regulatory layers, such as metabolism, epigenetics, noncoding ribonucleic acids (ncRNAs), or cellular immune dysfunction [14, 15, 16, 17, 18, 19, 20]. Although these approaches have provided important mechanistic insights, they may not fully explain how distinct molecular abnormalities converge to generate disease‐ and tissue‐specific phenotypes, or how such abnormalities can be translated into biomarker discovery and therapeutic stratification. The distinctive value of this review is therefore not to summarize these regulatory layers separately, but to organize them into a mechanism‐to‐biomarker‐to‐therapy framework. By comparing immunometabolic, epigenetic, posttranscriptional, and functional reprogramming mechanisms across autoimmune settings, we highlight how shared immune dysregulation can produce divergent clinical manifestations and how multidimensional molecular readouts may support more precise patient classification and intervention.

In this review, we aim to address three key questions: how immunometabolic, epigenetic, posttranscriptional, and functional reprogramming networks jointly shape the heterogeneity of ADs; how these multidimensional regulatory abnormalities can be translated into clinically informative biomarkers; and which regulatory circuits should be prioritized as targets for mechanism‐based, precision‐oriented therapeutic intervention. Accordingly, Sections 2, 3, 4, 5 review the major mechanistic dimensions underlying heterogeneity in ADs, Section 6 synthesizes their biomarker implications, and Section 7 discusses their therapeutic relevance. By synthesizing current evidence within this framework, we aim to provide a more integrated perspective on the molecular basis of heterogeneity in ADs and its translational implications.

## 2 Role of Immunometabolism in Shaping Heterogeneity in ADs

**FIGURE 1:**
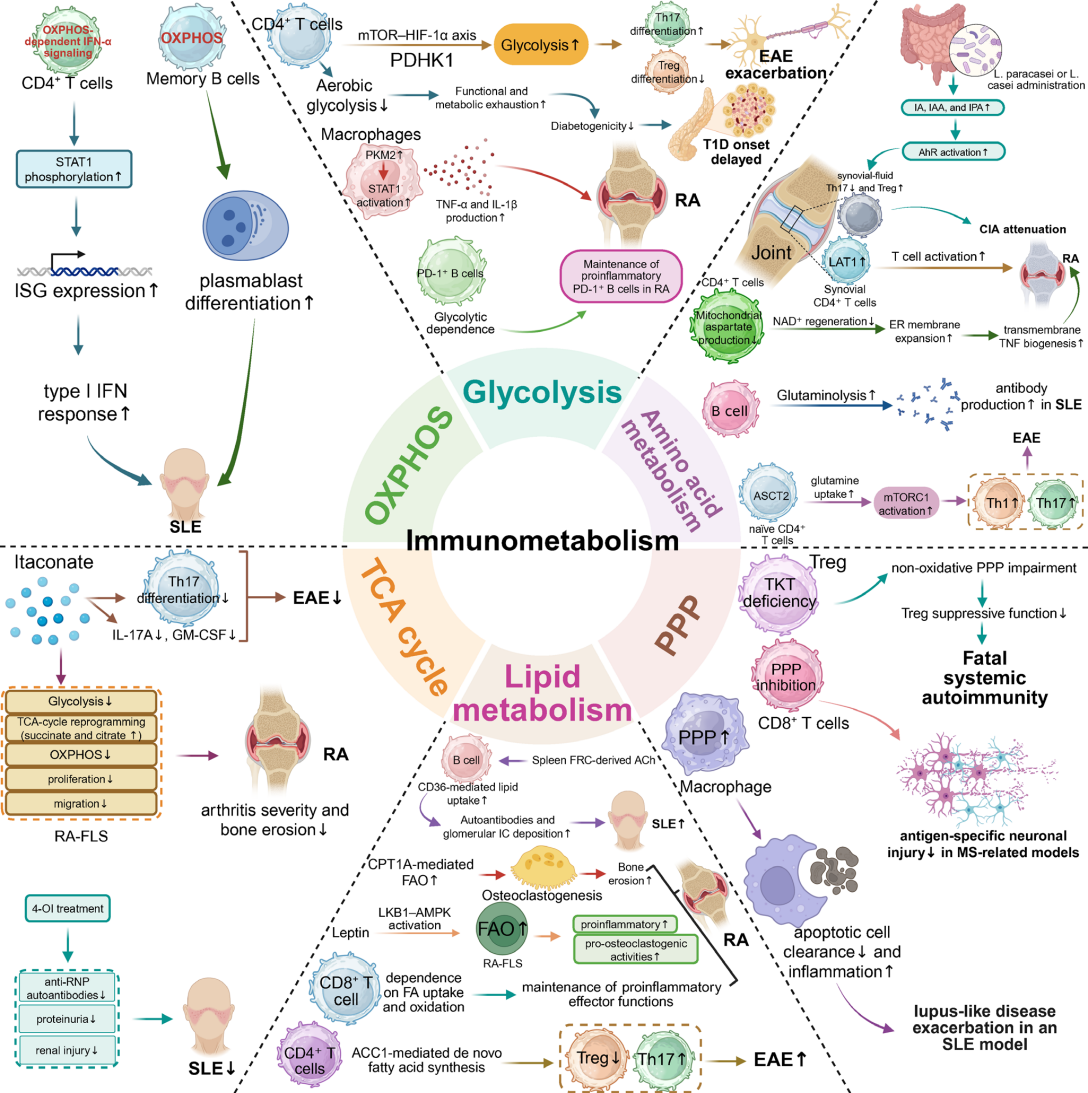
Immunometabolic programs shaping autoimmune heterogeneity. The figure summarizes how glycolysis, OXPHOS, lipid metabolism, amino acid metabolism, TCA‐cycle metabolites, and the PPP regulate immune‐cell fate, stromal activation, inflammatory amplification, and tissue injury across representative autoimmune diseases. Disease‐ and cell‐specific metabolic dependencies may provide both biomarkers and therapeutic vulnerabilities. ACC1, acetyl‐CoA carboxylase 1; AhR, aryl hydrocarbon receptor; ASCT2, alanine–serine–cysteine transporter 2; CD36, cluster of differentiation 36; CPT1A, carnitine palmitoyltransferase 1A; EAE, experimental autoimmune encephalomyelitis; FAO, fatty acid oxidation; RA‐FLS, rheumatoid arthritis fibroblast‐like synoviocyte; GM‐CSF, granulocyte‐macrophage colony‐stimulating factor; HIF‐1α, hypoxia‐inducible factor 1α; IFN, interferon; IFN‐α, interferon‐α; IL‐1β, interleukin‐1β; IL‐17A, interleukin‐17A; LAT1, L‐type amino acid transporter 1; MS, multiple sclerosis; mTOR, mechanistic target of rapamycin; OXPHOS, oxidative phosphorylation; PD‐1⁺, programmed cell death protein 1‐positive; PDHK1, pyruvate dehydrogenase kinase 1; PKM2, pyruvate kinase M2; PPP, pentose phosphate pathway; RA, rheumatoid arthritis; SLE, systemic lupus erythematosus; STAT1, signal transducer and activator of transcription 1; T1D, Type 1 diabetes; TCA, tricarboxylic acid; TKT, transketolase; TNF‐α, tumor necrosis factor‐α; Treg, regulatory T cell; Th1, T helper 1 cell; Th17, T helper 17 cell; 4‐OI, 4‐octyl itaconate; ACh, acetylcholine; AMPK, adenosine monophosphate‐activated protein kinase; B cell, B lymphocyte; CD4⁺, cluster of differentiation 4‐positive; CD8⁺, cluster of differentiation 8‐positive; CIA, collagen‐induced arthritis; ER, endoplasmic reticulum; FA, fatty acid; FRC, fibroblastic reticular cell; IA, indole‐3‐acrylic acid; IAA, indole‐3‐acetic acid; IC, immune complex; IPA, indole‐3‐propionic acid; ISG, interferon‐stimulated gene; L. casei, Lactobacillus casei; L. paracasei, Lactobacillus paracasei; LKB1, liver kinase B1; mTORC1, mechanistic target of rapamycin complex 1; NAD⁺, oxidized nicotinamide adenine dinucleotide; RNP, ribonucleoprotein; T cell, T lymphocyte; TNF, tumor necrosis factor. Created in BioRender. L, K. (2026) https://BioRender.com/vllki2v.

Immunometabolism focuses on how metabolic pathways and their metabolites regulate immune‐cell development, differentiation, and effector function, and how intrinsic and extrinsic signals, in turn, reshape cellular metabolic states [21]. During activation and differentiation, immune cells undergo marked metabolic reprogramming to satisfy the energetic and biosynthetic requirements of proliferation and effector responses [22, 23]. These metabolic adaptations are not simply passive outcomes of immune activation, but actively influence immune‐cell fate and function [23]. Accumulating evidence indicates that immunometabolic reprogramming may also contribute to heterogeneity in ADs [24, 25]. Different autoimmune disorders, and even distinct immune or stromal cell populations within a single disease, can show selective dependence on particular metabolic pathways [25, 26]. These disease‐ and cell‐specific metabolic preferences may influence inflammatory persistence, tissue injury, and disease progression [27, 28]. Immunometabolic rewiring may not simply be a uniform consequence of autoimmune inflammation, but may also help define pathogenic states that vary by disease, cell type, and tissue context [26, 28]. In this section, we summarize the ways in which major immunometabolic programs contribute to divergent pathogenic states across ADs (Figure 1).

### 2.1 Glycolysis

Glycolysis represents a major metabolic program involved in immune‐cell activation. In ADs, increased glycolytic activity supports different pathogenic programs depending on cell type and disease context.

In CD4^+^ T cells, glycolytic reprogramming is closely linked to inflammatory lineage commitment [29]. Mechanistic target of rapamycin (mTOR)–hypoxia‐inducible factor 1α (HIF‐1α)‐driven glycolysis biases T‐cell fate toward pathogenic T helper 17 (Th17) rather than Treg differentiation, representing one glycolysis‐dependent source of heterogeneity in autoimmune inflammation [29, 30]. Consistent with this, pyruvate dehydrogenase kinase 1 (PDHK1) maintains glycolytic dependence and pathogenic differentiation in Th17 cells, while its inhibition promotes Treg generation and suppresses experimental autoimmune encephalomyelitis (EAE) progression [24]. This glycolysis‐dependent shift in the Th17/Treg balance may contribute to autoimmune pathology [24, 29]. Autoreactive CD4^+^ T cells in experimental T1D also show glycolytic dependence; inhibition of this pathway induces functional and metabolic exhaustion and reduces their pathogenicity [31].

Glycolytic activation appears to amplify inflammation in macrophages and pathogenic B‐cell subsets in RA [27, 32, 33]. Pyruvate kinase M2 (PKM2) is upregulated in macrophages in experimental arthritis and promotes tumor necrosis factor (TNF)‐α and interleukin (IL)‐1β production through signal transducer and activator of transcription 1 (STAT1) signaling, whereas RA patient‐derived macrophages show increased glycolytic and oxidative metabolic activity that is regulated in part by signal transducer and activator of transcription 3 (STAT3) signaling; nicotinamide phosphoribosyltransferase (NAMPT) inhibition also reduces STAT3 expression and activation, supporting functional interplay between NAMPT and STAT3 in regulating the hyper‐inflammatory phenotype, which may contribute to synovial inflammation [27, 32]. Programmed cell death protein 1‐positive (PD‐1^+^) B cells in RA exhibit enhanced glycolytic capacity, dependence on glucose uptake, and a proinflammatory phenotype, with increased IL‐1β and granulocyte–macrophage colony‐stimulating factor (GM‐CSF) expression and enriched inflammatory gene signatures including TNF, IL‐6, and IL‐1β [33]. Together with the observation that RA B cells maintain matrix metalloproteinase 9 (MMP‐9), TNF, and IL‐6 expression under hypoxic conditions that mimic the inflamed joint, these findings suggest that glycolytic PD‐1^+^ B cells may contribute to RA pathogenesis by amplifying local inflammatory and tissue‐destructive responses [33].

Across ADs, glycolysis may contribute to heterogeneity through at least three main outputs: Th17‐biased inflammatory differentiation in T‐cell‐mediated diseases, hyperinflammatory macrophage activation and pathogenic glycolytic B‐cell activity in RA, and pathogenic activity of autoreactive CD4^+^ T cells in experimental T1D [27, 29, 31, 33]. Thus, glycolysis may function as a pathogenic driver or disease‐associated metabolic feature in different autoimmune settings.

### 2.2 Oxidative Phosphorylation (OXPHOS)

OXPHOS has context‐dependent roles in immune activation and cell survival. In ADs, OXPHOS dysregulation may reflect immune‐cell mitochondrial stress or changes in stromal‐cell bioenergetics. OXPHOS dysregulation in SLE appears to contribute to cell‐type‐specific pathogenic outputs rather than a single uniform injury pathway. Studies have shown that memory B cells from patients with SLE exhibit an OXPHOS‐ and mitochondrial‐dysfunction‐related signature associated with organ damage [25]. Mechanistically, mitochondrial respiration can also support type I interferon (IFN‐I) signaling in CD4^+^ T cells: metformin, an inhibitor of mitochondrial respiratory complex I, reduces STAT1 phosphorylation induced by interferon‐α (IFN‐α), STAT1 binding to IFN‐I‐stimulated response elements, and downstream IFN‐I‐stimulated gene expression, whereas additional respiratory‐chain inhibitors similarly suppress IFN‐I‐stimulated gene expression [34]. These findings suggest that OXPHOS abnormalities may shape SLE heterogeneity by linking B‐cell mitochondrial dysfunction with organ‐damage‐associated signatures and by supporting IFN‐I‐responsive inflammatory programs in T cells. In RA, OXPHOS abnormalities are also evident in local stromal cells. Fibroblast‐like synoviocytes (FLSs) from patients with established RA show reduced basal and maximal mitochondrial respiration, whereas FLSs from both individuals at risk of RA and patients with established disease show impaired mitochondrial long‐chain fatty acid β‐oxidation, suggesting that stromal‐cell metabolic dysfunction may occur before clinical arthritis and contribute to synovial pathology [26]. Overall, the effects of OXPHOS abnormalities vary across ADs. These abnormalities are better described as being associated with memory B‐cell mitochondrial dysfunction, organ damage, and CD4^+^ T‐cell IFN‐I responsiveness in SLE, whereas in RA they are associated with FLS bioenergetic dysfunction and preclinical stromal‐cell metabolic alterations.

### 2.3 Lipid Metabolism

Lipid metabolism is another axis along which autoimmune phenotypes diverge, particularly through fatty acid synthesis and fatty acid oxidation. Unlike glycolysis, which often marks inflammatory activation more broadly, lipid programs appear to affect lineage choice and tissue‐destructive behavior in a more disease‐specific manner. In T cells, lipid metabolism is closely tied to the choice between inflammatory and regulatory differentiation. Through acetyl‐coenzyme A carboxylase 1 (ACC1), de novo fatty acid synthesis favors Th17 differentiation while limiting Treg generation; conversely, T‐cell‐specific ACC1 deficiency suppresses Th17 responses and ameliorates EAE [35]. Thus, enhanced lipid synthesis is better interpreted as a metabolic bias that can favor pathogenic T‐cell programming in autoimmune settings. Lipid remodeling is not confined to one cell type in RA; several cellular compartments appear to participate in disease progression. For example, CD8^+^ T cells from patients with RA show altered fatty acid metabolism, including increased fatty acid transporter expression and fatty acid uptake, and in vitro pharmacological blockade of fatty acid metabolism reduces their proinflammatory effector functions [36]. In parallel, leptin‐driven fatty acid oxidation promotes FLS proliferation, inflammatory cytokine production, and angiogenic potential in vitro, thereby potentially contributing to synovial pathology [37]. Lipid metabolism also supports tissue destruction at the bone interface, where carnitine palmitoyltransferase 1A (CPT1A)‐dependent fatty acid oxidation promotes osteoclast precursor fusion and contributes to bone destruction [38]. Together, these findings suggest that lipid metabolism in RA is linked not only with immune activation but also with stromal and osteoclast‐mediated tissue damage. By contrast, in SLE, lipid metabolism supports autoreactive humoral immunity. Acetylcholine derived from splenic fibroblastic reticular cells increases B‐cell lipid uptake and mitochondrial metabolism through CD36, thereby promoting autoreactive B‐cell responses, whereas inhibition of fatty acid oxidation alleviates lupus‐like disease [39]. This finding suggests that enhanced lipid metabolism provides a metabolic basis for pathogenic B‐cell activation in SLE. Lipid metabolic dysregulation illustrates how the same metabolic category can contribute to different pathological outputs depending on cellular context, including pathogenic T‐cell differentiation in neuroinflammation, stromal and osteoclast‐mediated tissue destruction in RA, and support of autoreactive B cells in SLE. These differences need to be considered when lipid‐metabolism‐directed therapies are developed.

### 2.4 Amino Acid Metabolism

Amino acid metabolism regulates immune‐cell activation, differentiation, and inflammatory persistence, with glutamine‐ and tryptophan‐related pathways being particularly relevant to ADs. Glutamine metabolism supports pathogenic T‐cell differentiation in mouse models: alanine–serine–cysteine transporter 2 (ASCT2)‐mediated glutamine uptake promotes mTOR complex 1 (mTORC1) activation and the differentiation of T helper 1 (Th1) and Th17 cells [40], while glutaminase (GLS)‐dependent glutaminolysis sustains IL‐17 production and Th17 differentiation, and T‐cell‐specific GLS deficiency attenuates Th17‐driven inflammatory pathology [41].

Increased glutamine use is tied to mitochondrial activation and plasmablast differentiation *in* *vitro*, while B‐cell mitochondrial hyperpolarization is associated with plasmablast frequency and disease activity in SLE [42]. Accordingly, blocking glutaminolysis improves lupus‐like disease in MRL/lpr mice and is accompanied by changes in inflammatory signaling and lymphocyte subsets [43]. In RA T cells, insufficient mitochondrial aspartate generation disrupts nicotinamide adenine dinucleotide regeneration and promotes transmembrane TNF biosynthesis, a mechanism that can amplify synovial inflammation [44]. Other amino acid‐related processes also enter the inflammatory circuit: L‐type amino acid transporter 1 (LAT1) enables T‐cell activation in the inflamed joints of arthritic mice, whereas modulation of gut microbiota–tryptophan–aryl hydrocarbon receptor (AhR) signaling can attenuate arthritis progression in rats with collagen‐induced arthritis [45, 46]. Overall, amino acid metabolism contributes to autoimmune heterogeneity through its support of pathogenic T‐cell differentiation, plasmablast differentiation, inflammatory mediator production, and context‐dependent immune activation. These findings indicate that amino acid metabolic pathways are not uniformly pathogenic and instead shape distinct disease programs across autoimmune settings.

### 2.5 Tricarboxylic Acid (TCA) Cycle Metabolites

TCA‐cycle metabolites may contribute to AD heterogeneity by acting as signaling molecules that modulate inflammatory and regulatory programs. In autoimmune settings, itaconate mainly functions as an anti‐inflammatory metabolite. Mechanistically, itaconate and its cell‐permeable derivatives can alkylate Kelch‐like ECH‐associated protein 1 (KEAP1) cysteine residues to stabilize nuclear factor erythroid 2‐related factor 2 (NRF2) and activate antioxidant and anti‐inflammatory transcriptional programs [47], and itaconate can inhibit ten‐eleven translocation (TET) DNA dioxygenases by competing with α‐ketoglutarate in α‐ketoglutarate‐dependent dioxygenase reactions [48]; additionally, the cell‐permeable derivative 4‐octyl itaconate modifies nucleotide‐binding oligomerization domain, leucine‐rich repeat, and pyrin domain‐containing protein 3 (NLRP3), disrupts the NLRP3–never in mitosis A‐related kinase 7 (NEK7) interaction, and blocks NLRP3 inflammasome activation [49]. These mechanisms are consistent with protective effects of itaconate or its derivatives in AD models: itaconate ameliorates EAE by modulating pathogenic T‐cell imbalance [50], reduces FLS proliferation, migration, arthritis, and bone erosion in RA models [51], and the itaconate derivative 4‐octyl itaconate attenuates established murine lupus, particularly renal injury and immune dysregulation [52]. These findings indicate that itaconate can restrain autoimmune inflammation by suppressing pathogenic immune programs and limiting inflammatory tissue responses. By contrast, succinate is best presented here as a proinflammatory macrophage‐associated TCA‐cycle signal with plausible relevance to autoimmune inflammatory niches. In inflammatory macrophage models, succinate accumulates partly through glutamine‐dependent anaplerosis and the gamma‐aminobutyric acid (GABA) shunt, stabilizes HIF‐1α, and enhances IL‐1β expression [53]; succinate oxidation through succinate dehydrogenase also drives mitochondrial reactive oxygen species (ROS) production and supports inflammatory macrophage activation [54]. Therefore, succinate‐related pathways may provide a mechanistic basis for cytokine amplification in autoimmune tissues, although the evidence cited here is less disease‐specific than the autoimmune‐model evidence for itaconate. Overall, itaconate and succinate illustrate how TCA‐cycle metabolites may shape AD programs in opposing directions: itaconate acts as a metabolic brake on inflammatory pathology, whereas succinate promotes inflammatory amplification through HIF‐1α‐, IL‐1β‐, and macrophage‐associated mechanisms.

### 2.6 Pentose Phosphate Pathway (PPP)

The PPP links glucose use to redox control, nucleotide synthesis, and immune regulation [55]. Evidence from several autoimmune settings further points to PPP dysregulation as a contributor to disease pathogenesis [28, 56, 57]. Different parts of the pathway do not act in the same direction; their effects vary between immune tolerance and inflammatory activation. An intact nonoxidative PPP in Treg cells is needed to maintain suppressive function and restrain autoimmune responses [56]. In an apoptotic‐cell‐induced murine SLE model and related macrophage efferocytosis assays, pharmacological activation of the PPP reduced macrophage clearance of apoptotic cells, enhanced inflammatory responses during efferocytosis, and exacerbated lupus‐like manifestations [57]. In MS, increased PPP engagement in CD8^+^ T cells is associated with a pathogenic program because experimental PPP inhibition reduces glucose uptake, proliferation, proinflammatory cytokine production, and antigen‐specific neuronal injury [28]. The PPP therefore should not be treated as uniformly protective or uniformly pathogenic. This context dependence makes the PPP a useful example of why the same metabolic pathway can be assigned different roles across AD programs.

### 2.7 Summary

Taken together, metabolic reprogramming is best interpreted as a set of links between nutrient use, metabolite signaling, immune‐cell fate, and tissue injury, rather than as a single pathogenic program. For glycolysis, the relevant outputs include Th17‐skewed T‐cell responses, macrophage‐driven synovial inflammation, accumulation of pathogenic B cells, and maintenance of the pathogenic function of autoreactive CD4^+^ T cells. OXPHOS‐related abnormalities point more toward immune‐cell stress in SLE and bioenergetic dysfunction of stromal cells in RA. Lipid metabolism maps onto pathogenic T‐cell differentiation, stromal and osteoclast‐mediated tissue damage in RA, and autoreactive humoral immunity in SLE. Amino acid metabolism is linked to pathogenic T‐cell differentiation, plasmablast differentiation, inflammatory mediator production, and context‐dependent regulation involving glutamine, aspartate, amino acid transport, and tryptophan‐related pathways. TCA‐cycle metabolites, especially itaconate and succinate, form opposing inflammatory rheostats; the PPP can support immune tolerance or inflammatory amplification depending on context. Together, these observations refine the view that immunometabolic dysregulation does not follow a uniform pattern across ADs, but instead represents an important mechanism shaping distinct pathogenic programs in a disease‐, cell‐, and tissue‐specific manner (Figure 1).

Immunometabolic heterogeneity provides a framework for connecting metabolic mechanisms with clinically relevant disease features. Cell‐ and tissue‐specific metabolic states may indicate early pathogenic immune activation or risk‐associated tissue alterations, complement conventional inflammatory measures in assessing disease activity, and help explain why particular patients develop dominant lymphocyte‐, macrophage‐, stromal‐, or osteoclast‐related pathology. Because metabolic dependencies differ across immune and tissue‐resident compartments, they may also support mechanism‐informed patient stratification and therapeutic prioritization. These implications represent conceptual links between metabolism and clinical utility.

## 3 Epigenetic Remodeling: Stabilizing Autoimmune Cell States and Tissue‐Specific Injury

**FIGURE 2:**
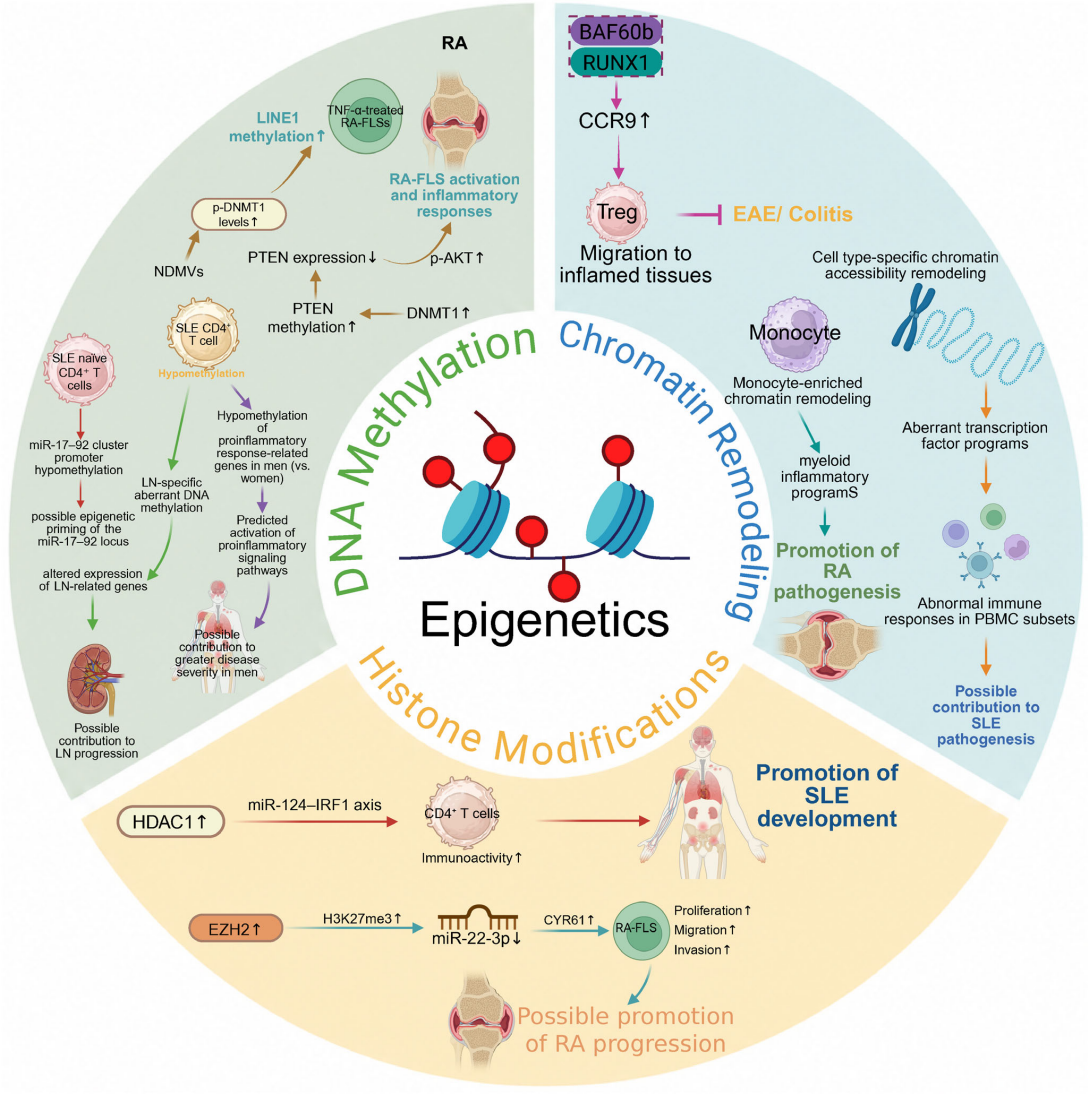
Epigenetic mechanisms stabilizing autoimmune cell states. DNA methylation, histone modifications, and chromatin remodeling regulate immune‐cell activation, tissue‐resident stromal phenotypes, organ‐specific injury, and persistent inflammatory programs. These mechanisms help explain how environmental and inflammatory cues are converted into stable pathogenic or regulatory states. p‐AKT, phosphorylated protein kinase B; BAF60b, BRG1/BRM‐associated factor 60b; CCR9, C‐C motif chemokine receptor 9; CYR61, cysteine‐rich angiogenic inducer 61; DNA, deoxyribonucleic acid; DNMT1, DNA methyltransferase 1; EZH2, enhancer of zeste homolog 2; H3K27me3, histone H3 lysine 27 trimethylation; HDAC1, histone deacetylase 1; IRF1, interferon regulatory factor 1; LINE1, long interspersed nuclear element 1; NDMVs, neutrophil‐derived microvesicles; PBMC, peripheral blood mononuclear cells; p‐DNMT1, phosphorylated DNA methyltransferase 1; PTEN, phosphatase and tensin homolog; RA, rheumatoid arthritis; RA‐FLS, rheumatoid arthritis fibroblast‐like synoviocyte; RUNX1, runt‐related transcription factor 1; SLE, systemic lupus erythematosus; Treg, regulatory T cell; CD4⁺, cluster of differentiation 4‐positive; EAE, experimental autoimmune encephalomyelitis; LN, lupus nephritis; miR‐17–92, microRNA‐17–92 cluster; miR‐22‐3p, microRNA‐22‐3p; miR‐124, microRNA‐124; RA‐FLSs, rheumatoid arthritis fibroblast‐like synoviocytes; T cell, T lymphocyte; TNF‐α, tumor necrosis factor‐α. Created in BioRender. L, K. (2026) https://BioRender.com/3pp5rl1.

Epigenetic regulation, including DNA methylation, histone modification, and chromatin remodeling, shapes gene expression without altering DNA sequence. In ADs, these mechanisms stabilize immune‐cell states and help explain why shared genetic susceptibility can still lead to marked differences in organ involvement and clinical phenotype. Studies of monozygotic twins discordant for SLE and subsequent integrative analyses indicate that lupus‐associated DNA methylation alterations may be shaped largely by nongenetic influences, potentially including environmental exposure, rather than by inherited variants alone [58, 59]. Accordingly, the role of epigenetics in autoimmune heterogeneity is not limited to disease‐associated gene dysregulation, but extends to stabilizing distinct disease states across patients, tissues, and immune‐cell subsets. In this section, we discuss how DNA methylation, histone modifications, and chromatin remodeling shape heterogeneity in ADs (Figure 2). Instead of presenting these layers as separate mechanisms, we focus on their role in explaining differences in sex bias, organ‐specific injury, tissue‐resident cell states, and the persistence of pathogenic or regulatory immune programs.

### 3.1 DNA Methylation

DNA methylation is one of the better‐defined epigenetic changes in ADs. It provides a useful example of how an epigenetic mark may relate to immune‐cell state, inflammatory output, and organ involvement in SLE. One recurring epigenetic feature of SLE is hypomethylation in naïve CD4^+^ T cells, particularly at interferon‐regulated genes, where hypomethylation precedes transcriptional activity and is consistent with epigenetic priming [59]. Lupus‐associated methylation signatures seem to reflect nongenetic influences more than genetic background alone [59]. In proliferative lupus nephritis, CD4^+^ T cells show distinct differentially methylated cytosine‐phosphate‐guanine (CpG) positions and corresponding transcriptional changes. These findings support an association between DNA methylation and renal‐involvement heterogeneity in SLE [60]. Sex‐related differences are also visible at the methylation level. Male patients with SLE show proinflammatory DNA methylation patterns in CD4^+^ T cells compared with female patients with SLE, involving genes related to apoptosis, T‐cell function, and inflammatory signaling pathways; these patterns may partly explain sex‐associated differences in disease severity [61]. As a related readout of transposable‐element activity, whole‐blood ribonucleic acid (RNA) sequencing (RNA‐seq) data from SLE further show a strong association between hyperactivated transposon expression, interferon signatures, and immune activation, with neutrophils proposed as a major source [62]. DNA methylation therefore appears to mark more than disease presence alone in SLE; it relates to sex bias and renal involvement, whereas transposon‐expression profiles provide an additional whole‐blood readout associated with interferon activity and immune activation.

DNA methylation evidence is particularly relevant to tissue‐resident FLSs within the synovial inflammatory microenvironment in RA. At the mechanistic level, upregulation of DNA methyltransferase 1 (DNMT1) in RA FLSs (RA‐FLSs) is associated with increased DNA methylation of the phosphatase and tensin homolog (PTEN) gene, lower PTEN expression, and altered protein kinase B (AKT) signaling; this axis may promote FLS activation and synovial pathology [63]. Another in vitro study using human FLSs showed that neutrophil‐derived microvesicles generated after TNF‐α stimulation were internalized by FLSs and counteracted TNF‐α‐associated loss of long interspersed nuclear element 1 (LINE‐1) methylation, with this effect accompanied by changes in phosphorylated DNMT1 expression [64]. These findings in RA move DNA methylation beyond circulating immune cells and place it within pathogenic tissue niches. Aberrant DNA methylation may be one way in which inflammatory exposure contributes to FLS activation. Through these effects, DNA methylation may help account for sex‐related differences and renal involvement in SLE, as well as FLS activation and local inflammatory responses in RA.

### 3.2 Histone Modifications

Histone modifications influence chromatin accessibility and transcriptional output, and in ADs, this regulation can stabilize either inflammatory or regulatory cell states. In immune cells, these modifications affect not only activation status but also the direction of effector differentiation. Histone deacetylase 1 (HDAC1) is overexpressed in CD4^+^ T cells from patients with SLE and binds the promoter of microRNA‐124 (miR‐124), reducing miR‐124 expression; because miR‐124 represses interferon regulatory factor 1 (IRF1) expression, HDAC1‐mediated miR‐124 inhibition increases IRF1 expression and enhances CD4^+^ T‐cell immunoactivity [65]. This type of regulation is particularly relevant when excessive T‐cell activation and persistent immune dysregulation are central features of the disease.

Histone modifications are also involved in differences between tissue‐resident pathogenic cells and immunosuppressive cell populations. Enhancer of zeste homolog 2 (EZH2), the catalytic component of polycomb repressive complex 2 (PRC2), is upregulated in RA‐FLSs and promotes their proliferation, migration, and invasion by increasing histone H3 lysine 27 trimethylation (H3K27me3) at the miR‐22‐3p promoter, repressing miR‐22‐3p, and thereby increasing cysteine‐rich 61 (CYR61) expression [66]. In Tregs, IKAROS family zinc finger 1 (IKZF1) binds forkhead box protein P3 (FOXP3) through its exon 5 and forms a repressive FOXP3–IKZF1–IKAROS family zinc finger 3 (IKZF3) complex; the FOXP3–IKZF1 complex associates with the nucleosome remodeling and deacetylase (NuRD) complex to restrict chromatin accessibility and exclude coactivators such as p300 and transcription factors such as nuclear factor of activated T cells 1 (NFAT1) from FOXP3 target loci; deletion of IKZF1 exon 5 derepresses interferon‐γ (IFN‐γ) and other effector programs, destabilizes FOXP3 expression, impairs Treg suppressive function, and causes systemic autoimmune inflammation [67]. These examples make the context dependence clear: histone‐modifying enzymes can support destructive stromal‐cell phenotypes in RA‐FLSs, whereas disruption of FOXP3‐associated chromatin‐repressive complexes weakens immune restraint in Tregs. Overall, histone modifications contribute to autoimmune heterogeneity by helping stabilize distinct inflammatory or regulatory cell states. Their significance lies not simply in the regulation of individual genes, but in their ability to reinforce pathogenic phenotypes and dysfunctional regulatory states in both immune cells and tissue‐resident cells.

### 3.3 Chromatin Remodeling

Through adenosine triphosphate (ATP)‐dependent complexes such as switch/sucrose nonfermentable (SWI/SNF), chromatin remodeling changes nucleosome positioning and DNA accessibility. In ADs, its importance lies more in shaping broad regulatory landscapes than in regulating individual genes, thereby influencing which cell populations respond to inflammatory cues and which tissues maintain pathogenic immune interactions. In RA synovium, paired single‐cell RNA‐seq and assay for transposase‐accessible chromatin with sequencing (ATAC‐seq) analyses identify FLS states with distinct inflammatory, invasive, and tissue‐remodeling programs, and link this FLS heterogeneity to state‐specific chromatin accessibility shaped by local cytokine exposure [68].

Single‐cell chromatin accessibility studies in SLE, together with ATAC‐seq analyses in RA, have revealed marked cell type‐specific regulatory remodeling in SLE and RA. Single‐cell ATAC‐seq (scATAC‐seq) has revealed distinct accessible chromatin patterns and abnormal transcription factor programs across peripheral blood immune subsets in SLE [69]. Among the examined peripheral immune‐cell types, RA‐associated chromatin accessibility changes are most prominent in monocytes, highlighting the contribution of myeloid‐cell regulatory landscapes to RA pathogenesis [70]. These findings suggest that chromatin remodeling helps explain disease‐associated differences in immune‐cell states and cell‐type involvement.

Additional studies further connect enhancer remodeling and SWI/SNF‐dependent chromatin remodeling with disease‐specific CD4^+^ T‐cell regulation and tissue‐targeted immune suppression. Cross‐disease analyses of CD4^+^ T cells have identified disease‐specific enhancer remodeling and downstream regulatory circuits across SLE, psoriasis, juvenile idiopathic arthritis, and Graves’ disease [71]. In addition, the SWI/SNF‐related matrix‐associated actin‐dependent regulator of chromatin subfamily D member 2 (SMARCD2; also known as BAF60b), a component of the SWI/SNF complex, cooperates with runt‐related transcription factor 1 (RUNX1) to maintain C‐C chemokine receptor type 9 (CCR9) expression on Tregs and thereby supports their migration to inflamed tissues. SMARCD2 deficiency impairs Treg‐cell migration to inflamed tissues and exacerbates inflammation in EAE and colitis models [72]. These mechanisms illustrate that chromatin remodeling can control whether regulatory cells localize to the appropriate tissue context to exert suppressive function. Overall, chromatin remodeling shapes autoimmune heterogeneity by defining accessible regulatory landscapes, enhancer activity, and migratory competence. It therefore links immune‐cell identity to tissue distribution and helps determine where and how disease is expressed.

### 3.4 Summary

Taken together, epigenetic dysregulation contributes to autoimmune heterogeneity by shaping disease‐associated transcriptional states in both immune cells and tissue‐resident cells. Importantly, epigenetic changes may also provide a mechanistic bridge between environmental or inflammatory exposure and immune‐cell or stromal‐cell reprogramming. Together, DNA methylation, histone modification, and chromatin remodeling help explain why patients with similar genetic susceptibility can still diverge in inflammatory burden, organ involvement, and dominant pathogenic cell states. In this sense, epigenetic regulation may help explain how immune perturbations contribute to disease‐associated phenotypes.

Epigenetic remodeling is important because it may preserve a molecular memory of environmental exposure, inflammatory history, and tissue‐specific immune activation. DNA methylation, histone‐modification, and chromatin‐accessibility patterns may therefore help explain stable disease subsets, organ‐involvement tendencies, and persistent disease‐activity states. However, the translational potential of these links requires further validation.

## 4 Posttranscriptional Regulation in Shaping the Heterogeneity of ADs

Posttranscriptional regulation operates at the RNA level and changes gene expression by altering RNA splicing, chemical modification, stability and decay, transport, translation, and editing. Through these mechanisms, cells can generate diverse RNA and protein outputs from the same genomic sequence, allowing gene‐expression programs to be adjusted at the RNA level.

**FIGURE 3:**
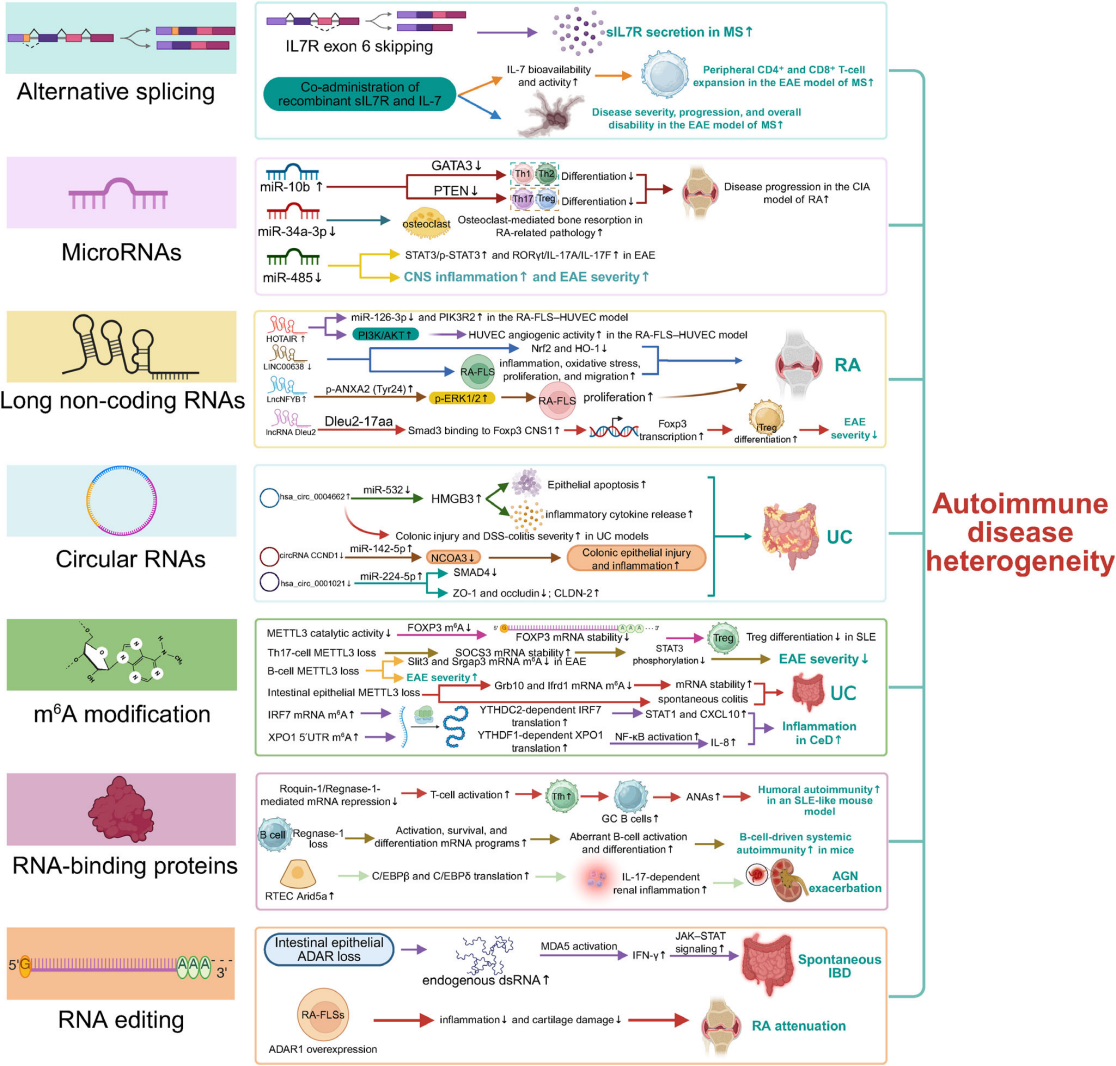
Posttranscriptional regulation in autoimmune heterogeneity. Alternative splicing, microRNAs, long noncoding RNAs, circular RNAs, m^6^A modification, RNA‐binding proteins, and RNA editing reshape transcript fate, inflammatory signaling, immune‐cell differentiation, epithelial barrier function, and target‐organ injury. SLE, systemic lupus erythematosus; MS, multiple sclerosis; RA, rheumatoid arthritis; UC, ulcerative colitis; CeD, celiac disease; IL7R, interleukin‐7 receptor; sIL7R, soluble interleukin‐7 receptor; Th1, T helper 1 cell; Th2, T helper 2 cell; Th17, T helper 17 cell; Treg, regulatory T cell; Tfh, T follicular helper cell; GATA3, GATA binding protein 3; PTEN, phosphatase and tensin homolog; STAT3, signal transducer and activator of transcription 3; RA‐FLS, rheumatoid arthritis fibroblast‐like synoviocyte; RA‐FLSs, rheumatoid arthritis fibroblast‐like synoviocytes; HUVEC, human umbilical vein endothelial cell; PI3K, phosphoinositide 3‐kinase; AKT, protein kinase B; Nrf2, nuclear factor erythroid 2‐related factor 2; HO‐1, heme oxygenase 1; p‐ANXA2 (Tyr24), annexin A2 phosphorylated at tyrosine 24; p‐ERK1/2, phosphorylated extracellular signal‐regulated kinases 1 and 2; FOXP3 and Foxp3, forkhead box P3; HMGB3, high mobility group box 3; NCOA3, nuclear receptor coactivator 3; EAE, experimental autoimmune encephalomyelitis; m^6^A, N^6^‐methyladenosine; SOCS3, suppressor of cytokine signaling 3; IRF7, interferon regulatory factor 7; NF‐κB, nuclear factor kappa B; MDA5, melanoma differentiation‐associated protein 5; JAK–STAT, Janus kinase‐signal transducer and activator of transcription; dsRNA, double‐stranded RNA; 5′UTR, 5′ untranslated region; ADAR, adenosine deaminase acting on RNA; ADAR1, adenosine deaminase acting on RNA 1; AGN, autoimmune glomerulonephritis; ANAs, antinuclear antibodies; Arid5a, AT‐rich interaction domain 5A; B cell, B lymphocyte; C/EBPβ, CCAAT/enhancer‐binding protein beta; C/EBPδ, CCAAT/enhancer‐binding protein delta; CCND1, cyclin D1; CD4⁺, cluster of differentiation 4‐positive; CD8⁺, cluster of differentiation 8‐positive; CIA, collagen‐induced arthritis; circRNA, circular RNA; CLDN‐2, claudin‐2; CNS, central nervous system; CNS1, conserved noncoding sequence 1; CXCL10, C‐X‐C motif chemokine ligand 10; Dleu2, deleted in lymphocytic leukemia 2; Dleu2‐17aa, Dleu2‐encoded 17‐amino‐acid peptide; DSS, dextran sulfate sodium; GC, germinal center; Grb10, growth factor receptor‐bound protein 10; HOTAIR, HOX transcript antisense intergenic RNA; hsa_circ_0001021, Homo sapiens circular RNA 0001021; hsa_circ_0004662, Homo sapiens circular RNA 0004662; IBD, inflammatory bowel disease; IFN‐γ, interferon‐γ; Ifrd1, interferon‐related developmental regulator 1; IL‐7, interleukin‐7; IL‐8, interleukin‐8; IL‐17, interleukin‐17; IL‐17A, interleukin‐17A; IL‐17F, interleukin‐17F; iTreg, inducible regulatory T cell; LINC00638, long intergenic non‐protein‐coding RNA 638; LncNFYB, long noncoding RNA NFYB; lncRNA, long noncoding RNA; METTL3, methyltransferase‐like protein 3; miR‐10b, microRNA‐10b; miR‐34a‐3p, microRNA‐34a‐3p; miR‐126‐3p, microRNA‐126‐3p; miR‐142‐5p, microRNA‐142‐5p; miR‐224‐5p, microRNA‐224‐5p; miR‐485, microRNA‐485; miR‐532, microRNA‐532; mRNA, messenger RNA; p‐STAT3, phosphorylated signal transducer and activator of transcription 3; PIK3R2, phosphoinositide‐3‐kinase regulatory subunit 2; RNA, ribonucleic acid; RORγt, retinoic acid receptor‐related orphan receptor gamma t; RTEC, renal tubular epithelial cell; Slit3, slit guidance ligand 3; Smad3, SMAD family member 3; SMAD4, SMAD family member 4; Srgap3, SLIT‐ROBO Rho GTPase‐activating protein 3; STAT1, signal transducer and activator of transcription 1; T cell, T lymphocyte; XPO1, exportin 1; YTHDC2, YTH domain‐containing protein 2; YTHDF1, YTH domain‐containing family protein 1; ZO‐1, zonula occludens‐1. Created in BioRender. L, K. (2026) https://BioRender.com/0lw0yxv.

Because ADs differ markedly in phenotype and tissue involvement, RNA‐level regulation is one plausible source of this variation. Alternative splicing, ncRNA regulation, RNA modification, and RNA editing act on immune‐cell function, inflammatory responses, and target‐organ injury. These RNA‐level changes may help explain why phenotypes differ both between ADs and within the same disease. This section therefore considers RNA‐level regulation through several interrelated forms (Figure 3), including changes in transcript isoforms, ncRNA‐mediated regulatory networks, RNA modification patterns, RNA stability and translation control, and endogenous RNA sensing. Where relevant, these RNA‐level mechanisms are also discussed in relation to metabolic and epigenetic programs.

### 4.1 Alternative Splicing

Alternative splicing is one of the main RNA‐level mechanisms through which eukaryotic cells diversify gene output [73]. By selecting alternative splice sites, retaining introns, or including or skipping exons in precursor messenger RNAs (mRNAs), one gene can give rise to RNA and protein isoforms with different structures and functions, increasing transcriptomic and proteomic diversity [73, 74]. Alternative splicing events are regulated through the combined action of cis‐regulatory elements and trans‐acting splicing factors [73].

Aberrant alternative splicing has been reported in several ADs and is increasingly linked to their pathogenic programs. Xu et al. [73] used patient transcriptomic data to perform a genome‐wide analysis of alternative splicing and identified multiple dysregulated splicing events in peripheral blood in SLE. Changes in key splicing factors were associated with abnormal splicing of immune‐related genes. They were also linked to altered immune‐cell infiltration patterns. Sun et al. [74] further examined RNA‐seq data from T cells and B cells in patients with SLE and found widespread abnormalities in intron retention (IR). Distinct IR patterns separated SLE T‐cell samples into subgroups, suggesting that splicing‐program variation may contribute to the molecular heterogeneity of SLE.

At immune‐regulatory loci, alternative splicing can alter autoimmune pathology by changing the functional isoforms produced from the same gene. Galarza‐Muñoz et al. [75] studied alternative splicing of interleukin‐7 receptor (IL7R) exon 6 and showed that this event controls production of soluble interleukin‐7 receptor (sIL7R), while risk‐associated splicing patterns increase sIL7R expression, strengthen interleukin‐7 (IL‐7) signaling, and are associated with greater susceptibility to MS. Population‐scale genetic analyses have also brought alternative splicing into the discussion of genetic heterogeneity. Using single‐cell RNA‐seq data from the Asian Immune Diversity Atlas, Tian et al. [76] identified cell type‐specific splicing quantitative trait loci (sQTLs) across 19 immune‐cell types. Cis‐sQTL effects were enriched in the heritability of autoimmune and inflammatory diseases, and sQTL signals colocalized with loci associated with these diseases, indicating that autoimmune risk loci may affect splicing in a cell type‐specific manner. Alternative splicing may therefore contribute to autoimmune heterogeneity at more than one level: it changes the structure and function of immune‐related transcripts and produces splicing programs that differ across immune‐cell types.

### 4.2 MicroRNAs (miRNAs)

miRNAs are small endogenous single‐stranded ncRNAs. They usually bind target mRNAs through sequences in the 3′ untranslated region (3′ UTR), leading to mRNA degradation or translational repression.

Aberrant miRNA expression intersects with two major disease features in RA: immune‐cell differentiation and bone‐destruction programs. This may help explain why distinct pathological subtypes emerge. Tu et al. [77] reported upregulation of miR‐10b in CD4^+^ T cells from patients with RA. By targeting GATA3 and PTEN, miR‐10b disrupted the balance among CD4^+^ T‐cell subsets, promoted Th1 and Th17 differentiation, suppressed T helper 2 (Th2)‐ and Treg‐associated programs, and ultimately aggravated experimental arthritis. Dong et al. [78] profiled miRNAs in osteoclasts derived from peripheral blood mononuclear cells (PBMCs) of patients with RA and detected distinct miRNA patterns in erosive versus nonerosive RA. Functional experiments showed that miR‐34a‐3p directly influenced osteoclastic bone resorption, whereas miR‐365b‐3p, miR‐374a‐3p, miR‐511‐3p, and miR‐193b‐3p were linked to the erosive phenotype. These findings place miRNA abnormalities in two RA‐related processes: amplification of inflammation and separation of erosive from nonerosive phenotypes.

In MS‐related autoimmune neuroinflammation, disease severity is also linked to miRNA control of pathogenic Th17 programs. Xue et al. [79] found that miR‐485 expression was reduced during EAE. Experimental miR‐485 knockdown relieved repression of STAT3, increased Th17 cell generation and related cytokine expression, and aggravated EAE. Conversely, increased miR‐485 expression suppressed Th17‐associated inflammatory signaling and was associated with improvement of EAE. These results suggest that miRNA abnormalities can push MS‐like inflammation in either direction, aggravating or attenuating tissue injury through effects on pathogenic T‐cell differentiation. miRNA‐related heterogeneity may therefore operate at several points: expression patterns and regulatory axes vary by disease context, and the downstream effects include immune‐cell differentiation, bone resorption, inflammatory amplification, disease activity, organ injury, and differences between pathological subtypes.

### 4.3 Long Noncoding RNAs (lncRNAs)

lncRNAs are transcripts longer than 200 nucleotides that generally do not encode proteins. Their regulatory effects arise through several routes, including miRNA‐related interactions, binding to RNA‐binding proteins (RBPs), and, in some cases, production of functional micropeptides.

In RA, lncRNAs do not act in a single direction in FLSs or related microenvironmental cells. This divergence in regulatory effect may be one source of disease heterogeneity. In an RA‐FLS‐induced human umbilical vein endothelial cell (HUVEC) model, Liu et al. [80] showed that HOX transcript antisense RNA (HOTAIR), a lncRNA, acted through the miR‐126‐3p/PIK3R2 axis. This activated phosphoinositide 3‐kinase (PI3K)/AKT signaling and promoted endothelial proliferation, migration, and tube formation, linking lncRNA HOTAIR to RA‐associated synovial angiogenesis. By contrast, Sun et al. [81] reported downregulation of LINC00638 in RA. Restoring LINC00638 activated the Nrf2/heme oxygenase 1 (HO‐1) pathway and suppressed inflammatory responses, oxidative stress, proliferation, and migration in FLSs. Reduced LINC00638 expression therefore appears to mark a more inflammatory and migratory synovial phenotype in RA. Xiao et al. [82] identified another RA‐associated lncRNA, LncNFYB. By binding ANXA2 and enhancing extracellular signal‐regulated kinases 1 and 2 (ERK1/2) activation, LncNFYB promoted RA‐FLS proliferation, again linking lncRNA regulation to proliferative stromal phenotypes.

The function of lncRNAs is not limited to RNA‐mediated regulation; some lncRNAs also encode functional peptides. Tang et al. [83] showed that lncRNA Dleu2 encodes Dleu2‐17aa, a 17‐amino‐acid micropeptide that promotes inducible Treg formation by enhancing Smad3 binding to conserved noncoding sequence 1 within Foxp3. Loss of Dleu2‐17aa impaired Treg induction and worsened EAE, whereas exogenous supplementation with this micropeptide reduced disease severity. These findings place lncRNAs at two levels of autoimmune regulation: local pathogenic processes regulated by lncRNA‐mediated mechanisms and immune tolerance influenced by lncRNA‐encoded micropeptides. The relevance of lncRNAs in AD comes partly from their tissue specificity, cell type specificity, and mechanistic diversity. Through these features, they are linked to synovial angiogenesis, inflammatory amplification, oxidative stress, and immune tolerance. As a result, dysregulated or pathogenic lncRNA programs may worsen disease processes such as RA synovial angiogenesis and FLS proliferation, whereas protective lncRNA‐derived products such as Dleu2‐17aa can restrain inflammation and alleviate EAE.

### 4.4 Circular RNAs (circRNAs)

circRNAs are endogenous RNAs commonly generated by back‐splicing and characterized by covalently closed circular structures. Because they do not contain a 5′ cap or a 3′ polyadenylated tail, circRNAs are highly resistant to exonuclease‐mediated degradation. At the posttranscriptional level, circRNAs can act by sponging miRNAs or interacting with proteins, including RBPs.

Altered circRNA expression appears to connect genetic risk with peripheral immune‐cell RNA profiles in MS. Cardamone et al. [84] carried out circRNA‐enriched sequencing in PBMCs from patients with MS, identified numerous differentially expressed circRNAs, and found significant associations between multiple MS risk loci and circRNA expression. These findings suggest that genetic risk variants may affect circRNA expression profiles in peripheral immune cells.

In ulcerative colitis (UC), circRNAs do not show a uniform direction of effect. Depending on the circRNA involved, they may influence epithelial inflammation, apoptosis, and barrier function in ways that lead to different pathological outcomes. Qiu et al. [85] reported that hsa_circ_0004662 was upregulated in UC tissues and in LPS‐stimulated colonic epithelial cells. Acting through the miR‐532/HMGB3 axis, it promoted cell apoptosis, inflammatory cytokine release, and tissue injury, and it aggravated colitis in a mouse model. Li et al. [86] reported that circular RNA ATPase class II type 9B (circAtp9b) was induced by lipopolysaccharide stimulation and promoted apoptosis of colonic epithelial cells through PTEN upregulation, suggesting a potential role in epithelial injury and UC progression. In contrast, Xiang et al. [87] showed that circRNA CCND1 was downregulated in UC, and restoring its expression in LPS‐treated Caco‐2 cells reduced cell injury and inflammatory responses through the miR‐142‐5p/NCOA3 axis. Li et al. [88] further found decreased hsa_circ_0001021 expression in UC and showed that this circRNA maintained epithelial barrier‐associated molecules, including zonula occludens 1 (ZO‐1) and occludin, through the miR‐224‐5p/SMAD4 axis. Reduced expression of this circRNA was associated with lower expression of barrier‐associated molecules and greater disease severity. The UC examples show why circRNAs should be interpreted in a context‐specific way. Depending on the specific circRNA and tissue context, they may be linked with either proinflammatory or protective effects in autoimmune‐related inflammation. Because their effects can move in opposite directions, circRNAs form a posttranscriptional layer linked to differences in inflammatory intensity, tissue injury, and disease severity.

### 4.5 N^6^‐Methyladenosine (m⁶A) RNA Modification

m^6^A is the most abundant internal chemical modification in eukaryotic mRNA. It is enriched mainly near stop codons and in 3′ UTRs. m^6^A modification is dynamic and reversible. It is deposited by an m^6^A methyltransferase complex, erased by demethylases, and recognized by m^6^A reader proteins that determine its downstream effects on mRNA splicing, stability, localization, and translation.

Recent studies point to a strongly cell‐dependent effect of m^6^A modification on autoimmune phenotypes. Lu et al. [89] found reduced expression of the methyltransferase METTL3 in CD4^+^ T cells from patients with SLE. Suppression of METTL3 reduced FOXP3 mRNA stability and impaired Treg differentiation, while also promoting CD4^+^ T‐cell activation and altering effector differentiation. The result was a shift in the Treg–effector T‐cell balance, with downstream impairment of immune tolerance and aggravation of the lupus‐like phenotype in a cGVHD mouse model. In contrast, Wang et al. [90] found in EAE that METTL3 deficiency in Th17‐lineage cells increased Socs3 mRNA stability, impaired encephalitogenic Th17 differentiation and infiltration into the central nervous system (CNS), reduced IL‐17A and CCR5 expression, and ultimately attenuated EAE‐associated neuroinflammation. However, Pei et al. [91] further showed that B cell‐specific METTL3 deletion aggravated EAE, with more severe inflammatory infiltration and demyelination, and this phenotype was associated with reduced m^6^A modification of transcripts such as Slit3 and Srgap3 in B cells. These findings show why the same m^6^A regulator cannot be assigned a single disease role. Its effect depends on the immune‐cell type, and this cell specificity helps explain m^6^A‐mediated disease heterogeneity.

m^6^A modification is also relevant to intestinal autoimmune‐related inflammation, particularly through its effects on target‐organ homeostasis. Fang et al. [92] showed that intestinal epithelial cell‐specific deletion of Mettl3 induced spontaneous colitis in mice, accompanied by dysregulation of T‐lymphocyte subsets. Ding et al. [93] reported METTL3 downregulation in the colonic mucosa of patients with UC. Mettl3 loss increased Grb10 and Ifrd1 mRNA stability, inhibited the self‐renewal and differentiation of Lgr5‐positive intestinal stem cells, especially goblet cell differentiation and maturation, and caused epithelial developmental abnormalities with spontaneous inflammation, thereby promoting UC‐relevant epithelial injury. Sebastian‐delaCruz et al. [94] found elevated m^6^A levels in the intestinal mucosa of patients with celiac disease (CeD) and linked m^6^A‐dependent, YTHDC2‐mediated regulation of IRF7 to inflammatory programs in CeD, which may promote mucosal inflammation. Olazagoitia‐Garmendia et al. [95] further showed that CeD‐associated XPO1 risk allele rs3087898‐T enhanced XPO1 translation through an m^6^A‐dependent mechanism and activated nuclear factor kappa B (NF‐κB)‐related inflammatory programs. This finding links m^6^A modification to the way risk alleles may influence the intensity of intestinal inflammation. The effects of m^6^A modification therefore have to be read in relation to the cell type and tissue environment in which they occur. It can shape T‐cell and B‐cell differentiation and effector function, while also influencing the maintenance of intestinal epithelial and stem‐cell homeostasis. This context dependence may help explain the apparently opposite outcomes: METTL3‐mediated m^6^A supports pathogenic Th17 responses in EAE, whereas B‐cell METTL3 appears protective in EAE; in SLE and UC, reduced METTL3 is linked to loss of immune or epithelial homeostasis, while in CeD increased m^6^A‐dependent IRF7 or XPO1 signaling may contribute to intestinal inflammation.

### 4.6 RBPs

In ADs, RBPs may contribute to heterogeneity by altering the posttranscriptional fate of inflammatory RNAs. The studies summarized here mainly support two mechanisms: RBP‐dependent mRNA decay or derepression in lymphocytes, and RBP‐dependent translational amplification in target tissues.

Roquin‐1 and Regnase‐1 illustrate how defective mRNA repression can reshape systemic immune responses. Behrens et al. [96] showed that Roquin‐1 cooperates with Regnase‐1 to posttranscriptionally regulate shared target mRNAs. Disruption of this interaction derepressed transcripts encoding costimulatory molecules such as inducible T‐cell costimulator (ICOS), promoted aberrant T‐cell activation, T follicular helper (Tfh) cell expansion, germinal center B‐cell responses, and autoantibody production, and ultimately resulted in systemic humoral autoimmunity. Bhat et al. [97] further showed that Regnase‐1‐mediated mRNA control is essential in B cells. B cell‐specific deletion of Regnase‐1 derepressed a transcriptional program related to B‐cell activation, survival, and differentiation, thereby triggering systemic autoimmunity and early morbidity. These findings indicate that failure of RBP‐mediated mRNA turnover can generate distinct lymphocyte‐specific autoimmune programs.

RBPs can also shape organ‐specific injury through translational control. Li et al. [98] found that Arid5a was upregulated in human and mouse autoantibody‐mediated glomerulonephritis (AGN) and was elevated in renal tubular epithelial cells during AGN. Mechanistically, Arid5a promoted IL‐17‐dependent renal inflammation partly by interacting with IL‐17 target mRNAs, including CEBPB and CEBPD, and with 18S ribosomal RNA (18S rRNA), thereby supporting CEBPB and CEBPD mRNA translation and downstream CCAAT/enhancer‐binding protein (C/EBP)‐dependent inflammatory programs in renal tubular epithelial cells. Loss of Arid5a attenuated myeloid cell infiltration and impaired IL‐17‐dependent cytokine and transcription factor programs, rendering mice resistant to AGN pathology. Thus, RBP dysregulation can shape autoimmune heterogeneity through different posttranscriptional mechanisms: mRNA derepression in T and B lymphocytes promotes systemic humoral autoimmunity, whereas translational amplification in renal tissue promotes organ‐specific inflammatory injury.

### 4.7 RNA Editing

RNA editing modifies sequence information after transcription, at the RNA rather than DNA level. A major form of this process is adenosine‐to‐inosine (A‐to‐I) editing, which is catalyzed by the adenosine deaminase acting on RNA (ADAR) family. A‐to‐I editing affects RNA processing and secondary structure. It also lowers the likelihood that endogenous double‐stranded RNA (dsRNA) will activate RNA sensors, which helps maintain innate immune homeostasis.

Recent studies suggest that the inflammatory consequences of RNA‐editing defects differ by tissue. Xu et al. [99] found that intestinal ADAR1 deficiency impaired A‐to‐I RNA editing and led to accumulation of endogenous dsRNA and endogenous retroviruses (ERVs). These RNAs activated melanoma differentiation‐associated protein 5 (MDA5)‐mediated sensing and Janus kinase (JAK)–signal transducer and activator of transcription (STAT) signaling, followed by spontaneous ileitis and colitis. These findings link defective RNA editing to immune‐mediated intestinal inflammation through endogenous RNA accumulation and abnormal innate immune sensing in the gut. In a joint‐inflammation setting, Fang et al. [100] focused on ADAR1, an RNA‐editing enzyme that catalyzes A‐to‐I editing in dsRNAs and can influence circRNA biogenesis. They observed low ADAR1 expression in RA‐FLSs. ADAR1 overexpression alleviated arthritis severity, inflammatory responses, and cartilage degradation in severe combined immunodeficiency (SCID) mice implanted with human cartilage and RA‐FLSs (SCID‐HuRAg), and this protective effect was associated with reduced circFTO and hnRNPA2B1 expression and increased miR‐548a‐3p expression in the implanted tissues. Read alongside the intestinal data, these results suggest that ADAR1 dysregulation can produce different tissue‐specific inflammatory manifestations. Its consequences differ across target tissues and may therefore contribute to AD heterogeneity.

### 4.8 Summary

Posttranscriptional regulation therefore adds a molecular layer beyond genomic and transcriptional variation, helping explain why autoimmune heterogeneity can emerge after transcription. These mechanisms act through RNA isoforms, regulatory RNA networks, RNA modification states, RNA stability, translation, and editing‐dependent immune sensing. Their downstream effects include immune‐cell differentiation, inflammatory amplification, stromal activation, epithelial barrier integrity, and target‐organ injury, with the direction of effect depending on context. Their effects vary with immune‐cell subset, tissue microenvironment, and disease setting. This variation helps explain why related autoimmune pathways may still produce different levels of inflammation, patterns of organ involvement, tissue damage, and clinical phenotype.

Posttranscriptional mechanisms also have important translational implications. Such RNA‐level alterations may generate molecular signatures. Because some RNA‐level signals can be measured in blood, extracellular vesicles (EVs), or disease‐relevant tissues, they may help support disease‐activity assessment, organ‐involvement evaluation, and estimation of disease trajectory or tissue‐damage risk after further validation. At the same time, RNA–control pathways may inform therapeutic stratification by identifying inflammatory programs driven by RNA processing, RNA stability, RNA modification, or RNA‐sensing defects.

## 5 Functional Reprogramming in Shaping Heterogeneity in ADs

**FIGURE 4:**
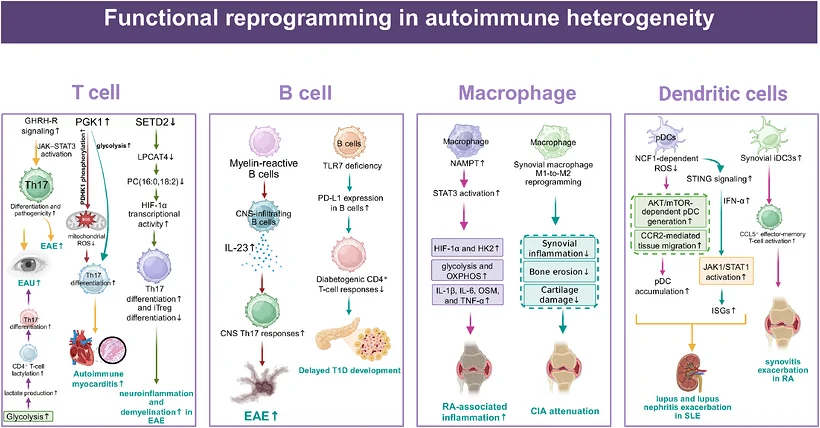
Functional reprogramming as the cellular output of upstream molecular dysregulation. Chronic inflammatory, metabolic, epigenetic, and tissue‐derived cues reshape T cells, B cells, macrophages, and dendritic cells into pathogenic, regulatory, or tissue‐adapted states, thereby shaping disease activity and organ‐specific injury. iDC3s, inflammatory type 3 dendritic cells; EAE, experimental autoimmune encephalomyelitis; GHRH‐R, growth hormone‐releasing hormone receptor; IL‐1β, interleukin‐1β; IL‐6, interleukin‐6; IL‐23, interleukin‐23; JAK–STAT3, Janus kinase–signal transducer and activator of transcription 3; NAMPT, nicotinamide phosphoribosyltransferase; NCF1, neutrophil cytosolic factor 1; pDC, plasmacytoid dendritic cell; pDCs, plasmacytoid dendritic cells; PGK1, phosphoglycerate kinase 1; RA, rheumatoid arthritis; ROS, reactive oxygen species; SETD2, SET domain‐containing 2 histone lysine methyltransferase; SLE, systemic lupus erythematosus; Th17, T helper 17 cell; iTreg, induced regulatory T cell; AKT, protein kinase B; B cell, B lymphocyte; CCL5⁺, C‐C motif chemokine ligand 5‐positive; CCR2, C‐C motif chemokine receptor 2; CD4⁺, cluster of differentiation 4‐positive; CIA, collagen‐induced arthritis; CNS, central nervous system; EAU, experimental autoimmune uveitis; HIF‐1α, hypoxia‐inducible factor 1α; HK2, hexokinase 2; IFN‐α, interferon‐α; ISGs, interferon‐stimulated genes; JAK1, Janus kinase 1; LPCAT4, lysophosphatidylcholine acyltransferase 4; M1, classically activated macrophage phenotype; M2, alternatively activated macrophage phenotype; mTOR, mechanistic target of rapamycin; OSM, oncostatin M; OXPHOS, oxidative phosphorylation; PC(16:0,18:2), phosphatidylcholine containing 16:0 and 18:2 fatty acyl chains; PDHK1, pyruvate dehydrogenase kinase 1; PD‐L1, programmed death‐ligand 1; STAT1, signal transducer and activator of transcription 1; STAT3, signal transducer and activator of transcription 3; STING, stimulator of interferon genes; T cell, T lymphocyte; T1D, type 1 diabetes; TLR7, Toll‐like receptor 7; TNF‐α, tumor necrosis factor‐α. Created in BioRender. L, K. (2026) https://BioRender.com/0kyx1kx.

Functional reprogramming is used here to describe disease‐ and tissue‐context‐dependent redirection of differentiated immune cells toward pathogenic, regulatory, or nonpathogenic states in response to persistent inflammatory, metabolic, epigenetic, or intercellular cues. These reprogrammed states can persist beyond transient signaling events and are characterized by altered activation programs, cytokine output, metabolic behavior, tissue localization, and interactions with other immune cells [56, 101, 102, 103, 104]. In ADs, this process is better viewed as more than a fluctuation in one signaling pathway. Instead, it can be read as the phenotypic convergence of upstream molecular perturbations, especially immunometabolic remodeling and epigenetic stabilization, together with additional regulatory inputs that reinforce particular cell states [19, 56, 103, 105, 106, 107]. Once established, this reprogramming can redirect immune‐cell differentiation trajectories, tissue localization, cytokine production, and intercellular communication. These changes may then affect inflammatory amplification, organ involvement, and disease evolution [101, 102, 104]. The direction and stability of reprogramming vary across immune‐cell lineages and tissue niches. As a result, distinct cellular states may emerge even within a single disease entity and add to clinical and immunological heterogeneity [104]. This section therefore uses four immune‐cell compartments, T cells, B cells, macrophages, and dendritic cells (DCs), to examine how upstream molecular dysregulation is translated into cell‐state outputs and ultimately into divergent disease phenotypes (Figure 4).

### 5.1 T‐Cell Reprogramming and Divergence Between Pathogenicity and Immune Restraint

T‐cell reprogramming offers one of the most visible examples of autoimmune heterogeneity. In different tissues, these T‐cell programs can tilt inflammation toward restraint, persistence, or amplification.

The Th17–Treg relationship is one of the better‐characterized examples of this balance. In collagen‐induced arthritis, Treg cells coexpressing retinoic acid receptor‐related orphan receptor gamma t (RORγt) and FOXP3, termed T regulatory 17 (Tr17) cells, accumulate in inflamed joints, express suppressive molecules including IL‐10, and suppress conventional T‐cell proliferation, supporting the idea that this hybrid state can act as a local restraint on autoimmune joint inflammation rather than merely as a pathogenic deviation [108]. By contrast, growth hormone‐releasing hormone receptor (GHRH‐R) expression is induced during Th17 differentiation, and GHRH‐R signaling reinforces pathogenic Th17 programming through JAK/STAT3 signaling; GHRH‐R deficiency alleviates both EAE and autoimmune uveitis, whereas pharmacologic inhibition alleviates autoimmune uveitis [109]. Treg cells specific for the Smith (Sm) autoantigen suppress Sm‐specific proinflammatory responses in vitro and disease progression in a humanized model of lupus nephritis [110], whereas loss of RORγt serine‐182 phosphorylation increases excessive Th17 activation and reduces IL‐10‐associated regulatory output [107]. These examples make an important point: T‐cell plasticity is not unidirectional. Disease severity and organ‐selective injury may depend, at least in part, on how pathogenic and regulatory reprogramming are balanced.

Metabolic and epigenetic changes can also work together to stabilize T‐cell functional states. In experimental autoimmune uveitis, enhanced glycolysis increases lactate accumulation, and lactate‐derived lactylation promotes Th17 differentiation and worsens ocular inflammation; specifically, Ikzf1 Lys164 lactylation promotes Th17 differentiation by altering Ikzf1 binding to Th17‐related gene loci [105]. In experimental autoimmune myocarditis, pharmacologic phosphoglycerate kinase 1 (PGK1) inhibition reduces pathogenic Th17 and Th1 responses, shifts the balance toward Treg cells and reduces cardiac injury [111]. Disruption of the nonoxidative PPP in Treg cells weakens suppressive function through linked metabolic, epigenetic, and transcriptional alterations [56]. Epigenetic regulation adds another layer to these differences. SET domain containing 2 (SETD2) restrains Th17 differentiation and promotes induced Treg formation through lysophosphatidylcholine acyltransferase 4 (Lpcat4)‐dependent phosphatidylcholine remodeling and suppression of HIF‐1α activity [103]. T‐cell reprogramming therefore links phenotypic plasticity with metabolic and epigenetic stabilization. This combination helps explain both disease‐specific and organ‐specific heterogeneity.

### 5.2 B‐Cell Reprogramming and Tissue‐Dependent Humoral Pathogenicity

In ADs, B‐cell pathogenicity extends beyond autoantibody production [112]. B cells also present antigens, secrete cytokines, and engage in reciprocal functional interactions with T cells [102, 112]. B‐cell reprogramming depends on tissue context, developmental stage, and inflammatory cues [113, 114]. This makes B cells a plausible source of heterogeneity both between diseases and within the same disease. B‐cell receptor (BCR) signaling further links these cues to B‐cell heterogeneity by regulating activation thresholds, survival, differentiation, tolerance checkpoints, and autoreactive humoral responses across autoimmune rheumatic diseases [112].

Tissue microenvironments may impose B‐cell states that are specific to a given disease setting. In nonobese diabetic (NOD) mice, islet B cells show transcriptional programs that differ from those of pancreatic lymph node B cells. These programs include coordinated upregulation of antibody‐secreting cell differentiation genes, interferon pathways, proinflammatory cytokines, and Toll‐like receptor signaling, suggesting that the local islet niche may reprogram B cells toward a more pathogenic state [113]. Consistent with this, Toll‐like receptor 7 (TLR7) deficiency in female NOD mice delays T1D onset and reduces disease incidence while reshaping B‐cell differentiation, antigen‐presenting capacity, and interactions with T cells. This supports the idea that TLR7‐dependent innate sensing can influence whether B cells adopt proinflammatory and antigen‐presenting programs [115]. Bone marrow single‐cell transcriptomics and BCR analyses reveal heterogeneous developmental programs in SLE, including a subgroup marked by defective early B‐cell development, stronger IFN‐I and metabolic activation, and more abnormal BCR repertoires. The partial reversibility of these abnormalities observed in one patient during remission suggests that B‐cell developmental reprogramming may be dynamic and clinically relevant [114].

B cells also contribute to heterogeneity by altering the behavior of other immune compartments. In a CNS autoimmunity model relevant to MS, myelin‐reactive B cells exacerbate CD4^+^ T‐cell‐driven disease in an IL‐23‐dependent manner [102]. They also promote meningeal lymphoid aggregation and Th17 accumulation [102]. Autoreactive B cells recognizing posttranslationally modified antigens are enriched among activated C‐X‐C motif chemokine receptor 3 (CXCR3)‐positive memory B cells and plasmablasts with enhanced activation and migration‐associated features in RA [116]. This pattern suggests that persistent activation of the autoreactive B‐cell compartment may contribute to disease chronicity in RA [116]. B‐cell reprogramming therefore has two sides: intrinsic changes in differentiation and the ability of B cells to reshape T‐cell responses and local immune niches. Through both routes, it may add to pathological heterogeneity.

### 5.3 Macrophage Reprogramming and Inflammatory Microenvironment Diversification

Macrophages show marked plasticity during autoimmune inflammation [117]. Macrophage reprogramming is often described in terms of classically activated M1‐like and alternatively activated M2‐like states, but disease‐associated macrophage states rarely fit a simple binary model [117]. Instead, macrophages often acquire hybrid or persistently inflammatory programs in which inflammatory output is coupled to altered metabolism or tissue remodeling [27, 117].

In a collagen‐induced arthritis model, macrophage polarization is associated with disease severity and tissue destruction [101]. When synovial macrophages move away from a proinflammatory state and toward anti‐inflammatory programs, joint inflammation and tissue injury are alleviated [101, 118]. This supports the view that macrophage polarization may influence experimental arthritis severity [101, 118]. RA macrophage abnormalities, however, cannot be reduced to simple M1 skewing. Patient‐derived monocytes and monocyte‐derived macrophages show exaggerated inflammatory responses, and the macrophages additionally show increased glycolysis and OXPHOS, abnormal mitochondrial morphology, and impaired phagocytic function. Transcriptomic analyses link this highly inflammatory and highly metabolic phenotype to NAMPT and STAT3 signaling, and inhibition of either pathway reduces proinflammatory gene expression, whereas NAMPT inhibition, but not STAT3 inhibition, helps restore macrophage phagocytic function [27]. Macrophage reprogramming in RA may therefore be better framed as a sustained inflammatory‐metabolic state [27].

Macrophage reprogramming in SLE is also linked to metabolic deviation, but the pathological output differs from that seen in RA. In monocytes from patients with SLE, ubiquitin‐specific peptidase 18 (USP18) mRNA expression tended to correlate negatively with the expression of metabolism‐related genes [119]. In mouse bone marrow‐derived macrophages, USP18 deficiency enhanced M1‐like polarization induced by lipopolysaccharide plus IFN‐γ, together with glycolysis, mitochondrial respiration, mitochondrial DNA release, and ROS production; conversely, USP18 overexpression suppressed the M1‐like program and favored IL‐4‐driven polarization [119]. These studies place macrophage polarization imbalance in SLE within an immunometabolic framework and suggest that it may add to systemic inflammatory amplification. Macrophage reprogramming therefore appears to be both tissue dependent and disease dependent. This context dependence may help drive clinical heterogeneity.

### 5.4 DC Reprogramming and the Balance Between Immunogenicity and Tolerance

DCs sit at the interface between innate and adaptive immunity. Their reprogramming can shift immune responses toward immunogenic, tolerogenic, or inflammatory states [104]. In ADs such as RA, DC reprogramming can involve changes in activation state as well as redistribution of distinct DC subsets across tissue niches [104].

Plasmacytoid DCs (pDCs) illustrate a disease‐specific reprogramming state dominated by high interferon activity in SLE. In mouse lupus models, reduced neutrophil cytosolic factor 1 (NCF1)‐dependent ROS production promotes pDC accumulation in multiple organs and is accompanied by elevated IFN‐α levels and IFN‐I‐stimulated gene expression. These changes worsen lupus phenotypes [106]. Moreover, Smith ribonucleoprotein‐containing immune complexes comprising immunoglobulin A1 (IgA1) and immunoglobulin G (IgG) autoantibodies show greater binding to and internalization by pDCs than IgA1‐depleted complexes in vitro [120]. Coengagement of the IgA‐specific Fc receptor (FcαR; CD89) and Fc gamma receptor IIa (FcγRIIa) markedly amplifies pDC IFN‐α responses in vitro [120]. These findings suggest a feedback route from humoral abnormalities to pDC activation and interferon output, with the potential to reinforce interferon amplification [120]. Autoantibody isotypes and pDC FcαR expression vary across patients [120]. For this reason, the extent of pDC reprogramming may help explain differences in interferon activity, disease activity, and organ involvement.

In RA, DC reprogramming is reflected in both niche‐specific redistribution of DC subsets and their maturation into distinct functional states. Single‐cell, spatial transcriptomic, and cell‐trajectory analyses suggest that, in healthy synovium, infiltrating dendritic cell 2 (DC2) populations can mature into AXL receptor tyrosine kinase (AXL)‐positive DC2 states with tolerance‐associated features beneath the protective lining layer [104]. In active RA synovium, infiltrating inflammatory dendritic cell 3 (iDC3) mature into activated tissue phenotypes that stimulate effector memory T cells, potentially promoting synovitis [104]. In RA sublining lymphoid niches, infiltrating DC2s can mature through a highly activated C‐C motif chemokine receptor 7 (CCR7)‐expressing intermediate state into immunogenic DC2 states that interact with naive T cells, potentially supporting local T‐cell priming and effector expansion [104]. Importantly, sustained clinical and imaging remission is accompanied by regression of these pathogenic niches, but the tolerogenic AXL‐positive DC2 compartment is not fully restored [104]. A proinflammatory gene signature can already be detected in circulating iDC3 precursors before flare onset [104]. DC reprogramming therefore reflects functional and spatial reorganization of the local immune architecture in RA, not simply a shift in cell abundance. Across diseases, DCs are linked to heterogeneity through interferon output, T‐cell priming, and reconstruction of tissue immune niches.

### 5.5 Summary

Functional reprogramming therefore provides a way to connect upstream molecular dysregulation with divergent autoimmune phenotypes. Across immune‐cell compartments, reprogrammed cellular states link metabolic and epigenetic alterations, tissue‐specific cues, and intercellular communication to differences in inflammatory intensity, organ involvement, relapse propensity, and disease evolution. This framework is useful because it places cellular plasticity within tissue context and cross‐cellular regulation. From that perspective, distinct pathogenic and regulatory immune programs can be understood as sources of heterogeneous clinical trajectories in ADs. T‐cell, B‐cell, macrophage, and DC functional programs may help identify dominant pathogenic compartments, clarify tissue‐specific immune pressure, and provide a mechanistic basis for subsequent biomarker interpretation and therapeutic stratification.

## 6 Biomarkers Associated With Multidimensional Regulatory Networks in ADs

Autoimmune pathogenesis involves disturbances across several connected regulatory layers. Within these layers, molecular abnormalities are relevant not only to disease onset and progression but also to biomarker discovery for diagnosis, monitoring, prognosis, and treatment stratification. Conventional serological biomarkers remain useful in clinical practice, but studies in RA and SLE show that commonly used markers still have limitations in sensitivity or specificity for early and accurate diagnosis [121, 122]. These limitations motivate the development of complementary biomarkers for disease classification, activity monitoring, and treatment–response stratification. Recent mechanistic and biomarker studies describe multidimensional alterations in ADs, including metabolic abnormalities, hypo‐ or hypermethylation at specific DNA loci, dysregulated ncRNAs, altered m^6^A‐related molecules, and immune‐cell activation, exhaustion, or altered function [19, 123, 124, 125, 126].

For a biomarker to be clinically useful in ADs, it should ideally link a mechanistic signal to a decision that can be made in practice. Practically, these purposes are not the same: a biomarker may help with diagnosis or differential diagnosis, reflect disease activity or organ involvement, estimate disease trajectory, or identify patients more likely to respond to a given therapeutic strategy. Current biomarker signals are increasingly derived from multidimensional regulatory abnormalities, including metabolic intermediates, DNA methylation signatures, RNA‐based markers, m^6^A‐related molecules, and cell‐state indicators. However, these signals differ substantially in translational maturity: some are supported by multicohort or longitudinal validation and may be considered validated candidates with potential clinical application, whereas many others remain exploratory candidates based on differential expression, cross‐sectional association, internal validation, or mechanistic relevance.

In this review, biomarker evidence is interpreted according to translational maturity. We distinguish clinically established biomarkers, validated biomarker candidates, exploratory candidate biomarkers, and mechanistic or pharmacodynamic indicators. Clinically established biomarkers refer to assays that are already used in routine clinical decision‐making or are incorporated into accepted diagnostic or monitoring frameworks. Validated biomarker candidates have been tested in independent cohorts, larger multicohort datasets, or longitudinal settings, but still require standardization and prospective clinical evaluation before routine use. Exploratory candidate biomarkers are supported mainly by differential expression, cross‐sectional association, single‐cohort diagnostic performance, or internal validation. Mechanistic or pharmacodynamic indicators are useful for understanding disease biology or therapeutic effects but should not be presented as diagnostic, prognostic, or stratification biomarkers unless further validated in clinically defined cohorts. This distinction is important because molecular dysregulation or disease association does not by itself establish clinical applicability. Table 1 summarizes representative biomarker signals according to proposed clinical utility and current evidence level.

**TABLE 1:** Representative biomarker signals in autoimmune diseases stratified by proposed clinical utility and current evidence level.

Clinical utility	Biomarker	Disease	Expression/change	Biomarker evidence	Key clinical associations	Evidence level	References
Diagnosis and differential diagnosis	Trp–Kyn metabolite panel: Trp, XA, CA, and KynA	RA	↓	RA–HC model shows high discriminatory performance; reduced Trp and XA are linked to Th17/Treg imbalance, and reduced KynA is linked to Tfh/Tfr imbalance.	Metabolic signature of immune‐cell imbalance	Exploratory candidate	[127]
	IFI44L promoter methylation	SLE, cSLE	Hypomethylation	Site1 cutoff shows high sensitivity and specificity and distinguishes SLE from RA and pSS; diagnostic performance is also validated in cSLE.	IFN‐associated epigenetic dysregulation	Validated candidate with potential clinical application	[122, 128]
	CDKN2A promoter methylation	SLE, RA	Hypomethylation	PBMC methylation levels show diagnostic performance for distinguishing patients from HC.	Blood‐based epigenetic abnormality shared by SLE and RA	Exploratory candidate	[129]
	NEAT1_2	cSLE	↑	ROC analysis shows diagnostic relevance.	lncRNA dysregulation in childhood‐onset autoimmunity	Exploratory candidate	[130]
	TGFB2‐OT1 (NR_125715.1)	RA	↑	PBMC expression shows diagnostic discrimination and correlates with RA‐related serological markers.	PBMC lncRNA dysregulation associated with RA‐related autoantibody status	Exploratory candidate	[131]
	RP11‐273G15.2	SLE	↑ in B cells	B‐cell expression discriminates SLE from HC and, in most B‐cell subsets except naïve B cells, from RA and SSc.	Pathogenic B‐cell activation and Type I IFN‐pathway involvement	Validated exploratory candidate	[132]
	ALKBH5, FTO, and YTHDF2	RA	↓	ALKBH5, FTO, and YTHDF2 are decreased in RA; FTO is associated with DAS28 and complement‐related parameters, whereas YTHDF2 is associated with inflammatory‐cell indices and inflammatory cytokine‐related markers.	m^6^A‐related inflammatory regulatory abnormality	Exploratory candidate	[133, 134]
Disease activity and organ involvement assessment	SII and serum Klotho	RA	Higher SII is associated with lower serum Klotho	SII is negatively correlated with serum Klotho.	Systemic inflammatory burden	Exploratory systemic‐burden marker	[135]
	Trp, KYN, KynA, 3‐HAA, 3‐HK, QA, and KYN/Trp ratio	SLE	Trp↓; KYN, KynA, 3‐HAA, 3‐HK, QA↑; KYN/Trp ratio↑	Serum KP profile differs from HC; KYN/Trp ratio is also correlated with disease activity.	Systemic KP activation and immunometabolic imbalance	Exploratory candidate	[136]
	ACOD1–itaconate axis	RA	ACOD1 mRNA↓ in active disease	PBMC itaconate is inversely correlated with disease activity, and itaconate inhibits FLS proliferation and migration.	Active inflammation and FLS proliferative/migratory behavior	Candidate activity‐associated signal plus preclinical mechanistic indicator	[51, 137]
	IFI44L promoter methylation	SLE	Hypomethylation; partial recovery in remission; lower with renal involvement	Methylation levels differ between active and remission states and by renal damage status.	IFN‐linked activity state and renal involvement	Exploratory activity‐ and organ‐involvement‐associated candidate	[122]
	CDKN2A promoter methylation	SLE	Hypomethylation	Methylation level is associated with anti‐dsDNA, C3, and C4.	Anti‐dsDNA‐ and complement‐associated disease activity	Exploratory candidate	[129]
	Renal damage‐associated DNAm loci in CD4^+^ T cells	SLE, LN	Renal damage‐associated abnormal DNAm pattern	Validated DMPs discriminate LN from nonrenal SLE and HC.	Renal damage‐associated T‐cell epigenetic state	Exploratory organ‐involvement candidate	[60]
	MMP9 promoter methylation	SLE, especially renal involvement	Hypomethylation	Methylation level differs by renal involvement and correlates with creatinine, C3, and C4.	Renal injury and complement consumption	Exploratory candidate	[138]
	RUNX3 promoter methylation	SLE, especially renal involvement	Hypermethylation	Methylation level differs by renal involvement and correlates with creatinine.	Renal injury‐associated epigenetic alteration	Exploratory candidate	[139]
	miR‐155	SLE, LN	↑	miR‐155 correlates with SLEDAI and distinguishes active nephritis from inactive or absent nephritis.	Active lupus nephritis and systemic disease activity	Exploratory candidate	[125]
	RP11‐273G15.2	SLE	↑	Expression correlates with IFN score, SLEDAI, and ISG expression.	IFN‐driven pathogenic B‐cell activation	Validated exploratory candidate	[132]
	ALKBH5	SLE	↓	Expression is associated with disease‐activity‐related laboratory parameters, including N%, NLR, C3, and fever.	Active inflammatory and complement‐consumption state	Exploratory candidate	[153]
Prognosis and relapse assessment	SII	RA	↑	Higher SII is associated with increased all‐cause and CV mortality.	Mortality‐associated systemic inflammation	Potential prognostic candidate	[140]
	ACOD1–itaconate axis	RA	ACOD1 deficiency or reduced ACOD1–itaconate‐axis activity	The axis suppresses Hif1α‐mediated glycolysis and osteoclast differentiation; deficiency aggravates bone loss.	Osteoclastogenesis and bone‐destructive phenotype	Preclinical/mechanistic candidate	[137]
	Four‐metabolite relapse panel: lysine, asparagine, isoleucine, and leucine	RRMS	During relapse: lysine↑, asparagine↑; isoleucine↓, leucine↓	The four‐metabolite panel distinguishes relapse from remission in group‐level and paired‐sample analyses.	Relapse‐associated metabolic state	Validated within‐cohort candidate	[141]
	Longitudinal DNAm changes and methylation‐based subtypes	SLE	Dynamic change	Longitudinal DNAm changes are associated with remission status and clinical/biological subtypes.	Flare–remission‐associated epigenetic heterogeneity	Longitudinal exploratory candidate	[142]
	EV‐miR‐557	AIH	↑	EV‐miR‐557 >7.69 copies/µL is associated with higher 7‐year relapse incidence.	Relapse‐prone inflammatory state	Candidate diagnostic and relapse‐risk biomarker	[143]
	Activated Tph cells, activated CD8^+^ T cells, and BAFF	AIH	Activated Tph↑ and activated CD8^+^ T cells↑ in active AIH; activated Tph↑/activated CD8^+^ T cells↑ and BAFF↑ in relapse‐prone patients before immunosuppression withdrawal	Higher activated Tph cells, activated CD8^+^ T cells, and BAFF before immunosuppression withdrawal are associated with relapse.	Relapse‐prone immune activation state	Candidate relapse‐risk markers	[144]
	MMP9	SLE	m^6^A‐related MMP9 dysregulation	MMP9 correlates with m^6^A modification and is causally linked to ischemic stroke risk in MR analysis.	m^6^A‐related complication‐risk signal	Hypothesis‐generating computational/MR signal	[126]
Treatment response prediction and patient stratification	Plasma proteomic response model	RA	Treatment‐responsive protein signature	Distinct plasma protein signatures predict responses to different csDMARD combinations.	Therapy‐associated inflammatory and metabolic pathway remodeling	Validated predictive model with potential clinical application	[145]
	Baseline SII	RA	Lower baseline level is favorable	Lower pretreatment SII is observed in responders and is associated with TNF‐α inhibitor efficacy.	Inflammation‐linked treatment responsiveness	Retrospective exploratory candidate	[146]
	Plasma itaconate	Early RA	↑ after csDMARD‐associated improvement	Change in itaconate correlates with DAS44 and CRP improvement.	Anti‐inflammatory metabolic response	Candidate pharmacodynamic marker	[147]
	miR‐155 dynamics under TNF inhibitors	RA	Dynamic change	Different TNF inhibitors differentially modulate miR‐155 and M2‐like macrophage polarization.	Drug‐specific macrophage polarization response	Candidate pharmacodynamic indicator	[148]
	m^6^A score and m^6^A modification pattern	RA	Lower m^6^A score	Lower m^6^A score is associated with greater infliximab benefit.	m^6^A‐defined infliximab‐responsive immune phenotype	Computational candidate stratification model	[149]
	B‐cell depletion and reconstitution after anti‐CD19 CAR‐T	Refractory SLE	Deep B‐cell depletion; reconstituted B cells mainly show IgM and IgD heavy chains	CAR‐T therapy induces B‐cell depletion and clinical remission, followed by nonclass‐switched B‐cell reconstitution.	Possible immune reset with non‐class‐switched B‐cell recovery	Pharmacodynamic response indicator	[8]

*Abbreviations*: 3‐HAA, 3‐hydroxyanthranilic acid; 3‐HK, 3‐hydroxykynurenine; ACOD1, aconitate decarboxylase 1; AIH, autoimmune hepatitis; ALKBH5, AlkB homolog 5; anti‐dsDNA, anti‐double‐stranded DNA antibody; BAFF, B‐cell activating factor; C3, complement component 3; C4, complement component 4; CA, cinnabarinic acid; CAR‐T, chimeric antigen receptor T‐cell therapy; CD19, cluster of differentiation 19; CD4, cluster of differentiation 4; CD8, cluster of differentiation 8; CDKN2A, cyclin‐dependent kinase inhibitor 2A; CRP, C‐reactive protein; csDMARD, conventional synthetic disease‐modifying antirheumatic drug; cSLE, childhood‐onset systemic lupus erythematosus; CV, cardiovascular; DAS28, Disease Activity Score based on 28 joints; DAS44, Disease Activity Score based on 44 joints; DMP/DMPs, differentially methylated position(s); DNAm, DNA methylation; EV, extracellular vesicle; EV‐miR‐557, extracellular vesicle‐associated microRNA‐557; FLS, fibroblast‐like synoviocyte; FTO, fat mass and obesity‐associated protein; HC, healthy control; HIF‐1α/Hif1α, hypoxia‐inducible factor 1α; IFI44L, interferon‐induced protein 44‐like; IFN, interferon; IgD, immunoglobulin D; IgM, immunoglobulin M; ISG, interferon‐stimulated gene; KP, kynurenine pathway; KYN/Kyn, kynurenine; KYN/Trp, kynurenine‐to‐tryptophan ratio; KynA, kynurenic acid; LN, lupus nephritis; lncRNA, long noncoding RNA; M2‐like, alternatively activated macrophage‐like phenotype; m^6^A, N^6^‐methyladenosine; miR, microRNA; MMP9, matrix metalloproteinase 9; MR, Mendelian randomization; mRNA, messenger RNA; N%, neutrophil percentage; NEAT1_2, nuclear paraspeckle assembly transcript 1 isoform 2; NLR, neutrophil‐to‐lymphocyte ratio; PBMC, peripheral blood mononuclear cells; pSS, primary Sjögren's syndrome; QA, quinolinic acid; RA, rheumatoid arthritis; ROC, receiver operating characteristic; RP11‐273G15.2, long noncoding RNA RP11‐273G15.2; RRMS, relapsing‐remitting multiple sclerosis; RUNX3, runt‐related transcription factor 3; SII, systemic immune‐inflammation index; SLE, systemic lupus erythematosus; SLEDAI, Systemic Lupus Erythematosus Disease Activity Index; SSc, systemic sclerosis; Tfh, T follicular helper cell; Tfr, T follicular regulatory cell; TGFB2‐OT1, transforming growth factor beta 2 overlapping transcript 1; Th17, T helper 17 cell; TNF, tumor necrosis factor; TNF‐α, tumor necrosis factor‐α; Tph, T peripheral helper cell; Treg, regulatory T cell; Trp, tryptophan; XA, xanthurenic acid; YTHDF2, YTH N^6^‐methyladenosine RNA‐binding protein F2.

### 6.1 Biomarkers for Diagnosis and Differential Diagnosis

Early diagnosis and accurate differential diagnosis of ADs are crucial for improving patient outcomes. Toward this goal, studies in the fields of immunometabolism, epigenetics, posttranscriptional regulation, and functional reprogramming have identified a series of promising candidate biomarkers.

In the field of immunometabolism, patients with SLE exhibit increased serum kynurenine (KYN), kynurenic acid (KynA), 3‐hydroxyanthranilic acid (3‐HAA), 3‐hydroxykynurenine (3‐HK), and quinolinic acid (QA) levels [136]. In a small cohort of patients with new‐onset, untreated, seropositive RA, the serum tryptophan–KYN pathway was downregulated, and seven differential serum tryptophan metabolites showed decreased levels; among these, tryptophan, xanthurenic acid (XA), cinnabarinic acid (CA), and KynA were selected as the top four features for the classification model [127]. In the same small cohort, patients with RA exhibit an increased proportion of Tfh cells, a decreased proportion of T follicular regulatory (Tfr) cells, and thus an elevated Tfh/Tfr ratio, while reduced tryptophan and XA are associated with a greater degree of Th17/Treg imbalance and reduced KynA was associated with a greater degree of Tfh/Tfr imbalance; a machine‐learning classification model based on tryptophan, XA, CA, and KynA showed high discrimination between patients with RA and healthy controls after internal validation [127]. However, because external validation and disease‐control comparisons are still lacking, this panel should be regarded as an exploratory candidate rather than a clinically applicable diagnostic biomarker [127].

Epigenetic biomarkers provide another major source of diagnostic information. In the field of epigenetics, patients with SLE show widespread DNA methylation abnormalities, including altered methylation in CD4^+^ T cells and B cells and hypomethylation at interferon‐regulated loci [19, 122]. Accordingly, DNA methylation abnormalities represent a promising class of molecular biomarker candidates for SLE diagnosis [19, 122]. Two CpG sites within the promoter of interferon‐induced protein 44‐like (IFI44L), Site1 and Site2, are hypomethylated in SLE [122]. IFI44L promoter hypomethylation at Site1 and Site2 has been evaluated in large adult SLE cohorts and independent validation cohorts, making it one of the better‐supported validated diagnostic biomarker candidates for SLE [122]. Nevertheless, its routine clinical use would still require standardized assay implementation and prospective evaluation alongside established serological tests. Zhao et al. found that Site1 methylation can effectively distinguish SLE from RA and primary Sjögren's syndrome (pSS) in an adult cohort [122]. IFI44L methylation is likewise significantly reduced in pediatric patients and demonstrates good diagnostic performance in a childhood‐onset SLE clinical validation cohort [128]. In addition, methylation of the cyclin‐dependent kinase inhibitor 2A (CDKN2A) promoter in PBMCs is markedly reduced in both SLE and RA compared with healthy controls, suggesting that it may serve as an exploratory auxiliary blood‐based diagnostic candidate for both diseases [129]. In SLE, its association with anti‐double‐stranded DNA (anti‐dsDNA) and complement levels suggests activity‐related relevance, whereas the reported cohort did not support a clear association with renal involvement or creatinine [129].

Posttranscriptional regulatory networks also provide RNA‐based biomarker candidates. Decreased miR‐98‐5p expression is accompanied by increased IFI44L expression in PBMCs and CD14^+^ monocytes from patients with SLE, and miR‐98‐5p directly targets IFI44L, thereby inhibiting the differentiation of monocytes into monocyte‐derived DCs and IFI44L‐mediated interferon responses in vitro [150]. Therefore, the miR‐98‐5p/IFI44L axis may provide mechanistic support for IFI44L‐related biomarker development [150]. A review of lncRNA expression profiles in SLE and RA has summarized dysregulated lncRNAs in PBMCs, serum or plasma, and exosomes, supporting further investigation of lncRNA signatures as biomarker candidates in SLE and RA [151]. In serum‐derived exosomes from patients with RA, the lncRNA nuclear enriched abundant transcript 1 (NEAT1) is upregulated; in CD4^+^ T cells, NEAT1 acts as a molecular sponge for miR‐144‐3p, thereby increasing Rho‐associated protein kinase 2 (ROCK2) expression, activating Wnt/β‐catenin signaling, and promoting CD4^+^ T‐cell proliferation, Th17 differentiation and chemotaxis in vitro, while NEAT1 inhibition alleviates collagen‐induced arthritis in mice [152]. lncNEAT1_2 is upregulated in childhood‐onset SLE and showed diagnostic discrimination in a pediatric cohort, supporting its status as an exploratory lncRNA biomarker candidate rather than an established diagnostic marker [130]. A recent study showed that lncTGFB2‐OT1, also annotated as NR_125715.1, is dysregulated in PBMCs from patients with RA and displays diagnostic discrimination in the tested cohort, suggesting that it may serve as an adjunct exploratory candidate for RA classification [131]. Its prognostic value, longitudinal stability, and treatment–response relevance remain unestablished [131].

At the level of m^6^A modification, peripheral blood global m^6^A content is increased in patients with RA and is negatively correlated with fat mass and obesity‐associated protein (FTO) mRNA expression [133]. The mRNA expression of α‐ketoglutarate‐dependent dioxygenase AlkB homolog 5 (ALKBH5), FTO, and YT521‐B homology domain family 2 (YTHDF2) has been reported to be reduced in the peripheral blood of patients with RA, suggesting potential alterations in m^6^A demethylation and reader‐mediated mRNA regulation [133]. The expression levels of FTO and YTHDF2 are associated with RA‐related disease‐activity or inflammatory parameters, whereas ALKBH5 expression was increased after treatment in a small subset of patients; decreased YTHDF2 expression is also associated with increased IL‐1β mRNA expression, suggesting that their expression levels may serve as exploratory molecular biomarker candidates for RA [133, 134].

In the field of functional reprogramming, disease‐relevant immune‐cell states may provide cell‐based markers for identifying pathogenic immune populations. In aquaporin‐4‐antibody‐positive neuromyelitis optica spectrum disorder (AQP4‐NMOSD), AQP4‐reactive effector/memory CD4^+^ T cells exhibit an exhaustion–like phenotype characterized by increased expression of PD‐1 and other coinhibitory receptors together with upregulation of FOXP3 [124]. The frequency of FOXP3^+^ cells within antigen‐identified AQP4‐reactive CD4^+^ T cells may serve as an exploratory cellular marker for distinguishing patients with AQP4‐NMOSD from healthy donors and disease controls [124].

### 6.2 Biomarkers for Disease Activity and Organ Involvement

Building on diagnosis, accurate assessment of disease activity and the extent of organ involvement is essential for guiding treatment intensity and preventing irreversible damage. Multidimensional biomarker candidates also show potential value in assessing disease activity and organ involvement.

Among systemic inflammation‐related biomarker candidates, elevated systemic immune‐inflammation index (SII) is associated with lower serum Klotho levels in a National Health and Nutrition Examination Survey (NHANES)‐based RA population, suggesting a possible systemic inflammatory‐burden signal. Because RA‐specific activity and serological indices such as Disease Activity Score based on 28 joints (DAS28), rheumatoid factor, anticitrullinated protein antibodies, and erythrocyte sedimentation rate (ESR) were unavailable in that analysis, this association should not be interpreted as validation of SII/Klotho as a disease‐activity biomarker [135]. In the field of immunometabolism, aconitate decarboxylase 1 (ACOD1) mRNA in PBMCs is decreased in patients with active RA compared with healthy controls. Although PBMC itaconate levels do not differ significantly among active RA, RA in remission, and healthy controls, they are negatively correlated with disease activity; together, these findings suggest an association between reduced ACOD1/itaconate‐axis activity and greater RA disease activity [137]. Because itaconate inhibits RA‐FLS proliferation and migration in vitro and ameliorates arthritis and bone erosion in animal models, reduced ACOD1/itaconate‐axis activity may be consistent with diminished metabolic restraint on RA‐FLS proliferation and migration rather than serving as a direct measure of local synovial invasion [51, 137]. Serum KYN levels and the KYN/tryptophan ratio (KYN/TRP ratio) are positively correlated with SLE disease activity. Organ‐specific associations are also reported: QA is elevated in patients with neurological involvement, KYN and the KYN/TRP ratio are increased in patients with joint involvement, and KYN, 3‐HK, 3‐HAA, and the KYN/TRP ratio are increased in patients with renal involvement. These findings suggest that KYN‐pathway metabolites may reflect disease activity and specific organ‐involvement patterns in SLE [136].

The value of epigenetic biomarkers in assessing organ involvement complements that of metabolic biomarkers. Site1 and Site2 within the IFI44L promoter remain hypomethylated during active disease in SLE, whereas methylation levels may partially recover during remission, and patients with renal involvement show even lower methylation levels [122]. Thus, IFI44L hypomethylation may reflect disease activity status and is associated with renal involvement, although its discriminatory performance for renal involvement was not evaluated [122]. In addition, in SLE, the degree of CDKN2A promoter demethylation is associated with increased anti‐dsDNA antibody and reduced complement component 3 (C3) and complement component 4 (C4), indicating that its hypomethylation may also assist in reflecting increased disease activity [129]. Renal damage–associated DNA methylation changes in CD4^+^ T cells may serve as candidate epigenetic biomarkers for proliferative lupus nephritis [60]. MMP9 promoter methylation in PBMCs is lower in SLE patients with renal involvement than in those without renal involvement [138]. Because MMP9 methylation levels are negatively correlated with creatinine and positively correlated with C3 and C4, lower MMP9 methylation may be associated with renal involvement, renal injury, and lower complement levels in SLE [138]. Promoter methylation of the runt‐related transcription factor 3 gene (RUNX3) in PBMCs is increased in SLE and further elevated in patients with renal involvement; its positive correlation with creatinine levels supports RUNX3 hypermethylation as a candidate epigenetic biomarker of renal injury and renal involvement in SLE [139].

Posttranscriptional regulatory signals may also reflect disease activity status. Elevated PBMC miR‐155 may serve as a candidate biomarker for active lupus nephritis and increased disease activity in SLE [125]. In B cells from patients with SLE, RP11‐273G15.2 expression is positively correlated with IFN‐I scores and Systemic Lupus Erythematosus Disease Activity Index (SLEDAI) scores and is associated with multiple clinical features, suggesting that it may reflect IFN‐I pathway activation in B cells and disease activity status [132]. Reduced METTL3 mRNA expression in CD4^+^ T cells from patients with SLE is accompanied by reduced global m6A levels, and METTL3 mRNA expression is negatively correlated with SLEDAI scores, suggesting its potential value as an exploratory m6A‐related molecular indicator of disease activity [89]. In PBMCs from patients with SLE, reduced ALKBH5 expression is associated with disease‐activity‐related laboratory parameters, including increased neutrophil percentage and neutrophil‐to‐lymphocyte ratio (NLR) as well as reduced C3, suggesting that low ALKBH5 may represent a candidate molecular indicator of a more active autoimmune state [153].

### 6.3 Biomarkers for Prognosis, Relapse, and Disease Trajectory

The ability to identify patients at high risk of relapse and intervene early is critical for improving long‐term outcomes, and multidimensional biomarker candidates provide potential tools to meet this need, although most require longitudinal and external validation before they can guide clinical decisions.

Elevated SII may serve as an exploratory candidate biomarker for prognosis in RA, as it is associated with increased all‐cause and cardiovascular mortality [140]. A four‐metabolite panel consisting of lysine, asparagine, isoleucine, and leucine can distinguish relapse from stable remission, supporting its value as an exploratory candidate biomarker for relapse status in relapsing‐remitting MS [141]. Dynamic DNA methylation changes in active SLE are associated with remission status and may help identify remission‐related patient subgroups [142].

Serum EV‐miR‐557 in autoimmune hepatitis is associated with relapse risk and may serve as an exploratory candidate biomarker of relapse [143]. Activated CD4^+^ T peripheral helper cells, activated CD8^+^ T cells, and B‐cell activating factor are also associated with relapse after immunosuppression withdrawal in autoimmune hepatitis, supporting their potential value as exploratory candidates for relapse‐risk stratification [144]. MMP9 was identified as an m^6^A‐related hub gene in SLE, and Mendelian randomization suggested a possible causal association between higher MMP9 expression and increased ischemic stroke risk, indicating potential relevance to complication‐risk assessment, although validation in SLE cohorts is needed [126].

### 6.4 Biomarkers for Treatment Response and Patient Stratification

On the basis of prognostic assessment, applying molecular biomarkers to guide therapeutic choices and enable patient stratification is a central goal of precision medicine.

Circulating multiomics and immunometabolic biomarkers have shown progress in predicting treatment response and supporting patient stratification. A longitudinal plasma proteomic study in RA showed that distinct protein signatures can predict responses to different conventional synthetic disease‐modifying antirheumatic drug (csDMARD) combinations, supporting the value of proteome‐based models for treatment–response prediction and therapeutic optimization [145]. In a retrospective cohort of patients with RA receiving an etanercept‐containing treatment regimen, those who achieved a favorable response had lower pretreatment SII levels, and baseline SII was significantly associated with treatment efficacy; therefore, baseline SII may serve as an exploratory candidate predictor of response to this regimen [146]. Increases in plasma itaconate are associated with improvement in Disease Activity Score based on 44 joints (DAS44) and reductions in C‐reactive protein (CRP) in patients with early RA receiving csDMARD treatment. Therefore, changes in plasma itaconate may serve as an exploratory candidate biomarker of treatment‐associated improvement and reduced inflammatory activity [147].

Posttranscriptional regulatory signals are increasingly being explored as candidate tools for patient stratification, but most remain exploratory, pharmacodynamic, or computational models rather than clinically validated predictors. Adalimumab and etanercept are associated with distinct miR‐155 expression patterns in RA monocyte‐derived M2‐like macrophages, a difference that is consistent with partial restoration of M2‐like macrophage polarization by adalimumab; therefore, miR‐155 expression may serve as an exploratory pharmacodynamic indicator reflecting differential monocyte/macrophage responses to these TNF inhibitors [148]. In RA, m^6^A modification patterns are associated with distinct immune microenvironmental features and differential response to infliximab. In particular, patients with lower m^6^A score show greater therapeutic benefit from infliximab, suggesting that the m^6^A score model may serve as an exploratory computational predictor of infliximab response and support patient stratification [149].

Functional reprogramming biomarkers provide key information for evaluating immune‐cell responses and supporting patient stratification across immunomodulatory therapeutic contexts. In a five‐patient compassionate‐use series, anti‐CD19 CAR‐T therapy led to B‐cell depletion, and all patients with refractory SLE achieved clinical remission, followed by reconstitution with predominantly naïve B cells bearing non‐class‐switched BCRs; therefore, B‐cell depletion and reconstitution can be described as pharmacodynamic response indicators after anti‐CD19 CAR‐T therapy, but they should not yet be presented as validated biomarkers for selecting patients or predicting long‐term outcomes [8]. Cotransduction of Foxp3 and an antigen‐specific T‐cell receptor (TCR) can reprogram conventional CD4^+^ T cells into antigen‐specific Treg‐like cells and reduce antigen‐induced knee swelling in an arthritis animal model; however, a significant reduction in Th17 cells in the draining lymph nodes was observed only with TCR‐transduced natural Tregs. Accordingly, the persistence and functional status of engineered Tregs are best described as preclinical pharmacodynamic readouts in experimental arthritis models rather than clinical biomarkers [154].

### 6.5 Translational Challenges and Future Directions of Biomarker Integration

Despite rapid progress, several challenges still limit clinical translation. First, most biomarkers have been evaluated within a single disease or a single specimen type, making cross‐disease comparison difficult. Second, many studies remain cross‐sectional and therefore cannot determine whether a biomarker predicts future flare, organ involvement, or treatment response. Third, preanalytical variation remains a major obstacle, especially for metabolite‐ and RNA‐based markers; the main sources include specimen type, storage conditions, and assay platform.

A further problem is the limited explanatory power of any single biomarker in AD. Given the multidimensional nature of disease heterogeneity, composite panels may be more useful than single markers, particularly when they combine metabolic, epigenetic, RNA‐based, and cellular‐state readouts. For translation, the priority should shift from adding more isolated candidates to building practical biomarker frameworks that connect mechanism, longitudinal monitoring, and treatment matching.

Biomarkers from immunometabolism, epigenetic remodeling, posttranscriptional regulation, and functional reprogramming do not describe the same layer of disease biology; together, they provide complementary views of AD. Their ultimate clinical value will depend on whether they can be integrated into robust models for diagnosis, activity assessment, prognosis, and precision therapeutic stratification. In this sense, biomarkers should be viewed not simply as molecular correlates of disease, but as tools for converting multidimensional heterogeneity into individualized clinical decisions.

## 7 Emerging Therapeutic Strategies for Precision Intervention in ADs

Therapeutic strategies for ADs are beginning to shift from broad immunosuppression toward mechanism‐guided intervention [155]. Emerging evidence indicates that immunometabolic remodeling, epigenetic dysregulation, posttranscriptional control, and immune‐cell functional plasticity provide new opportunities for pathway‐matched precision therapy [21, 156, 157, 158].

The central challenge in precision intervention is that the functional consequences of targeting the same pathway may vary across diseases, tissues, and immune‐cell states. The clinical value of these emerging therapies should therefore be judged less by whether they target a new molecule and more by whether they match a dominant regulatory circuit to a defined disease endotype, organ‐involvement pattern, or biomarker‐informed patient subset. Within this framework, each therapeutic class addresses a different layer of regulation (Table 2): immunometabolic therapies exploit metabolic vulnerabilities, epigenetic therapies may loosen stabilized pathogenic programs, RNA‐directed strategies act on transcript fate, and functional reprogramming approaches aim to reset pathogenic immune‐cell states. We systematically curated ongoing clinical trials registered on ClinicalTrials.gov over the past 5 years investigating therapeutic agents targeting immunometabolism, epigenetic regulation, posttranscriptional regulation, or functional reprogramming in ADs. In total, 245 trials were identified, including 26 related to immunometabolism‐targeted therapies and 219 related to functional reprogramming‐based therapies. No trials targeting posttranscriptional or epigenetic regulation were identified based on our criteria. Detailed information is provided in Table S1.

**TABLE 2:** Targeted therapeutic strategies for autoimmune diseases classified by regulatory layer and stage of evidence.

Therapeutic strategy category	Therapeutic strategy/drug	Target/mechanism	Autoimmune disease (s)	Trial phase/status	Key findings/outcome	References
Immunometabolism‐targeted	2‐DG	Glycolysis blockade	RA	Preclinical (CIA mice)	Reduced macrophage activation, inflammatory infiltration, and joint destruction	[159]
	Pkm2 RNAi, shPkm2 plasmid	PKM2 (STAT1‐related inflammatory program)	RA	Preclinical (PIA rats)	Alleviated experimental arthritis and suppressed inflammatory cytokines and STAT1 activation	[32]
	Sirolimus	mTORC1 (effector T‐cell glycolysis and Treg regulation)	SLE	Clinical study (retrospective single‐center study)	Improved disease activity and corrected T‐cell subset imbalance	[160]
	4‐OI	Itaconate pathway (inflammation and oxidative‐stress regulation)	SLE	Preclinical (NZB/W F1 lupus mice)	Attenuated immune dysregulation and organ damage	[52, 161]
	DMF (BG‐12)	NRF2 activation; GAPDH/glycolysis inhibition	RRMS	Clinical trial evidence, including Phase 3	Reduced relapses and MRI disease activity	[162, 163, 164, 165, 166]
	Vidofludimus calcium	DHODH (de novo pyrimidine synthesis)	RRMS	Clinical trial (Phase 2)	Reduced new MRI lesions with favorable safety and tolerability	[167]
	Metformin	Mitochondrial metabolic regulation	SLE	Clinical study (posthoc pooled analysis of two RCTs)	Reduced subsequent flare risk in patients with low disease activity	[34, 168]
	Ketogenic diet	Nutritional ketosis, systemic metabolic remodeling	Relapsing MS	Clinical trial (Phase 2)	Safe and tolerable, with potential clinical benefits	[169]
	Acriflavine	HIF‐1 inhibition and UPR/eIF2α–ATF4 modulation	MS‐associated ON	Preclinical (EAE‐ON mice)	Preserved visual function and reduced optic‐nerve inflammation and demyelination	[170]
	Ilepcimide	DHODH inhibition and NRF2 activation (restricting inflammation and oxidative stress)	MS	Preclinical (MOG‐induced EAE mice)	Reduced EAE clinical scores, leukocyte infiltration, demyelination, blood–brain barrier leakage, and CD4^+^ and CD8^+^ T‐cell infiltration/activation	[171]
Epigenetic‐targeted	ACY‐738	HDAC6 inhibition	SLE	Preclinical (NZB/W lupus mice)	Improved renal pathology, reduced proteinuria, anti‐dsDNA and renal immune‐complex deposition, and prolonged survival	[172]
	Givinostat (ITF2357)	HDAC inhibition	SoJIA	Clinical trial (Phase 2, open‐label, international, multicenter)	Showed favorable clinical responses and tolerability	[173]
	CKD‐506	HDAC6 inhibition	RA	Preclinical (AIA rats; RA PBMC and FLS in vitro studies)	Suppressed macrophage and FLS inflammation, improved Treg function, and reduced arthritis severity	[174]
	Vorinostat	HDAC inhibition (suppresses IFN‐I and plasma‐cell differentiation)	SLE	Preclinical (NZB/W F1 lupus mice; human PBMC and B‐cell studies)	Inhibited IFN‐I and plasma‐cell differentiation, improved lupus nephritis, and prolonged survival	[175]
	Pargyline	LSD1 inhibition (H3K9 demethylation, adipogenic program)	TAO	Preclinical (patient‐derived cells, in vitro)	Inhibited adipogenic differentiation of TAO‐derived cells	[176]
	GSK126	EZH2, PRC2	SLE, LN	Preclinical (NZB/NZW F1 mice)	Prolonged survival, reduced anti‐dsDNA, and attenuated proteinuria and lupus nephritis	[177]
Posttranscriptional/RNA‐targeted	Coptisine	METTL14 (m^6^A‐mediated TSC1 mRNA stabilization)	UC	Preclinical (DSS colitis mice; macrophage studies)	Stabilized TSC1 mRNA; shifted M1/M2 macrophage polarization	[156]
	LNA anti‐miR‐21, anti‐miR‐29a, and anti‐miR‐29b cocktail	Exosomal miR‐21, miR‐29a, and miR‐29b (TLR7 and TLR8‐related inflammatory signaling)	SLE	Preclinical (humanized SLE PBMC‐transfer mice)	Reduced proinflammatory cytokines and target‐organ inflammatory infiltration	[178]
	miR‐155 inhibitor	miR‐155, SOCS1	SLE	Preclinical (SLE mouse model; Treg in vitro studies)	Improved Treg function and stability and alleviated SLE phenotypes	[179]
	miR‐122‐5p inhibitor‐transfected SLE exosomes	miR‐122‐5p, FOXO3, NF‐κB (macrophage M1 polarization)	SLE, LN	Preclinical (MRL/lpr mice)	Improved albuminuria, creatinine, C3, kidney/lung pathology	[180]
	anti‐miR‐21a‐5p	miR‐21a‐5p, STAT3, PTEN	SSc	Preclinical (BLM‐induced SSc mice)	Reduced inflammatory infiltration and attenuated skin and lung fibrosis	[181]
	Ginger EVs@ZIF‐8@TNF‐α siRNA	TNF‐α siRNA (colon‐targeted oral delivery)	UC	Preclinical (DSS colitis mice)	Reduced colonic inflammation, promoted epithelial repair, and restored gut microbial balance	[182]
	Tolerogenic m^1^Ψ‐modified autoantigen mRNA‐LPX vaccine	m^1^Ψ‐mRNA‐LPX (antigen‐specific Treg induction, immune tolerance)	MS	Preclinical (multiple EAE mouse models)	Induced antigen‐specific FoxP3^+^ Treg cells, suppressed EAE, and preserved unrelated immune responses	[183]
	HIF‐CaP‐rHDL	HIF‐1α siRNA (anti‐inflammatory RNA silencing)	RA	Preclinical (CIA mice; RAW 264.7 studies)	Knocked down HIF‐1α, inhibited NF‐κB and MAPK signaling, and reduced RA inflammation	[184]
	miR‐20a‐overexpressing ADSCs	miR‐20a, mTOR‐mediated autophagy regulation	SLE, LN	Preclinical (B6.MRL/lpr mice)	Improved serologic and histologic abnormalities and protected podocytes	[185]
	HSP10 mRNA‐loaded LNPs	HSP10 mRNA delivery via heparin and protamine‐modified LNPs	RA	Preclinical (CIA DBA1/J mice)	Reduced joint inflammation and deformity and increased splenic Treg cells in CIA mice	[186]
	HCQ and TNF‐α siRNA LNPs	TNF‐α siRNA and HCQ (intra‐articular LNP codelivery)	RA	Preclinical (AIA rats; RAW 264.7 studies)	Reduced TNF‐α, paw volume, arthritis scores; good biosafety	[187]
Functional reprogramming‐based	CD19 CAR‐T	CD19 (B‐cell immune reset)	SLE, IIM, SSc	Clinical (multidisease case series)	Induced DORIS remission in SLE, major clinical response in IIM, reduced disease activity in SSc, and allowed discontinuation of immunosuppressive therapy in this case series	[155]
	Zorpo‐cel	CD19	SLE, SSc, IIM	Clinical trial (Phase 1–2a basket trial)	Met safety and efficacy endpoints; all patients remained free of GCs and other immunosuppressants during follow‐up	[188]
	CD19‐targeting CAR‐T	CD19 (B‐cell depletion, immune reset)	Diffuse SSc	Clinical (case series)	No progression; improved mRSS, CT disease extent, and FVC	[189]
	Relma‐cel	CD19	Active SLE	Clinical trial (Phase 1, open‐label)	All patients achieved SRI response; some reached DORIS remission; CRS was mostly grade 1	[190]
	BCMA‐CD19 cCAR	BCMA, CD19 (dual humoral and B‐cell immune reset)	SLE, LN	Clinical trial (Phase 1, open‐label)	Reduced B cell‐ and plasma cell‐derived autoantibodies	[191]
	Descartes‐08	BCMA (transient mRNA CAR‐T)	gMG	Clinical trial (Phase 1b–2a; Phase 2b RCT)	Produced durable benefit without lymphodepletion; Phase 2b showed superior MGC response versus placebo	[192, 193]
	Anti‐BCMA and anti‐CD19 bispecific CAR‐T cells	BCMA, CD19	Refractory gMG	Clinical trial (Phase 1)	Showed favorable safety and efficacy; most patients achieved minimal manifestations	[194]
	Blinatumomab	CD19×CD3 BiTE (T‐cell redirection to eliminate B cells)	Multidrug‐refractory RA	Clinical (compassionate‐use case series)	Reduced disease activity, improved imaging synovitis, and induced B‐cell immune reset	[195]
	T‐IL10 mRNA	IL‐10 mRNA, Ly6C^+^ inflammatory leukocyte targeting	IBD	Preclinical (DSS colitis mice)	Targeted IL‐10 mRNA expression in Ly6C^+^ leukocytes; alleviated colitis	[196]
	Simvastatin	Th17 and Treg balance (anti‐inflammatory and immunomodulatory effects)	MS	Preclinical (EAE mice)	Alleviated EAE by regulating Th17 and Treg balance	[197]
	STA‐21	STAT3 inhibition (Th17 and Treg balance)	MS	Preclinical (EAE mice)	Reduced EAE severity, inflammation, demyelination, STAT3 phosphorylation, and Th17 responses while increasing Treg and IL‐10 responses	[198]
	Ivermectin	IL‐2, STAT5 pathway (Th17 and Treg balance)	MS	Preclinical (EAE mice)	Protected against EAE through IL‐2/STAT5‐mediated Th17 and Treg modulation	[199]
	Silybin	DC activation and Th17 differentiation	MS	Preclinical (EAE mice)	Suppressed DC activation and Th17 differentiation, thereby alleviating EAE	[200]
	Parthenolide	Th17 suppression, NF‐κB–RORγt pathway	MS	Preclinical (EAE mice)	Suppressed Th17 responses and NF‐κB–RORγt signaling, thereby alleviating EAE	[201]
	Baricitinib, fedratinib	JAK2 signaling, STAT5‐mediated T‐cell polarization	MS	Preclinical (EAE mice)	Reduced Th1 and Th17 responses and alleviated EAE disease activity	[202]
	Pentamidine	S100B‐associated neuroinflammatory signaling	MS	Preclinical (chronic EAE mice)	Improved recovery, reduced neuroinflammation, and increased spinal‐cord Treg density	[203]
	AD‐MSCs	MSC‐mediated Th1 and Th17 suppression, Th2 and Treg expansion	MS	Preclinical (EAE mice)	Reduced Th1 and Th17 responses, expanded Th2 and Treg responses, and ameliorated EAE	[204]
	VitD_3_‐tolDCs and IFN‐β	Antigen‐specific tolDCs, IFN‐β‐supported immune regulation	MS	Preclinical (C57BL/6 EAE mice; RRMS patient‐cell studies)	Combined therapy improved EAE more effectively than either monotherapy	[205]
	Autologous AD‐MSCs	MSC‐based immunomodulation	RA	Clinical trial (Phase 1–2a, open‐label, nonrandomized pilot trial)	Showed safety and improved joint‐count outcomes	[206]
	Allogeneic BM‐MSCs	MSC‐based immunomodulation and tissue repair	Rectovaginal fistulizing CD	Clinical trial (Phase 1B–2A, RCT)	No MSC‐related AEs or SAEs; 50% achieved complete clinical and radiographic healing at 6 months	[207]
	Metformin‐loaded CuS@MnO_2_ NP‐modified MSCs	MSC nanomodification (enhanced anti‐inflammatory and chondrogenic activity)	RA	Preclinical (two RA rat models)	Enhanced MSC survival, migration, anti‐inflammatory activity, and chondrogenesis; reduced synovitis and cartilage erosion	[208]
	CXCR4‐expressing MSC membrane‐coated MTX‐loaded PLGA nanoparticles	CXCR4/SDF‐1 axis, inflamed‐joint targeting	RA	Preclinical (CIA mice)	Enhanced inflamed‐joint accumulation and suppressed synovial inflammation with favorable safety	[209]

Abbreviations: 2‐DG, 2‐deoxy‐D‐glucose; 4‐OI, 4‐octyl itaconate; AD‐MSCs, adipose‐derived mesenchymal stem cells; ADSCs, adipose‐derived stem cells; AEs, adverse events; AIA, adjuvant‐induced arthritis; anti‐dsDNA, anti‐double‐stranded DNA antibody; ATF4, activating transcription factor 4; B6.MRL/lpr, C57BL/6‐congenic MRL/lpr mouse strain; BCMA, B‐cell maturation antigen; BiTE, bispecific T‐cell engager; BLM, bleomycin; BM‐MSCs, bone marrow‐derived mesenchymal stem cells; C3, complement component 3; C57BL/6, C57BL/6 mouse strain; CaP, calcium phosphate; CAR‐T, chimeric antigen receptor T‐cell therapy; cCAR, compound chimeric antigen receptor; CD, Crohn's disease; CD19, cluster of differentiation 19; CD3, cluster of differentiation 3; CD4, cluster of differentiation 4; CD8, cluster of differentiation 8; CIA, collagen‐induced arthritis; CRS, cytokine release syndrome; CT, computed tomography; CuS, copper sulfide; CXCR4, C‐X‐C motif chemokine receptor 4; DBA1/J, DBA/1J mouse strain; DC, dendritic cell; DHODH, dihydroorotate dehydrogenase; DMF/BG‐12, dimethyl fumarate; DORIS, Definition of Remission in Systemic Lupus Erythematosus; DSS, dextran sulfate sodium; EAE, experimental autoimmune encephalomyelitis; EAE‐ON, experimental autoimmune encephalomyelitis‐associated optic neuritis; eIF2α, eukaryotic translation initiation factor 2α; EVs, extracellular vesicles; EZH2, enhancer of zeste homolog 2; FLS, fibroblast‐like synoviocyte; FOXO3, forkhead box O3; FoxP3, forkhead box P3; FVC, forced vital capacity; GAPDH, glyceraldehyde‐3‐phosphate dehydrogenase; GCs, glucocorticoids; gMG, generalized myasthenia gravis; H3K9, histone H3 lysine 9; HCQ, hydroxychloroquine; HDAC, histone deacetylase; HDAC6, histone deacetylase 6; HIF‐1, hypoxia‐inducible factor 1; HIF‐1α, hypoxia‐inducible factor 1α; HIF‐CaP‐rHDL, HIF‐1α siRNA‐loaded calcium phosphate/reconstituted high‐density lipoprotein nanoparticle; HSP10, heat shock protein 10; IBD, inflammatory bowel disease; IFN‐I, Type I interferon; IFN‐β, interferon‐β; IIM, idiopathic inflammatory myopathy; IL‐10, interleukin‐10; IL‐2, interleukin‐2; ITF2357, givinostat; JAK2, Janus kinase 2; LN, lupus nephritis; LNA, locked nucleic acid; LNP/LNPs, lipid nanoparticle(s); LPX, lipoplex; LSD1, lysine‐specific demethylase 1; Ly6C, lymphocyte antigen 6 complex locus C; M1/M2, classically/alternatively activated macrophage phenotypes; m^1^Ψ, N^1^‐methylpseudouridine; m^6^A, N^6^‐methyladenosine; MAPK, mitogen‐activated protein kinase; METTL14, methyltransferase‐like 14; MGC, Myasthenia Gravis Composite; miR, microRNA; MnO_2_, manganese dioxide; MOG, myelin oligodendrocyte glycoprotein; MRI, magnetic resonance imaging; MRL/lpr, Murphy Roths Large/lymphoproliferation mouse strain; mRNA, messenger RNA; mRNA‐LPX, messenger RNA lipoplex; mRSS, modified Rodnan skin score; MS, multiple sclerosis; MSC/MSCs, mesenchymal stem cell(s); mTOR, mechanistic target of rapamycin; mTORC1, mechanistic target of rapamycin complex 1; MTX, methotrexate; NF‐κB, nuclear factor kappa B; NP/NPs, nanoparticle(s); NRF2, nuclear factor erythroid 2‐related factor 2; NZB/NZW F1 and NZB/W F1, New Zealand Black/New Zealand White F1 mouse strain; ON, optic neuritis; PBMC/PBMCs, peripheral blood mononuclear cell(s); PIA, pristane‐induced arthritis; PKM2, pyruvate kinase M2; Pkm2, pyruvate kinase M2 gene; PLGA, poly(lactic‐co‐glycolic acid); PRC2, polycomb repressive complex 2; PTEN, phosphatase and tensin homolog; RA, rheumatoid arthritis; RAW 264.7, murine macrophage cell line; RCT/RCTs, randomized controlled trial(s); Relma‐cel, relmacabtagene autoleucel; rHDL, reconstituted high‐density lipoprotein; RNA, ribonucleic acid; RNAi, RNA interference; RORγt, retinoic acid receptor‐related orphan receptor γt; RRMS, relapsing‐remitting multiple sclerosis; S100B, S100 calcium‐binding protein B; SAEs, serious adverse events; SDF‐1, stromal cell‐derived factor 1; shPkm2, short hairpin RNA targeting Pkm2; siRNA, small interfering RNA; SLE, systemic lupus erythematosus; SOCS1, suppressor of cytokine signaling 1; SoJIA, systemic‐onset juvenile idiopathic arthritis; SRI, SLE responder index; SSc, systemic sclerosis; STAT1, signal transducer and activator of transcription 1; STAT3, signal transducer and activator of transcription 3; STAT5, signal transducer and activator of transcription 5; TAO, thyroid‐associated ophthalmopathy; Th1, T helper 1 cell; Th17, T helper 17 cell; Th2, T helper 2 cell; T‐IL10 mRNA, targeted interleukin‐10 messenger RNA; TLR7, Toll‐like receptor 7; TLR8, Toll‐like receptor 8; TNF‐α, tumor necrosis factor‐α; tolDCs, tolerogenic dendritic cells; Treg, regulatory T cell; TSC1, tuberous sclerosis complex 1; UC, ulcerative colitis; UPR, unfolded protein response; VitD_3_, vitamin D_3_; ZIF‐8, zeolitic imidazolate framework‐8; Zorpo‐cel, zorpocabtagene autoleucel.

### 7.1 Immunometabolism‐Targeted Therapies: Exploiting Metabolic Vulnerabilities

Immunometabolic intervention is attractive because pathogenic effector cells and regulatory immune cells often depend on different metabolic programs. This difference creates a possible window for selective therapeutic modulation [21, 23]. Glycolysis provides a useful entry point for this idea. In collagen‐induced arthritis, the glycolytic inhibitor 2‐deoxy‐D‐glucose (2‐DG) reduced macrophage activation, inflammatory cell infiltration, and joint destruction. This suggests that glycolytic blockade can affect autoimmune arthritis at both immune and stromal levels [159]. Targeting PKM2 produced a similar pattern: experimental arthritis was alleviated and STAT1 activation in macrophages was reduced, supporting PKM2 as a potential metabolic target in RA [32]. Taken narrowly, these data point to glycolytic inhibition as most relevant for disease states in which inflammatory macrophages or effector T‐cell programs dominate. A major limitation is that systemic glycolytic inhibition could also affect protective immune functions and nonimmune tissues, making clinical translation less straightforward.

Among metabolic approaches, mTOR inhibition is closer to clinical application. Sirolimus targets mTOR‐dependent T‐cell dysregulation, and in a retrospective clinical study of mild‐to‐moderate SLE, add‐on sirolimus was associated with improved disease activity, increased Treg numbers, a higher Treg/Th17 ratio, and broader correction of T‐cell subset imbalance [160]. This provides preliminary clinical evidence that metabolic pathway modulation may have therapeutic value in SLE [160]. Such intervention is likely to be most relevant when persistent T‐cell activation is paired with deficient regulatory restraint.

Other examples also support the feasibility of targeting immunometabolism therapeutically. Modulation of the itaconate pathway attenuated murine lupus, consistent with the anti‐inflammatory role of metabolite‐dependent signaling brakes [52, 161]. Dimethyl fumarate combines NRF2 activation with effects on aerobic glycolysis and has already been clinically validated in MS. It therefore offers an example of metabolic intervention moving from mechanistic rationale to established therapy [162, 163, 164, 165, 166]. Likewise, the selective dihydroorotate dehydrogenase inhibitor vidofludimus calcium reduced MRI lesion activity in a phase 2 trial of relapsing‐remitting MS, further supporting metabolism‐directed immunomodulation [167]. Post hoc analyses of randomized trials suggest that metformin may reduce disease flares in SLE. This finding supports the possible therapeutic relevance of repurposed metabolic modulators in SLE [168]. These findings make the strongest case for immunometabolic therapy when treatment is directed at a dominant metabolic dependency within a well‐defined inflammatory endotype, rather than used as broad metabolic suppression.

### 7.2 Epigenetic‐Targeted Therapies: Resetting Stabilized Pathogenic States

Epigenetic therapies are attractive because they target regulatory programs that help maintain chronic inflammatory states. In ADs, the aim is not only to suppress inflammation but also to correct disease‐associated epigenetic abnormalities [157].

Among chromatin‐ and deacetylase‐directed approaches, histone deacetylase (HDAC) inhibitors are among the most developed. The available evidence suggests that selectivity may be important, but HDAC6‐targeted strategies should be interpreted partly as deacetylase‐directed immunomodulation rather than as purely chromatin‐level epigenetic resetting [174]. In lupus‐prone mice, the HDAC6 inhibitor ACY‐738 improved renal pathology, reduced proteinuria and autoantibody titers, and prolonged survival. These results support selective HDAC6 targeting as a way to ameliorate lupus‐like disease [172]. Separately, the HDAC inhibitor givinostat completed a Phase 2 study with favorable clinical responses and tolerability in systemic‐onset juvenile idiopathic arthritis. This provides one of the clearer clinical signals that epigenetic intervention may improve autoimmune inflammation [173]. More recently, the selective HDAC6 inhibitor CKD‐506 suppressed inflammatory responses in RA‐related immune and stromal cells, improved Treg suppressive function, and reduced arthritis severity in vivo. These effects suggest that selective HDAC inhibition may be especially relevant to synovitis involving combined macrophage, fibroblast, and Treg dysfunction [174].

Beyond selective HDAC6 inhibition, this therapeutic logic extends to broader HDAC inhibition and other epigenetic targets. A repurposing study identified vorinostat as a compound that suppresses IFN‐I production and plasma‐cell differentiation in experimental SLE settings [175]. This implies that some epigenetic therapies may act on more than one core disease axis at the same time [175]. Inhibition of lysine‐specific demethylase 1 suppressed adipogenic differentiation in thyroid‐associated ophthalmopathy‐derived orbital adipose cells [176]. This places epigenetic intervention within tissue‐remodeling phenotypes rather than only immune‐cell inflammation [176]. Likewise, the EZH2 inhibitor GSK126 improved survival, reduced anti‐dsDNA titers, and alleviated lupus nephritis in New Zealand Black/New Zealand White (NZB/NZW) F1 mice. These findings support EZH2 inhibition as another epigenetic therapeutic entry point, particularly for IFN‐I‐linked lupus nephritis [177]. These studies point to a likely niche for epigenetic therapy: diseases in which pathogenic cell states appear stabilized and are not adequately controlled by conventional immunosuppression. The main unresolved issues are selectivity, off‐target effects, and how to identify the patient subsets most likely to benefit.

### 7.3 Posttranscriptional and RNA‐Directed Therapies: Targeting Transcript Fate With Higher Precision

Posttranscriptional therapies work at the level of RNA stability, RNA modification, or transcript‐specific silencing. Their potential advantage lies in precision: RNA‐level mechanisms are often cell‐ and context‐dependent, unlike pathway‐wide immunosuppression. One branch of this field targets m^6^A‐related regulation. Coptisine alleviated dextran sulfate sodium (DSS)‐induced colitis by increasing methyltransferase‐like 14 (METTL14)‐dependent m^6^A modification of tuberous sclerosis complex 1 (TSC1) mRNA, stabilizing TSC1 transcripts, suppressing mitogen‐activated protein kinase kinase (MEK)/ERK‐associated M1 macrophage polarization, and promoting M2 polarization [156]. This example makes RNA‐modification therapy most relevant to settings in which altered transcript handling can be traced to a defined cell lineage.

A second branch acts through pathogenic miRNAs or RNA interference‐mediated silencing of inflammatory genes. Antagonizing disease‐associated exosomal miRNAs, including miR‐21, miR‐29a, and miR‐29b, reduced inflammatory infiltration in target organs in lupus‐related humanized mice, whereas miR‐155 inhibition preserved suppressor of cytokine signaling 1 (SOCS1)‐dependent Treg stability and function and alleviated lupus phenotypes [178, 179]. Targeting SLE exosomal miR‐122‐5p also improved lupus nephritis by reducing forkhead box O3 (FOXO3)/NF‐κB‐mediated M1 macrophage polarization [180]. Anti‐miR‐21a‐5p reduced infiltration of inflammatory cytokine‐producing cells in the skin and attenuated skin and lung fibrosis in a murine systemic sclerosis model, in association with reduced STAT3 phosphorylation and increased PTEN expression in the skin; this suggests that anti‐miR strategies may be useful when immune activation and tissue remodeling are tightly linked [181]. RNA interference‐based delivery systems provide a complementary route. For example, oral delivery of TNF‐α small interfering RNA through a ginger‐derived EV‐coated nanoplatform ameliorated experimental UC and restored gut microbial homeostasis in mice, suggesting that tissue‐directed transcript silencing may be particularly relevant in mucosal ADs [182].

Among RNA‐based approaches, tolerogenic mRNA‐lipid nanoparticle vaccination is perhaps the most conceptually distinct. A noninflammatory N^1^‐methylpseudouridine (m^1^Ψ)‐modified mRNA vaccine encoding a disease‐relevant autoantigen induced antigen‐specific Foxp3^+^ Treg cells and suppressed disease in multiple EAE models without overt systemic immune suppression [183]. This work shifts the interpretation of mRNA platforms: they can be used not only to stimulate immunity but also to induce antigen‐specific tolerance. Posttranscriptional and RNA‐directed therapies are most compelling when the relevant disease‐driving transcript, miRNA, or autoantigen can be clearly identified. The translational barriers are still substantial: delivery specificity and platform immunogenicity remain unresolved, and durability and reproducibility across heterogeneous patient populations still need to be shown.

### 7.4 Functional Reprogramming‐Based Therapies: Re‐Educating Immune‐Cell States

Functional reprogramming‐based therapies try to move differentiated immune cells away from pathogenic phenotypes and toward regulatory or nonpathogenic states. Because these strategies act at the level of cell‐state output, they may produce remission that is deeper or more durable than approaches focused only on upstream signal dampening. Cell‐based strategies provide direct examples of functional reprogramming. In RA, autologous DCs conditioned by NF‐κB inhibition and loaded with citrullinated peptides have been explored as immunomodulatory APCs that reshape the downstream balance between effector T cells and Tregs, whereas in a humanized SLE model, FOXP3‐overexpressing CD19 CAR‐Tregs represent an engineered regulatory‐cell state that suppresses CD19^+^ B‐cell‐mediated autoimmune responses [158, 210].

In B‐cell‐mediated autoimmunity, cellular reconstitution has moved especially quickly. CD19 CAR‐T therapy has shown favorable safety and drug‐free remission potential across multiple treatment‐refractory ADs, with prospective Phase 1/2a support from the CAR‐T cells in systemic B cell‐mediated autoimmune disease (CASTLE) basket trial [155, 188]. Additional case‐series and early‐phase studies report benefit in diffuse systemic sclerosis and active SLE. These data suggest that CAR‐T‐mediated immune reset may extend beyond classic antibody‐driven disease and may also affect fibrosis‐associated organ injury [189, 190]. Dual‐target B‐cell maturation antigen (BCMA)/CD19 CAR‐T strategies may deepen remission by eliminating both autoreactive B cells and antibody‐secreting plasma cells. This could allow a more complete humoral and B‐cell immune reset [191]. Dual‐target BCMA/CD19 CAR‐T therapy has also shown encouraging efficacy in refractory myasthenia gravis [194].

RNA‐engineered CAR‐T platforms and non‐CAR T‐cell retargeting therapies extend the same immune‐reset logic. Anti‐BCMA RNA CAR‐T cells demonstrated durable clinical benefit in generalized myasthenia gravis without lymphodepleting pretreatment, and randomized Phase 2b data further support the feasibility of transient mRNA CAR‐T technology in AD [192, 193]. At the same time, the CD19×cluster of differentiation 3 (CD3) bispecific T‐cell engager blinatumomab has produced clinical and immunological improvement in multidrug‐refractory RA. This suggests that T‐cell redirection may be another route to immune functional recalibration [195].

Functional reprogramming does not necessarily require adoptive cell transfer. Targeted delivery of modified mRNA encoding IL‐10 to lymphocyte antigen 6 complex locus C (Ly6C)‐positive inflammatory leukocytes alleviated DSS‐induced colitis. This provides proof of concept that inflammation‐site‐directed mRNA therapy can re‐educate pathogenic leukocytes in vivo [196]. These approaches point to a likely place for functional reprogramming: severe or refractory disease states maintained by stable pathogenic immune circuits. Their appeal lies in the depth and specificity of immune reset. The main concerns are long‐term immune consequences, together with practical barriers such as cost and manufacturing.

Taken together, the studies reviewed above suggest a potential limitation: these strategies may not work equally well across ADs or across all patients within one disease. Their value is likely to be highest when treatment choice is matched to dominant regulatory circuits identified through multidimensional biomarkers. Different biomarker classes would then guide different therapeutic choices: metabolic signatures for immunometabolic intervention, epigenetic states for chromatin‐directed therapy against stabilized pathogenic programs, RNA‐related biomarkers for transcript‐level drivers, and immune‐cell state markers for functional reprogramming approaches.

The same framework also argues for more rational combination strategies. For example, weakening a dominant pathogenic pathway may not be enough on its own; it could become more effective when paired with a strategy that restores regulatory function or limits tissue‐specific inflammatory amplification. Ideally, combination therapy should be designed around complementary targeting of the dominant molecular and cellular abnormalities in a defined patient subset, rather than around empirical drug stacking.

Despite rapid progress, many of these strategies are still preclinical or in early clinical testing. Several bottlenecks still limit translation, including patient selection, optimal timing and dosing, tissue‐ or cell‐specific delivery, long‐term safety, and standardization of complex platforms across centers and disease indications. Even so, the field is beginning to move beyond proof‐of‐concept. Clinical validation of dimethyl fumarate in MS, together with emerging clinical evidence for sirolimus in SLE, provides preliminary support that noncanonical regulatory pathways can be therapeutically tractable [160, 162, 163, 164, 165, 166]. In parallel, tolerogenic mRNA platforms and immune‐cell reprogramming strategies suggest a more precise form of immune recalibration [155, 183, 188, 189, 190, 192, 193, 194, 196]. Future progress will likely require closer integration of mechanistic studies, longitudinal multiomics, and biomarker‐guided patient stratification. The goal is to base treatment selection increasingly on molecular subtype rather than on a broad disease label alone, thereby building a precision‐intervention framework that links dominant pathogenic circuits, clinically actionable biomarkers, and matched therapies within a coherent translational pathway.

## 8 Conclusion and Future Perspectives

ADs show substantial heterogeneity in immune‐cell composition, dominant pathogenic pathways, organ involvement, disease course, and responses to treatment. The evidence reviewed here argues against treating autoimmune heterogeneity as the product of isolated molecular abnormalities. It is better explained by interactions among several regulatory layers, including immunometabolic rewiring, epigenetic remodeling, posttranscriptional fine‐tuning, and immune‐cell functional reprogramming. These regulatory layers intersect at several points: environmental sensing by immune and stromal cells, maintenance of pathogenic or regulatory states, processing of disease‐relevant transcripts, and generation of distinct inflammatory outputs. Autoimmune heterogeneity therefore should be viewed as a dynamic, multilayered biological process, not merely as a downstream result of immune activation.

One practical implication is that biomarkers and therapeutic strategies should be developed with their molecular context in mind. A biomarker is unlikely to be translationally useful simply because it is differentially expressed. To be clinically informative, it should reflect a dominant regulatory circuit, capture a clinically relevant cell state, or provide actionable support for diagnosis, stratification, prognosis, or treatment selection. The same logic applies to therapy: strategies are more likely to be useful when target selection is guided by key regulatory programs or reprogrammable immune‐cell states, rather than by the alteration of individual molecules alone. Moving from mechanism discovery to clinical application will therefore depend on a pipeline that connects molecular dysregulation to biomarker prioritization, patient stratification, and mechanism‐based intervention.

The mechanistic sections of this review trace autoimmune heterogeneity from molecular regulation to cellular behavior. Altered metabolic flux, chromatin status, RNA‐level control, and immune‐cell state remodeling do not provide interchangeable information. Their value lies in explaining why similar immune dysregulation may produce different tissue lesions, patterns of disease activity, and therapeutic vulnerabilities.

The biomarker and therapeutic sections then extend this mechanistic view toward clinical use. Metabolite profiles, methylation and chromatin signatures, RNA‐based markers, and reprogrammed immune‐cell phenotypes offer different readouts of disease state. In parallel, pathway‐directed metabolic, epigenetic, RNA‐directed, and cell‐state reprogramming strategies indicate how these regulatory insights might be converted into interventions. This review therefore treats ADs as network‐driven disorders, linking molecular regulation, cellular phenotypes, biomarkers, and therapies within one conceptual framework. Future work should prioritize longitudinal and cross‐disease multiomics cohorts, standardized biomarker validation, integration of single‐cell and spatial technologies, and therapeutic trials built around mechanism‐based hypotheses.

## Author Contributions

Jialong Kuang conducted the literature search, prepared the figures and tables, and drafted the manuscript. Yin Zhu and Fang Xu contributed to writing and revising the manuscript and preparing the tables. Yongjing Liu and Yuan Li contributed to manuscript writing. Ling Yuan, Liqin Wei, Bo Huang, and Yan Chen proofread and edited the manuscript. Pei Huang and Zuochen Du conceived the study and oversaw the review and revision of the manuscript. All authors read and approved the final manuscript and agree to be accountable for all aspects of the work.

## Ethics Statement

The authors have nothing to report.

## Conflicts of Interest

The authors declare no conflicts of interest.

## Supporting information




**Supporting File 1**: mco270905‐sup‐0001‐SuppMat.docx

## Data Availability

The authors have nothing to report.
