# Supplementary material for: Autoimmunity: Molecular Mechanisms, Biomarkers, and Therapeutic Opportunities
Source: MedComm (2020). 2026 Sep 3;7(9):e70905. doi: 10.1002/mco2.70905 (PMC13539207; doi:10.1002/mco2.70905)
Supplement: Supplementary file 1 [file MCO2-7-e70905-s001.docx]

**Autoimmunity: Molecular Mechanisms, Biomarkers, and Therapeutic Opportunities**

Jialong Kuang^1,2^, Yin Zhu^1,2^, Fang Xu^1,2^, Yongjing Liu^1,2^, Yuan Li^1,2^, Ling Yuan^1,2^, Liqin Wei^1,2^, Bo Huang^3^, Yan Chen^1,2,4^, Pei Huang^1,2,4 *^, Zuochen Du^1,2*^

1. Department of Pediatrics, Affiliated Hospital of Zunyi Medical University, Zunyi, China

2. Guizhou Children's Hospital, Zunyi, China

3. Zunyi Medical and Pharmaceutical College, Zunyi, China

4. Guizhou Biomanufacturing Laboratory, Affiliated Hospital of Zunyi Medical University, Zunyi, China

* Corresponding authors:

Zuochen Du, MD, Department of Pediatrics, Affiliated Hospital of Zunyi Medical University, Zunyi, Guizhou 563003, China; Tel: +86-0851-28609207; E-mail: [dzc9036@126.com](mailto:dzc9036@126.com)

Pei Huang, PhD, Department of Pediatrics, Affiliated Hospital of Zunyi Medical University, Zunyi, Guizhou 563003, China; E-mail: [fenglin4620@163.com](mailto:fenglin4620@163.com)

**Supplementary Information**

**Table S1.** Ongoing clinical trials of therapeutic strategies targeting immunometabolism, epigenetic regulation, post-transcriptional regulation, or immune-cell functional reprogramming in autoimmune diseases

| Category | NCT Number | Drug | Diseases | Study Status | Phases | Trial objective | Available preliminary / reported results |
| --- | --- | --- | --- | --- | --- | --- | --- |
| Functional reprogramming | NCT07629596 | Arnovie101 | Systemic Lupus Erythematosus\|Autoimmune Hemolytic Anemia (AIHA) | Recruiting | Early Phase 1 | To evaluate the safety, pharmacokinetics, and pharmacodynamics of Arnovie101 in B-cell-mediated autoimmune diseases. | NO |
| Functional reprogramming | NCT07617740 | IASO207 Injection | Refractory Systemic Lupus Erythematosus | Recruiting | Early Phase 1 | To evaluate the safety, tolerability, and preliminary efficacy of IASO207 injection in active refractory systemic lupus erythematosus. | NO |
| Functional reprogramming | NCT07614191 | GT801 Injection | Moderate to Severe Refractory Autoimmune Diseases | Recruiting | Early Phase 1 | To evaluate the safety, efficacy, and cellular pharmacokinetics of GT801 injection in moderate to severe refractory autoimmune diseases. | NO |
| Functional reprogramming | NCT07586267 | QT-219CX | Autoimmune Diseases\|Systemic Lupus Erythematosus (SLE)\|Multi-Drug Resistant Nephrotic Syndrome\|IgA Nephropathy (IgAN)\|Systemic Sclerosis (SSc)\|ANCA Associated Systemic Vasculitis | Recruiting | Early Phase 1 | To evaluate the safety, tolerability, and preliminary efficacy of QT-219CX in B-cell-related autoimmune diseases. | NO |
| Functional reprogramming | NCT07558850 | anti-CD19/BCMA-UCAR-T cells | Rheumatoid Arthritis\|Dermatomyositis\|Systemic Lupus Erythematosus\|Systemic Sclerosis | Recruiting | NA | To evaluate the safety and efficacy of CD19 and BCMA-targeted UCAR-T cells in refractory autoimmune diseases. | NO |
| Functional reprogramming | NCT07528105 | Allogeneic CD7-Targeted CAR-T Cell Injection | T1DM - Type 1 Diabetes Mellitus | Recruiting | Early Phase 1 | To evaluate the safety, preliminary efficacy, and cellular kinetics of allogeneic CD7-targeted CAR-T cell injection in early-stage type 1 diabetes mellitus. | NO |
| Functional reprogramming | NCT07523542 | Autologous anti-CD19 CAR-T cells / Autologous anti-BCMA CAR-T cells | Refractory Systemic Lupus Erythematosus\|Lupus Nephritis | Recruiting | Phase 1/2 | To evaluate the safety, recommended dose, and week 24 remission rate of biomarker-guided autologous CAR-T therapy targeting CD19 or BCMA in refractory systemic lupus erythematosus. | NO |
| Functional reprogramming | NCT07526493 | Universal CD19 CAR-γδT Cell Injection | Multiple Sclerosis (MS)\|Neuromyelitis Optica Spectrum Disorder (NMOSD)\|Autoimmune Encephalitis (AE)\|Chronic Inflammatory Demyelinating Polyneuropathy (CIDP)\|Myasthenia Gravis (MG)\|Anti-Myelin Oligodendrocyte Glycoprotein Immunoglobulin G Antibody-Associated Disease (MOGAD)\|Idiopathic Inflammatory Myopathies (IIM) | Recruiting | Phase 1 | To evaluate the safety and pharmacodynamics of universal CD19 CAR γδ T-cell injection in relapsed or refractory antibody-mediated autoimmune neurological diseases. | NO |
| Functional reprogramming | NCT07507201 | QT-219C | Autoimmune Diseases\|Systemic Lupus Erythematosus (SLE)\|Multi-Drug Resistant Nephrotic Syndrome\|IgA Nephropathy (IgAN)\|Systemic Sclerosis (SSc)\|ANCA Associated Systemic Vasculitis | Recruiting | Early Phase 1 | To evaluate the safety, tolerability, and preliminary efficacy of QT-219C in B-cell-related autoimmune diseases. | NO |
| Functional reprogramming | NCT07490041 | KN5601 | Rheumatic Diseases\|Pediatric Rheumatological Condition (i.e., Arthritis, SLE, Kawasaki's Disease)\|Systemic Lupus Erythematosus (SLE)\|Connective Tissue Disease-associated Interstitial Lung Disease | Recruiting | Early Phase 1 | To evaluate the safety and efficacy of KN5601 in relapsed or refractory pediatric rheumatic diseases. | NO |
| Functional reprogramming | NCT07473154 | QEL-005 | Diffuse Cutaneous Systemic Sclerosis\|Rheumatoid Arthritis (RA)\|Systemic Sclerosis (SSc)\|Autoimmune Rheumatologic Disease | Recruiting | Phase 1/2 | To evaluate the safety, tolerability, preliminary efficacy, and biological activity of QEL-005 in diffuse cutaneous systemic sclerosis and difficult-to-treat rheumatoid arthritis. | NO |
| Functional reprogramming | NCT07470151 | EVM18001 Injection | Systemic Lupus Erythematosus (SLE)\|Scleroderma\|Myasthenia Gravis (MG) | Recruiting | NA | To evaluate the safety, tolerability, recommended dose, PK, PD, preliminary efficacy, relevant biomarkers, and immunogenicity of EVM18001 injection in active refractory autoimmune diseases. | NO |
| Functional reprogramming | NCT07448298 | AFN50 injection | Relapsed/Refractory Autoimmune Diseases | Recruiting | Early Phase 1 | To evaluate the safety, tolerability, and preliminary efficacy of AFN50 injection in adults with B-cell-mediated relapsed or refractory autoimmune diseases. | NO |
| Functional reprogramming | NCT07441525 | RD06-05 | Autoimmune Hemolytic Anemia\|Primary Immune Thrombocytopenic Purpura\|Evans Syndrome | Recruiting | Early Phase 1 | To evaluate the safety, tolerability, pharmacokinetics, pharmacodynamics, and efficacy of RD06-05 in autoantibody-mediated benign autoimmune hematologic diseases. | NO |
| Functional reprogramming | NCT07444307 | QT-019B Cell Injection | Systemic Lupus Erythematosus (SLE) | Recruiting | Phase 1 | To evaluate the safety and tolerability of QT-019B cell injection in refractory systemic lupus erythematosus and to determine the recommended dose. | NO |
| Functional reprogramming | NCT07413341 | TI-0032-III injection | Systemic Lupus Erythematosus (SLE)\|Sjogren's Syndrome (SS)\|Systemic Sclerosis (SSc)\|Inflammatory Myopathy\|ANCA Associated Systemic Vasculitis | Recruiting | Phase 1 | To evaluate the safety and efficacy of TI-0032-III injection in relapsed and refractory autoimmune diseases. | NO |
| Functional reprogramming | NCT07408336 | AFN50 injection | Autoimmune Diseases | Recruiting | Early Phase 1 | To evaluate the safety, tolerability, and primary efficacy of AFN50 injection in autoimmune diseases, mainly relapsing and refractory systemic lupus erythematosus. | NO |
| Functional reprogramming | NCT07403097 | PTOC1 | Systemic Lupus Erythematosus | Recruiting | Phase 1 | To evaluate the safety and efficacy of PTOC1 in refractory systemic lupus erythematosus. | NO |
| Functional reprogramming | NCT07361094 | Autologous CD19-BCMA Dual-Target CAR T-Cell Therapy | Relapsed/Refractory Systemic Lupus Erythematosus\|Relapsed/Refractory Systemic Sclerosis\|Relapsed/Refractory Idiopathic Inflammatory Myopathies\| Relapsed/Refractory Sjögren's Syndrome\|Relapsed/Refractory Autoimmune Hemolytic Anemia\|Relapsed/Refractory Multiple Sclerosis | Recruiting | Phase 1 | To evaluate the safety and efficacy of autologous CD19 and BCMA dual-target CAR-T cell therapy in relapsed or refractory autoimmune diseases. | NO |
| Functional reprogramming | NCT07339332 | CT1195E CAR-T cells Injection | Systemic Lupus Erythematosus (SLE) | Recruiting | Early Phase 1 | To evaluate the safety, efficacy, and cellular metabolic dynamics of CT1195E CAR-T cell injection in moderate to severe refractory SLE. | NO |
| Functional reprogramming | NCT07339540 | V001-BCMA | Recurrent or Refractory Systemic Lupus Erythematosus\|Recurrent or Refractory IgG4 Related Diseases\|Recurrent or Refractory Systemic Sclerosis\|Recurrent or Refractory Idiopathic Inflammatory Myopathy\|Recurrent or Refractory ANCA Associated Vasculitis | Recruiting | Early Phase 1 | To evaluate the safety, tolerability, efficacy, pharmacokinetics, and pharmacodynamics of V001-BCMA in recurrent or refractory autoimmune diseases. | NO |
| Functional reprogramming | NCT07337785 | RD06-05 | Relapsing or Refractory Multiple Sclerosis (MS)\|Myasthenia Gravis (MG)\|Chronic Inflammatory Demyelinating Polyneuropathy (CIDP)\|Autoimmune Encephalitis (AE) | Recruiting | Early Phase 1 | To evaluate the safety, tolerability, pharmacokinetics, pharmacodynamics, and efficacy of RD06-05 in autoimmune neurological diseases. | NO |
| Functional reprogramming | NCT07331467 | CD19 CAR-T cells | Systemic Lupus Erythematosus | Recruiting | Phase 1 | To evaluate the safety and efficacy of ultra-fast autologous CD19-targeted CAR-T cells in refractory systemic lupus erythematosus. | NO |
| Functional reprogramming | NCT07333118 | GT801 Injection | Moderate to Severe Refractory Autoimmune Disease | Recruiting | Early Phase 1 | To evaluate the safety, efficacy, and cellular metabolism kinetics of GT801 injection in moderate to severe refractory autoimmune diseases. | NO |
| Functional reprogramming | NCT07333677 | HN2301 | Graves' Disease | Recruiting | Early Phase 1 | To evaluate the safety and efficacy of HN2301 in refractory Graves’ disease. | NO |
| Functional reprogramming | NCT07331272 | LUCAR-G79D T cells | Systemic Lupus Erythematosus (SLE)\|Idiopathic Inflammatory Myopathies(IIM) | Recruiting | Phase 1 | To evaluate the safety, tolerability, pharmacokinetics, pharmacodynamics, and efficacy of LUCAR-G79D T cells in relapsed or refractory SLE and relapsed or refractory IIM. | NO |
| Functional reprogramming | NCT07322718 | RXIM002 | Relapsed/Refractory B Cell-Mediated Autoimmune Diseases | Recruiting | Early Phase 1 | To evaluate the safety, tolerability, pharmacokinetics, pharmacodynamics, immunogenicity, and preliminary efficacy of RXIM002 in relapsed or refractory B-cell-mediated autoimmune diseases. | NO |
| Functional reprogramming | NCT07318259 | BCMA/CD70 CAR-T cells | Systemic Lupus Erythematosus | Recruiting | Phase 1 | To evaluate the safety and efficacy of ultra-fast autologous BCMA and CD70-targeted CAR-T cells in refractory systemic lupus erythematosus. | NO |
| Functional reprogramming | NCT07315087 | QT-019C | SLE - Systemic Lupus Erythematosus\|SSc-Systemic Sclerosis\|IIM- Idiopathic Inflammatory Myopathies\|ANCA Associated Vasculitis (AAV)\|Connective Tissue Disease-Associated Thrombocytopenia\|SLE-ITP | Recruiting | Phase 1 | To evaluate the safety and preliminary efficacy of universal allogeneic CAR-T therapy targeting CD19 and BCMA in relapsed or refractory autoimmune diseases. | NO |
| Functional reprogramming | NCT07303790 | GC012F CAR-T Cell Injection | Multiple Sclerosis | Recruiting | Early Phase 1 | To evaluate the tolerability, safety, efficacy, PK, and PD of GC012F CAR-T cell injection in patients with multiple sclerosis. | NO |
| Functional reprogramming | NCT07304154 | KITE-363 | Chronic Inflammatory Demyelinating Polyneuropathy\|Myasthenia Gravis\|Multiple Sclerosis | Recruiting | Phase 1 | To evaluate the safety, tolerability, recommended dose, and preliminary efficacy of KITE-363 in relapsed or refractory autoimmune neurological diseases. | NO |
| Functional reprogramming | NCT07305116 | UCAR T-cell | Autoimmune Diseases\|Systemic Lupus Erthematosus (SLE)\|Multi-Drug Resistant Nephrotic Syndrome\|IgA Nephropathy (IgAN)\|Systemic Sclerosis (SSc)\|ANCA Associated Systemic Vasculitis | Recruiting | Phase 1 | To evaluate the safety and preliminary efficacy of UCAR T-cell therapy in B-cell-mediated autoimmune disease. | NO |
| Functional reprogramming | NCT07295847 | AZD0120 | Systemic Sclerosis\|Idiopathic Inflammatory Myopathies\|Rheumatoid Arthritis | Recruiting | Phase 1 | To evaluate the safety and tolerability of AZD0120 in adults with systemic sclerosis, idiopathic inflammatory myopathies, or difficult-to-treat rheumatoid arthritis, and to determine the recommended Phase 2 dose. | NO |
| Functional reprogramming | NCT07283315 | anti-CD19/BCMA CAR NK cells (KN5601-K) | Paediatric B Cell-related Autoimmune Diseases\|Autoimmune Diseases\|CAR | Recruiting | Early Phase 1 | To evaluate the safety and efficacy of anti-CD19 and anti-BCMA CAR NK cells, KN5601-K, in pediatric B-cell-related autoimmune diseases. | NO |
| Functional reprogramming | NCT07274059 | allogeneic umbilical cord blood-derived CAR-T targeting CD19 and BCMA | SLE - Systemic Lupus Erythematosus | Recruiting | Early Phase 1 | To evaluate the safety and efficacy of allogeneic umbilical cord blood-derived CAR-T cells targeting CD19 and BCMA in refractory systemic lupus erythematosus. | NO |
| Functional reprogramming | NCT07275736 | CNCT19 cell injection | Neurological Autoimmune Diseases | Recruiting | Early Phase 1 | To evaluate the safety, tolerability, and preliminary efficacy of CNCT19 cell injection in relapsed or refractory autoimmune neurological diseases. | NO |
| Functional reprogramming | NCT07266181 | CD19 CAR-T cell therapy | Refractory Membranous Nephropathy | Recruiting | Phase 1 | To preliminarily evaluate the safety and efficacy of CD19 CAR-T cell therapy in refractory membranous nephropathy. | NO |
| Functional reprogramming | NCT07261345 | RD06-05 | Graves' Disease | Recruiting | Early Phase 1 | To evaluate the safety and efficacy of RD06-05 in refractory Graves’ disease. | NO |
| Functional reprogramming | NCT07246096 | KN3601 | Autoimmune Diseases\|Systemic Lupus Erythematosus\|Systemic Sclerosis\|Primary Sjögren Syndrome\|Rheumatoid Arthritis\|Anti-Neutrophil Cytoplasmic Antibody-Associated Vasculitis | Recruiting | Early Phase 1 | To evaluate the safety and efficacy of KN3601 in relapsed or refractory autoimmune diseases. | NO |
| Functional reprogramming | NCT07236762 | YTS109 cell | Systemic Lupus Erythematosus\|Lupus Nephritis (LN)\|Sjogren's Syndrome\|Inflammatory Myopathy\|Anti-Neutrophil Cytoplasmic Antibody-Associated Vasculitis\|Antiphospholipid Syndrome | Recruiting | Phase 1 | To evaluate the safety and efficacy of YTS109 cells in relapsing or refractory autoimmune diseases. | NO |
| Functional reprogramming | NCT07236801 | YTS109 cell | Systemic Lupus Erythematosus (SLE)\|Systemic Sclerosis\|Sjogren's Syndrome (SS)\|Inflammatory Myopathy\|Anti-Neutrophil Cytoplasmic Antibody-Associated Vasculitis\|Antiphospholipid Syndrome | Recruiting | Phase 1 | To evaluate the safety and efficacy of YTS109 cells in relapsing or refractory autoimmune diseases. | NO |
| Functional reprogramming | NCT07233642 | CD19 CAR-T cells | Systemic Lupus Erythematosus | Recruiting | Phase 1 | To evaluate the safety and efficacy of ultra-fast autologous CD19-targeted CAR-T cells in refractory systemic lupus erythematosus. | NO |
| Functional reprogramming | NCT07224373 | AZD0120 | Multiple Sclerosis | Recruiting | Phase 1 | To evaluate the safety and tolerability of AZD0120 in adults with refractory relapsing or progressive multiple sclerosis, and to determine the recommended Phase 2 dose. | NO |
| Functional reprogramming | NCT07203404 | RD06-05 CART Cell Injection | SLE\|LN\|ANCA Associated Vasculitis\|ANCA-Associated Glomerulonephritis\|Anti-GBM Disease\|MN\|SSc\|IIM | Recruiting | Early Phase 1 | To evaluate the safety, tolerability, PK, PD, and efficacy of anti-CD19 and anti-BCMA universal CAR-T cell therapy RD06-05 in patients with autoimmune diseases. | NO |
| Functional reprogramming | NCT07193667 | CD19 CAR-T cells | Relapsed/Refractory Autoimmune Diseases | Recruiting | NA | To evaluate the safety and efficacy of CD19 CAR-T cells in relapsed or refractory autoimmune diseases. | NO |
| Functional reprogramming | NCT07184450 | BCMA/CD70-targeted CAR-T | Juvenile Dermatomyositis (JDM)\|Polyarticular Juvenile Idiopathic Arthritis\|Systemic Sclerosis (SSc)\|Primary Sjogren's Syndrome | Recruiting | Phase 1 | To evaluate the efficacy and safety of BCMA and CD70-targeted CAR-T therapy in refractory pediatric rheumatic diseases. | NO |
| Functional reprogramming | NCT07178431 | MB-CART2019.1 | Multiple Sclerosis\|CAR T Cell Therapy | Recruiting | Phase 1/2 | To evaluate the feasibility, safety, and preliminary efficacy of MB-CART2019.1 in active refractory primary and secondary progressive multiple sclerosis. | NO |
| Functional reprogramming | NCT07174843 | BZE2204 | Idiopathic Inflammatory Myopathies(IIM)\|Immune Thrombocytopenia(ITP)\|Systemic Lupus Erythematosus(SLE) | Recruiting | Early Phase 1 | To evaluate the safety, tolerability, and preliminary efficacy of BZE2204 in relapsed or refractory active autoimmune diseases, and to determine the maximum tolerated dose or recommended dose. | NO |
| Functional reprogramming | NCT07167537 | OL-CD19-GDT | Systemic Sclerosis (SSc)\|Primary Sjogren's Syndrome | Recruiting | Phase 1 | To evaluate the safety, tolerability, pharmacokinetics, pharmacodynamics, and preliminary efficacy of OL-CD19-GDT in relapsed or refractory autoimmune diseases. | NO |
| Functional reprogramming | NCT07155369 | UCAR-T cells | Systemic Lupus Erythematosus\|Systemic Sclerosis | Active, not recruiting | NA | To evaluate the safety and efficacy of UCAR T-cell therapy targeting CD19 and BCMA in relapsed or refractory autoimmune diseases. | NO |
| Functional reprogramming | NCT07148791 | anti-BCMA-CD19 CAR-T cells | IgG4 Related Disease\|B-cell Mediated Autoimmune Disorders | Recruiting | Phase 2 | To evaluate the safety, feasibility, and preliminary efficacy of anti-BCMA and anti-CD19 CAR-T cells in relapsed or refractory IgG4-related disease. | NO |
| Functional reprogramming | NCT07143617 | STR-P004 | Autoimmune Diseases | Recruiting | NA | To evaluate the safety, tolerability, PK, and preliminary efficacy of STR-P004 in relapsed or refractory autoimmune diseases. | NO |
| Functional reprogramming | NCT07144462 | NEUK203-215 Injection | Autoimmune | Recruiting | Early Phase 1 | To evaluate the safety, tolerability, and preliminary efficacy of NEUK203-215 injection in adults with relapsed or refractory autoimmune diseases. | NO |
| Functional reprogramming | NCT07142161 | RD13-02 cell infusion | Type 1 Diabetes | Active, not recruiting | Early Phase 1 | To evaluate the safety, tolerability, pharmacokinetics, pharmacodynamics, efficacy, and cellular kinetics of RD13-02 cell infusion in type 1 diabetes. | NO |
| Functional reprogramming | NCT07139743 | Obecabtagene autoleucel | Progressive Multiple Sclerosis | Recruiting | Phase 1 | To evaluate the safety, tolerability, and preliminary efficacy of obecabtagene autoleucel in refractory progressive multiple sclerosis. | NO |
| Functional reprogramming | NCT07132112 | GT719 Injection | Auto Immune Disease | Recruiting | Early Phase 1 | To evaluate the safety, efficacy, and cellular pharmacokinetics of GT719 injection in moderate to severe refractory autoimmune disease. | NO |
| Functional reprogramming | NCT07129642 | Allogeneic anti-CD19 CAR-T | Graves Disease | Recruiting | Early Phase 1 | To evaluate the efficacy and safety of allogeneic anti-CD19 CAR-T therapy in refractory Graves’ disease. | NO |
| Functional reprogramming | NCT07122076 | GT719 Injection | Autoimmune Diseases | Recruiting | Early Phase 1 | To evaluate the safety, efficacy, and cellular pharmacokinetics of GT719 injection in moderate to severe refractory autoimmune diseases. | NO |
| Functional reprogramming | NCT07123519 | YTS109 cell | Systemic Lupus Erythematosus (SLE)\|Lupus Nephritis (LN)\|Sjogren's Syndrome (SS)\|Inflammatory Myopathy\|Anti-Neutrophil Cytoplasmic Antibody-Associated Vasculitis\|Antiphospholipid Syndrome (APS) | Recruiting | Phase 1 | To evaluate the safety, efficacy, PK, and PD of YTS109 cells in relapsed or refractory autoimmune diseases. | NO |
| Functional reprogramming | NCT07121777 | Anti-CD19/BCMA CAR gene vector injection | Autoimmune Diseases | Recruiting | NA | To evaluate the safety and efficacy of anti-CD19 and anti-BCMA CAR gene vector injection in refractory autoimmune diseases. | NO |
| Functional reprogramming | NCT07115745 | BMS-986515 | Refractory Autoimmune Diseases | Recruiting | Phase 1 | To evaluate the safety, tolerability, optimal dose, and preliminary efficacy of BMS-986515 in severe refractory autoimmune diseases. | NO |
| Functional reprogramming | NCT07104721 | YTS109 cell | Systemic Lupus Erythematosus (SLE)\|Lupus Nephritis (LN)\|Systemic Sclerosis (SSc)\|Inflammatory Myopathy\|Anti-Neutrophil Cytoplasmic Antibody-Associated Vasculitis\|Antiphospholipid Syndrome (APS) | Recruiting | Phase 1 | To evaluate the safety, efficacy, PK, and PD of YTS109 cells in relapsed or refractory autoimmune diseases. | NO |
| Functional reprogramming | NCT07100873 | ADI-001 | Rheumatoid Arthritis (RA) | Recruiting | Phase 1 | To compare ADI-001 in treatment-refractory rheumatoid arthritis across different lymphodepletion regimens. | NO |
| Functional reprogramming | NCT07095075 | LUCAR-DKS1 NK cells | Relapsed/Refractory Autoimmune Diseases | Recruiting | Early Phase 1 | To evaluate the safety, tolerability, pharmacokinetics, pharmacodynamics, and efficacy of LUCAR-DKS1 in subjects with relapsed or refractory autoimmune diseases. | NO |
| Functional reprogramming | NCT07088068 | Teplizumab | Type 1 Diabetes Mellitus | Recruiting | Phase 3 | To evaluate the efficacy and safety of teplizumab versus placebo in patients aged 1 to 25 years with recently diagnosed stage 3 type 1 diabetes, and to assess changes in glycemic control and mealtime insulin independence over 52 weeks. | NO |
| Functional reprogramming | NCT07089121 | Descartes-08 | Childhood-onset Systemic Lupus Erythematous\|ANCA-Associated Vasculitis (AAV)\|Juvenile Myasthenia Gravis\|Juvenile Dermatomyositis | Recruiting | Phase 1/2 | To evaluate the safety, tolerability, and efficacy of Descartes-08 in pediatric, adolescent, and young adult patients with autoimmune diseases. | NO |
| Functional reprogramming | NCT07085104 | ALLO-329 | Systemic Lupus Erythematosus (With and Without Nephritis)\|Idiopathic Inflammatory Myopathy\|Systemic Sclerosis | Recruiting | Phase 1 | To evaluate the safety, tolerability, and preliminary efficacy of ALLO-329 in autoimmune disease, and to determine the recommended Phase 2 regimen. | NO |
| Functional reprogramming | NCT07085676 | HBI0101 CART | Systemic Sclerosis (SSc)\|Idiopathic Inflammatory Myopathy (IIM)\|Rheumatoid Arthritis (RA)\|Systemic Lupus Erythematosus (SLE)\|Multiple Sclerosis (MS) Primary Progressive\|Multiple Sclerosis (MS) Secondary Progressive\|Neuromyelitis Optica Spectrum Disorder (NMOSD)\|Myasthenia Gravis (MG)\|Antiphospholipid Antibody Syndrome | Recruiting | Phase 1 | To evaluate the safety of HBI0101 CAR-T therapy in B-cell-mediated refractory autoimmune diseases and to determine the maximum safely administered dose. | NO |
| Functional reprogramming | NCT07058298 | GC012F injection | Generalized Myasthenia Gravis (gMG) | Recruiting | Early Phase 1 | To evaluate the safety, tolerability, preliminary clinical efficacy, pharmacokinetics, pharmacodynamics, and immunogenicity of GC012F injection in refractory gMG. | NO |
| Functional reprogramming | NCT07059169 | JY231 | Autoimmune Diseases | Recruiting | NA | To evaluate the safety, tolerability, and preliminary efficacy of JY231 in refractory autoimmune diseases. | NO |
| Functional reprogramming | NCT07053800 | Obecabtagene autoleucel | Lupus Nephritis | Recruiting | Phase 2 | To evaluate the safety and efficacy of obecabtagene autoleucel in severe refractory systemic lupus erythematosus with active lupus nephritis. | NO |
| Functional reprogramming | NCT07051564 | CNK-UT009 cell injection | Type 1 Diabetes (T1D) | Recruiting | NA | To evaluate the safety, tolerability, maximum tolerated dose, preliminary efficacy, pharmacokinetics, and immunogenicity of CNK-UT009 cell injection in type 1 diabetes, as well as its effects on peripheral blood immune cells and serum cytokines. | NO |
| Functional reprogramming | NCT07052565 | ECAR01 | Autoimmune Diseases | Recruiting | NA | To evaluate the safety and efficacy of ECAR01 in treating refractory autoimmune diseases. | NO |
| Functional reprogramming | NCT07048197 | Rapcabtagene autoleucel | Rheumatoid Arthritis (RA) and Sjögren's Disease (SjD) | Recruiting | Phase 1/2 | To assess the safety, cellular kinetics, and exploratory efficacy of rapcabtagene autoleucel in patients with rheumatoid arthritis or Sjögren’s disease. | NO |
| Functional reprogramming | NCT07031713 | CT1192 cells infusion | Systemic Lupus Erythematosus (SLE) | Recruiting | Phase 1 | To evaluate the safety, efficacy, and cellular metabolic dynamics of CT1192 cell infusion in patients with moderate to severe refractory SLE. | NO |
| Functional reprogramming | NCT07022197 | BAFF-R CART | Chronic Inflammatory Demyelinating Polyradiculoneuropathy\|NMO Spectrum Disorder\|Myasthenia Gravis\|Idiopathic Inflammatory Myopathies | Recruiting | Phase 1/2 | To evaluate the safety, tolerability, and preliminary efficacy of BAFF-R CAR T-cell therapy in refractory neuroimmune diseases. | NO |
| Functional reprogramming | NCT07021209 | GT719 Injection | Neurological Autoimmune Diseases | Recruiting | Phase 1 | To evaluate the safety, cell kinetics, and preliminary efficacy of GT719 Injection in recurrent or refractory antibody-mediated neurological immune diseases. | NO |
| Functional reprogramming | NCT07015983 | CC-97540 | Lupus Erythematosus, Systemic\|Lupus Nephritis | Recruiting | Phase 2 | To evaluate the efficacy, safety, and drug levels of CC-97540 in patients with active systemic lupus erythematosus, including those with lupus nephritis. | NO |
| Functional reprogramming | NCT07013110 | dnaJP1 | Rheumatoid Arthritis (RA)\|Rheumatology | Recruiting | Phase 2 | To evaluate the efficacy of dnaJP1 combined with hydroxychloroquine in moderate-to-severe active RA and investigate its mechanisms in inducing immune tolerance. | NO |
| Functional reprogramming | NCT07008378 | P-CD19CD20-ALLO1 Cells | Multiple Sclerosis | Active, not recruiting | Phase 1 | To assess the safety, tolerability, cellular kinetics, and pharmacodynamics of P-CD19CD20-ALLO1 cells in the treatment of multiple sclerosis. | NO |
| Functional reprogramming | NCT07001956 | UTAA91 injection | Rheumatoid Arthritis (RA) | Recruiting | Early Phase 1 | To evaluate the safety of UTAA91 injection in patients with refractory moderate-to-severe active rheumatoid arthritis. | NO |
| Functional reprogramming | NCT06993493 | CD19 CART | Autoimmune Disease | Recruiting | NA | To evaluate the safety and efficacy of autologous CD19 CAR-T therapy in relapsed or refractory autoimmune diseases. | NO |
| Functional reprogramming | NCT06983964 | CD19 CART | Autoimmune Disease | Recruiting | NA | To evaluate the safety and efficacy of universal CD19 CAR-T therapy in recurrent or refractory autoimmune diseases. | NO |
| Functional reprogramming | NCT06980597 | OL-108 | Systemic Lupus Erythematosus (SLE)\|Idiopathic Inflammatory Myopathy (IIM)\|Systemic Sclerosis (SSc)\|ANCA Associated Vasculitis (AAV) | Recruiting | Phase 1 | To evaluate the safety, tolerability, pharmacokinetics, pharmacodynamics, and preliminary efficacy of OL-108 in relapsed or refractory autoimmune diseases. | NO |
| Functional reprogramming | NCT06978647 | YTS109 cell | Systemic Lupus Erythematosus (SLE)\|Systemic Sclerosis (SSc)\|Inflammatory Myopathy\|Anti-Neutrophil Cytoplasmic Antibody-Associated Vasculitis\|Antiphospholipid Syndrome (APS)\|Rheumatoid Arthritis (RA)\|IgG4-Related Diseases\|Sjogren's Syndrome | Recruiting | Phase 1 | To evaluate the safety, preliminary efficacy, pharmacokinetics, and pharmacodynamics of YTS109 cells in relapsed or refractory autoimmune diseases. | NO |
| Functional reprogramming | NCT06975787 | Vonsetamig; Odronextamab | Lupus Nephritis (LN) | Recruiting | Phase 1 | To evaluate the safety, tolerability, serum drug concentrations, and immunogenicity of bispecific antibodies in patients with lupus nephritis. | NO |
| Functional reprogramming | NCT06973356 | LCAR1901 | Immune Thrombocytopenia (ITP) | Recruiting | NA | To evaluate the safety and efficacy of LCAR1901 in relapsed and refractory ITP. | NO |
| Functional reprogramming | NCT06946485 | Universal anti-CD70 CAR-T (CHT101) | Systemic Lupus Erythematosus (SLE) | Recruiting | Early Phase 1 | To evaluate the safety and efficacy of universal anti-CD70 CAR-T cells (CHT101) in relapsed and refractory systemic lupus erythematosus. | NO |
| Functional reprogramming | NCT06947460 | CD19-BCMA CAR-T cells infusion | Refractory Lupus Nephritis\|Systemic Sclerosis\|Primary Sjogren's Syndrome Combined With Pulmonary Hypertension | Recruiting | Phase 1/2 | To evaluate the safety of CD19-BCMA CAR-T cell infusion in patients with refractory lupus nephritis, systemic sclerosis, or primary Sjögren’s syndrome with pulmonary hypertension. | NO |
| Functional reprogramming | NCT06947473 | umbilical cord blood CD19-BCMA CAR-T cells infusion | Refractory Lupus Nephritis\|Systemic Sclerosis\|Primary Sjogren's Syndrome Combined With Pulmonary Hypertension | Recruiting | Phase 1/2 | To evaluate the safety of umbilical cord blood-derived CD19-BCMA CAR-T cell infusion in patients with refractory lupus nephritis, systemic sclerosis, or primary Sjögren’s syndrome complicated by pulmonary arterial hypertension. | NO |
| Functional reprogramming | NCT06943937 | YTS109 cell injection | Lupus Nephritis (LN)\|Immune Thrombocytopenia (ITP) | Recruiting | Phase 1 | To evaluate the safety, efficacy, and pharmacokinetic and pharmacodynamic characteristics of YTS109 cell injection in the treatment of refractory SLE-associated lupus nephritis and SLE-associated immune thrombocytopenia. | NO |
| Functional reprogramming | NCT06941129 | UCAR T-cell | Systemic Lupus Erythematosus\|Systemic Sclerosis (SSc)\|Inflammatory Myopathy\|ANCA-Associated Vasculitis (AAV)\|Connective Tissue Disease-Associated Thrombocytopenia\|SLE-ITP | Recruiting | Phase 1 | To evaluate the safety and preliminary efficacy of UCAR T-cell therapy in patients with relapsed or refractory autoimmune diseases. | NO |
| Functional reprogramming | NCT06939166 | UCAR T-cell | Neuromyelitis Optica Spectrum Disorders\|Myasthenia Gravis\|Multiple Sclerosis\|Chronic Inflammatory Demyelinating Polyradiculoneuropathy\|Autoimmune Encephalitis | Recruiting | Early Phase 1 | To evaluate the safety and preliminary efficacy of UCAR T-cell therapy in patients with relapsed/refractory neurological autoimmune diseases. | NO |
| Functional reprogramming | NCT06934447 | anti-BCMA/CD70-CAR-T cells | Childhood-onset Systemic Lupus Erythematosus | Recruiting | Phase 1 | To evaluate the safety and efficacy of anti-BCMA/CD70 CAR-T cells in the treatment of refractory childhood-onset systemic lupus erythematosus. | NO |
| Functional reprogramming | NCT06925542 | CTX112 | SLE (Systemic Lupus)\|Lupus Erythematosus, Systemic\|Lupus Nephritis\|Systemic Sclerosis\|Inflammatory Myopathy, Idiopathic\|Myositis\|Diffuse Cutaneous Systemic Sclerosis | Recruiting | Phase 1 | To evaluate the safety and preliminary efficacy of CTX112 in adults with refractory autoimmune diseases. | NO |
| Functional reprogramming | NCT06919354 | GNTI-122 | Type 1 Diabetes (T1D)\|Type 1 Diabetes Mellitus | Recruiting | Phase 1 | To evaluate the safety, tolerability, cellular kinetics, and changes in C-peptide biomarkers of GNTI-122 in adults with recently diagnosed type 1 diabetes. | NO |
| Functional reprogramming | NCT06920433 | Universal allogeneic CD19/BCMA CAR T-cells | Systemic Lupus Erythematosus | Recruiting | Early Phase 1 | To evaluate the safety and efficacy of universal allogeneic CD19/BCMA CAR T cells in the treatment of relapsed/refractory systemic lupus erythematosus. | NO |
| Functional reprogramming | NCT06904729 | CAR-T cells | Lupus Nephritis | Recruiting | Phase 3 | To evaluate the safety and potential efficacy of CAR-T cell therapy in children with refractory/recurrent lupus nephritis | NO |
| Functional reprogramming | NCT06902844 | Equecabtagene Autoleucel Injection | Systemic Lupus Erythematosus (SLE)\|Lupus Nephritis (LN) | Recruiting | NA | To evaluate the efficacy and safety of Equecabtagene Autoleucel Injection in patients with relapsed/refractory Systemic Lupus Erythematosus | NO |
| Functional reprogramming | NCT06897930 | AZD0120 | Lupus Erythematosus, Systemic | Recruiting | Phase 1/2 | To evaluate the safety, tolerability, and efficacy of AZD0120 in adult participants with refractory Systemic Lupus Erythematosus. | NO |
| Functional reprogramming | NCT06892145 | MC-1-50 | Systemic Lupus Erythematosus (SLE) | Recruiting | Phase 1 | To evaluate the safety, tolerability, cytodynamic characteristics, preliminary efficacy, and applicable Phase 2 dose regimen of MC-1-50 in refractory adult SLE. | NO |
| Functional reprogramming | NCT06869278 | LCAR-AIO T cells | Multiple Sclerosis (MS)\|Neuromyelitis Optica Spectrum Disease (NMOSD)\|Anti-Myelin Oligodendrocyte Glycoprotein-IgG Associated Disorders (MOGAD)\|Myasthenia Gravis | Recruiting | Phase 1 | To evaluate the safety, tolerability, pharmacokinetics, pharmacodynamics, and efficacy profiles of LCAR-AIO CAR-T cells in subjects with relapsed/refractory neurological autoimmune diseases | NO |
| Functional reprogramming | NCT06868290 | Rapcabtagene autoleucel | ANCA Associated Vasculitis (AAV) | Recruiting | Phase 2 | To evaluate the efficacy and safety of rapcabtagene autoleucel versus comparator in participants with severe active granulomatosis with polyangiitis or microscopic polyangiitis | NO |
| Functional reprogramming | NCT06839976 | CART19 | Systemic Lupus Erythematosus (SLE)\|Lupus Nephritis (LN) | Recruiting | Phase 1/2 | To evaluate the safety and efficacy of CART19 in children and young adults with refractory systemic lupus erythematosus, including lupus nephritis and non-renal SLE | NO |
| Functional reprogramming | NCT06826430 | Inaticabtagene Autoleucel Injection | Autoimmune Thrombocytopenia | Recruiting | Phase 1 | To evaluate the safety and tolerability of Inaticabtagene Autoleucel Injection in refractory systemic lupus erythematosus-related immune thrombocytopenia and determine the Phase 2 recommended dose | NO |
| Functional reprogramming | NCT06828042 | QH103 | Systemic Lupus Erythematosus\|Systemic Sclerosis (SSc)\|Sjogren Syndrome\|ANCA Associated Vasculitis (AAV)\|Inflammatory Myopathies\|Antiphospholipid Syndrome | Recruiting | Phase 1/2 | To evaluate the safety and efficacy of universal CD19-targeting CAR-γδ T cells in refractory autoimmune diseases | NO |
| Functional reprogramming | NCT06822881 | CT1190B cells infusion | Systemic Lupus Erythematosus (SLE)\|Systemic Sclerosis (SSc) | Recruiting | Phase 1 | To investigate the safety, efficacy, and cellular metabolism of CT1190B CAR-T Cell Therapy in patients with moderate to severe refractory Systemic Lupus Erythematosus or refractory/progressive Systemic Sclerosis. | NO |
| Functional reprogramming | NCT06799247 | Descartes-08 | Myasthaenia Gravis | Recruiting | Phase 3 | To evaluate the safety, tolerability, and efficacy of Descartes-08 in patients with acetylcholine receptor autoantibody-positive generalized Myasthenia Gravis | NO |
| Functional reprogramming | NCT06797024 | JY231 Injection | Autoimmune Diseases of the Nervous System | Recruiting | NA | To evaluate the safety, tolerability, and preliminary efficacy of JY231 Injection in patients with relapsed/refractory neurologic immune disorders. | NO |
| Functional reprogramming | NCT06792799 | KN5601 | Autoimmune Diseases\|Systemic Lupus Erythematosus\|Multi-Drug Resistant Nephrotic Syndrome\|IgAN - IgA Nephropathy | Recruiting | Early Phase 1 | To evaluate the efficacy and safety of anti-CD19/BCMA CAR-NK cells in children with B cell-mediated autoimmune diseases | NO |
| Functional reprogramming | NCT06794008 | BCMA-CD19 CAR-T therapy | Systemic Lupus Erythematosus\|Inflammatory Myopathy\|Systemic Sclerosis (SSc)\|ANCA-associated Vasculitis\|IgG4-Related Diseases\|Antiphospholipid Syndrome\|Acquired Thrombotic Thrombocytopenic Purpura\|Behcet Disease\|Sjogren Syndrome | Recruiting | Phase 2 | To evaluate the efficacy and safety of BCMA/CD19 CAR-modified T cells in the treatment of multiple refractory autoimmune diseases | NO |
| Functional reprogramming | NCT06791291 | Teplizumab | Type 1 Diabetes Mellitus | Recruiting | Phase 2 | To assess the efficacy, safety, tolerability, pharmacokinetics, pharmacodynamics, and immunogenicity of a 14-day intravenous infusion regimen of teplizumab in Japanese participants with Stage 2 Type 1 Diabetes | NO |
| Functional reprogramming | NCT06787989 | BCMA-CD19 cCAR T cells | Refractory Immune Cytopenia | Recruiting | Phase 1 | To evaluate the safety and tolerability of BCMA-CD19 cCAR T cells in patients with refractory immune cytopenia associated with autoimmune disease. | NO |
| Functional reprogramming | NCT06785519 | CD19/BCMA Lupus Nephritis Targeted CAR T-cells injection | Lupus Nephritis | Recruiting | Early Phase 1 | To evaluate the safety and efficacy of CD19/BCMA CAR-T cell therapy in patients with refractory/relapsed lupus nephritis | NO |
| Functional reprogramming | NCT06783309 | CNP-103 | Type 1 Diabetes Mellitus | Recruiting | Phase 1/2 | To evaluate the safety, tolerability, pharmacodynamics, and efficacy of multiple ascending doses of CNP-103 in participants with recent onset Stage 3 Type 1 Diabetes | NO |
| Functional reprogramming | NCT06775912 | RD06-05 CART Cell Injection | SLE\|Systemic Sclerosis\|IIM\|NMOSD\|MS\|MG\|ANCA Associated Vasculitis (AAV) | Recruiting | Early Phase 1 | To evaluate the safety, tolerability, pharmacokinetics, pharmacodynamics, and efficacy of RD06-05 in patients with active SLE, SSc, AAV, IIM, NMOSD, MS, and MG | NO |
| Functional reprogramming | NCT06733610 | universal allogeneic anti-CD19/BCMA CAR T-cells | Autoimmune Hemolytic Anemia | Recruiting | Phase 1 | To evaluate the safety and preliminary efficacy of universal allogeneic anti-CD19/BCMA CAR T-cell therapy in patients with autoimmune hemolytic anemia who have failed ≥3 lines of therapy | NO |
| Functional reprogramming | NCT06733935 | NKX019 | Systemic Sclerosis\|Idiopathic Inflammatory Myopathies\|Antineutrophil Cytoplasmic Antibody-Associated Vasculitis\|Rheumatoid Arthritis | Recruiting | Phase 1/2 | To determine the safety and tolerability of NKX019 and evaluate preliminary efficacy, pharmacokinetics, pharmacodynamics, and immunogenicity in participants with autoimmune diseases | NO |
| Functional reprogramming | NCT06721962 | TRX103 | Crohn's Disease | Recruiting | Phase 1/2 | To evaluate the safety and preliminary efficacy of TRX103 in subjects with moderate to severe treatment-refractory Crohn's disease | NO |
| Functional reprogramming | NCT06711146 | Meta10-19 | Systemic Lupus Erythematosus | Recruiting | Early Phase 1 | To evaluate the safety and efficacy of metabolically armed CD19 CAR-T cells in the treatment of moderate to severe active Systemic Lupus Erythematosus. | NO |
| Functional reprogramming | NCT06710717 | CD19 CAR-T cells | Systemic Lupus Erythematosus (SLE) | Recruiting | Phase 1 | To evaluate the safety and efficacy of autologous CD19 CAR-T cell infusion in patients with severe refractory Systemic Lupus Erythematosus. | NO |
| Functional reprogramming | NCT06704269 | YTB323 | Generalized Myasthenia Gravis | Recruiting | Phase 1/2 | To assess the safety, efficacy, and cellular kinetics of YTB323 in participants with treatment-resistant generalized Myasthenia Gravis | NO |
| Functional reprogramming | NCT06691152 | CD19 Universal CAR-T cells | Systemic Lupus Erythematosus | Recruiting | Phase 1 | To assess the safety and efficacy of CD19 universal CAR-T cells in the treatment of refractory systemic lupus erythematosus | NO |
| Functional reprogramming | NCT06688331 | CD4^+^CD25^+^CD127- regulatory lymphocytes; rituximab | Presymptomatic (Stage 1) Type 1 Diabetes | Recruiting | Phase 2 | To evaluate the safety and efficacy of cell therapy based on artificially expanded CD4+CD25+CD127- regulatory lymphocytes and anti-CD20 antibody in pediatric patients with presymptomatic Type 1 Diabetes. | NO |
| Functional reprogramming | NCT06688435 | SYS6020 injection | Myasthenia Gravis | Recruiting | Phase 1 | To evaluate the safety, tolerability, recommended dose, preliminary clinical efficacy, pharmacokinetics, and immunogenicity of SYS6020 injection in patients with refractory generalized Myasthenia Gravis. | NO |
| Functional reprogramming | NCT06688799 | CD19 CAR-T cells | Autoimmune Diseases | Recruiting | Phase 1/2 | To evaluate the safety and efficacy of CD19 CAR T cells in the treatment of refractory autoimmune diseases | NO |
| Functional reprogramming | NCT06685042 | CD19-CAR_Lenti | Systemic Lupus Erythematosus\|Systemic Sclerosis\|ANCA-Associated Vasculitis\|Dermatomyositis\|Polymyositis | Recruiting | Phase 1/2 | To assess the safety, feasibility, and preliminary efficacy of autologous anti-CD19 CAR T-cell therapy in subjects with refractory systemic autoimmune diseases | NO |
| Functional reprogramming | NCT06680037 | Azercabtagene zapreleucel | B-cell Mediated Autoimmune Disorders | Recruiting | Phase 1 | To determine the recommended phase 2 dose and evaluate the safety and clinical activity of azercabtagene zapreleucel in participants with B-cell mediated autoimmune disorders | NO |
| Functional reprogramming | NCT06680388 | CD19 CAR-T | Autoimmune Diseases | Recruiting | Early Phase 1 | To evaluate the safety, tolerability, and preliminary efficacy of autologous CD19 CAR-T in patients with relapsed/refractory autoimmune diseases. | NO |
| Functional reprogramming | NCT06676631 | NK010 or NK042; Rituximab | Refractory Systemic Lupus Erythematosus\|Refractory Lupus Nephritis | Recruiting | Phase 1 | To determine the safety and preliminary efficacy of NK010 or NK042 in combination with Rituximab for the treatment of refractory Systemic Lupus Erythematosus or Lupus Nephritis. | NO |
| Functional reprogramming | NCT06675422 | JY231 injection | Systemic Lupus Erythematosus (SLE) | Recruiting | Phase 1/2 | To evaluate the safety, tolerability, and efficacy of JY231 injection in patients with active systemic lupus erythematosus | NO |
| Functional reprogramming | NCT06675864 | rapcabtagene autoleucel | Progressive Multiple Sclerosis | Recruiting | Phase 1/2 | To assess safety, disease progression, and cellular kinetics following rapcabtagene autoleucel administration in participants with non-active Progressive Multiple Sclerosis | NO |
| Functional reprogramming | NCT06653556 | LCAR-AIO T cells | Systemic Lupus Erythematosus (SLE) | Recruiting | Early Phase 1 | To evaluate the safety, tolerability, pharmacokinetics, pharmacodynamics, and efficacy profiles of LCAR-AIO in adults with relapsed/refractory systemic lupus erythematosus | NO |
| Functional reprogramming | NCT06626919 | anitocabtagene autoleucel | Generalized Myasthenia Gravis | Recruiting | Phase 1 | To evaluate the safety, tolerability, and preliminary efficacy of anitocabtagene autoleucel in adult subjects with generalized myasthenia gravis. | NO |
| Functional reprogramming | NCT06617793 | rapcabtagene autoleucel | Relapsing Multiple Sclerosis | Recruiting | Phase 1/2 | To assess the safety, efficacy, and cellular kinetics of rapcabtagene autoleucel in participants with relapsing Multiple Sclerosis. | NO |
| Functional reprogramming | NCT06613490 | KN5501 | Antisynthetase Syndrome\|Rheumatoid Arthritis | Recruiting | Early Phase 1 | To evaluate the safety and efficacy of anti-CD19 CAR NK cells (KN5501), including expansion, persistence, and CD19-positive B-cell clearance, in patients with refractory antisynthetase antibody syndrome and rheumatoid arthritis | NO |
| Functional reprogramming | NCT06614270 | Anti-CD19 IL-10/IL15 CAR-NK | Systemic Sclerosis (SSc)\|ANCA Associated Vasculitis (AAV)\|Idiopathic Inflammatory Myopathy (IIM)\|Sjogren's Syndrome\|Antiphospholipid Syndrome\|Systemic Lupus Erythematosus | Recruiting | NA | To investigate the safety and efficacy of Anti-CD19 IL-10/IL15 CAR-NK cells in patients with refractory/relapsed autoimmune diseases | NO |
| Functional reprogramming | NCT06588491 | KYV-101 | Stiff-Person Syndrome | Active, not recruiting | Phase 1 | To evaluate the safety and clinical activity of KYV-101, an autologous fully human anti-CD19 CAR-T cell therapy, in subjects with treatment-refractory Stiff Person Syndrome. | NO |
| Functional reprogramming | NCT06585514 | CD19 CAR-T cells | Systemic Lupus Erythematosus (SLE)\|Lupus Nephritis (LN) | Recruiting | Phase 1/2 | To evaluate the safety, tolerability, optimal biological dose, recommended phase 2 dose, and efficacy of CD19 CAR-T cell therapy in patients with refractory systemic lupus erythematosus | NO |
| Functional reprogramming | NCT06581198 | rapcabtagene autoleucel | Lupus Erythematosus, Systemic\|Lupus Nephritis | Recruiting | Phase 2 | To evaluate the efficacy and safety of rapcabtagene autoleucel in patients with active, refractory Systemic Lupus Erythematosus or active, refractory Lupus Nephritis | NO |
| Functional reprogramming | NCT06557265 | NKX019 | Lupus Nephritis\|Primary Membranous Nephropathy | Recruiting | Phase 1/2 | To determine the safety, tolerability, recommended dose, preliminary efficacy, pharmacokinetics, and pharmacodynamics of NKX019 in participants with autoimmune diseases. | NO |
| Functional reprogramming | NCT06548607 | CD19 or CD19-BCMA CAR-T | SLE (Systemic Lupus)\|Systemic Sclerosis\|ANCA Associated Vasculitis\|Idiopathic Inflammatory Myopathies\|Sjogren's Syndrome\|Autoimmune Diseases | Recruiting | Early Phase 1 | To evaluate the safety, tolerability, pharmacokinetics, pharmacodynamics, and efficacy of CD19 or CD19-BCMA CAR-T in patients with active autoimmune diseases | NO |
| Functional reprogramming | NCT06544330 | SYNCAR-001 + STK-009 | Systemic Lupus Erythematosus\|Lupus Nephritis\|Systemic Sclerosis | Active, not recruiting | Phase 1 | To evaluate the safety and tolerability of SYNCAR-001 + STK-009 in subjects with severe, refractory systemic autoimmune rheumatic disease | NO |
| Functional reprogramming | NCT06530849 | GC012F Injection | Refractory Systemic Lupus Erythematosus | Active, not recruiting | Phase 1/2 | To assess the safety and efficacy of GC012F Injection in subjects with refractory systemic lupus erythematosus | NO |
| Functional reprogramming | NCT06519565 | PRG-1801 | Immune Thrombocytopenia | Recruiting | Early Phase 1 | To evaluate the safety, tolerability, and preliminary efficacy of PRG-1801 in patients with relapsed/refractory Immune Thrombocytopenia. | NO |
| Functional reprogramming | NCT06513429 | IM19 CAR-T cells | Refractory Systemic Lupus Erythematosus | Recruiting | NA | To evaluate the efficacy and safety of IM19 CAR-T cells in patients with refractory Systemic Lupus Erythematosus. | NO |
| Functional reprogramming | NCT06503224 | SCAR02 | Autoimmune Diseases | Recruiting | NA | To evaluate the safety and efficacy of anti-BCMA and CD19 CAR-T cell therapy in patients with refractory autoimmune diseases | NO |
| Functional reprogramming | NCT06503237 | SWK002 | Rheumatoid Arthritis | Recruiting | NA | To evaluate the safety and efficacy of SWK002 in patients with D2T-Rheumatoid Arthritis | NO |
| Functional reprogramming | NCT06497361 | PRG-2311 | Lupus Nephritis\|IgG4-related Disease | Recruiting | Early Phase 1 | To evaluate the safety and effectiveness of PRG-2311 in refractory lupus nephritis and IgG4-related disease | NO |
| Functional reprogramming | NCT06497387 | PRG-1801 | Lupus Nephritis\|IgG4-related Disease | Recruiting | Early Phase 1 | To evaluate the safety and effectiveness of PRG-1801 BCMA-targeting CAR-T cells in the treatment of refractory Lupus Nephritis and IgG4-related Disease. | NO |
| Functional reprogramming | NCT06475495 | KYV101; Rituximab | Rheumatoid Arthritis | Active, not recruiting | Phase 1/2 | To compare B-cell depletion by Rituximab and anti-CD19 CAR-T therapy, and to assess safety, clinical efficacy, immunological impact, antibody positivity, and long-term remission/drug-free survival in patients with active, ACPA-positive, treatment-refractory Rheumatoid Arthritis. | NO |
| Functional reprogramming | NCT06464679 | anti-CD19 CAR NK cells | Autoimmune Diseases | Recruiting | Phase 1 | To evaluate the safety, feasibility, and efficacy of CD19 CAR-NK cells in patients with relapsed/refractory autoimmune diseases | NO |
| Functional reprogramming | NCT06465147 | SCRI-CAR19v3 | Systemic Lupus Erythematosus | Recruiting | Phase 1 | To examine the safety, feasibility, and efficacy of SCRI-CAR19v3 in pediatric and young adult participants with treatment-refractory Systemic Lupus Erythematosus | NO |
| Functional reprogramming | NCT06451159 | KYV-101 | Progressive Multiple Sclerosis | Active, not recruiting | Phase 1 | To evaluate the safety, tolerability, CNS penetration, target engagement, and preliminary efficacy of KYV-101 in participants with treatment-refractory progressive multiple sclerosis | NO |
| Functional reprogramming | NCT06434363 | AD-PluReceptor-NK cells; Tafasitamab-cxix | Autoimmune Disorders\|Systemic Sclerosis\|Systemic Lupus Erythematosus\|Lupus\|Lupus Nephritis | Recruiting | Phase 1/2 | To determine the safety, tolerability, optimal cell dose, and efficacy of AD-PluReceptor-NK cells plus tafasitamab-cxix and lymphodepleting chemotherapy in patients with systemic sclerosis and systemic lupus erythematosus, including lupus nephritis. | NO |
| Functional reprogramming | NCT06435897 | fetal MSCs combined with 4SCAR T cells | Autoimmune Diseases | Recruiting | Phase 1/2 | To assess the feasibility, safety, efficacy, and long-term effects of fetal MSCs combined with 4SCAR T cells in patients with autoimmune diseases. | NO |
| Functional reprogramming | NCT06428188 | BCMA/CD19 CAR-T cells | Autoimmune Diseases\|Systemic Lupus Erythematosus\|Systemic Lupus Erythematosus Acute\|Sjogren's Syndrome | Recruiting | Phase 1/2 | To evaluate the efficacy, safety, feasibility, function, and persistence of BCMA/CD19 CAR-T cells in patients with relapsed/refractory autoimmune diseases. | NO |
| Functional reprogramming | NCT06400303 | KYV-101 | Systemic Sclerosis | Active, not recruiting | Phase 1/2 | To investigate KYV-101, an autologous fully human anti-CD19 CAR T-cell therapy, in adult subjects with systemic sclerosis | NO |
| Functional reprogramming | NCT06384976 | KYV-101; Anti-CD20 mAB | Primary Progressive Multiple Sclerosis\|Secondary Progressive Multiple Sclerosis | Active, not recruiting | Phase 2 | To evaluate KYV-101, an autologous fully human anti-CD19 CAR-T therapy, in subjects with refractory primary and secondary progressive Multiple Sclerosis. | NO |
| Functional reprogramming | NCT06379646 | YTS109 | Systemic Lupus Erythematosus\|Systemic Sclerosis\|Inflammatory Myopathy\|Anti-Neutrophil Cytoplasmic Antibody-Associated Vasculitis\|Antiphospholipid Syndrome\|Sjogren's Syndrome | Recruiting | NA | To evaluate the safety, initial efficacy, pharmacokinetics, and pharmacodynamics of YTS109 cell injection in subjects with recurrent/refractory autoimmune disease | NO |
| Functional reprogramming | NCT06371040 | CD19-BCMA Targeted CAR-T | Myasthenia Gravis | Recruiting | Phase 1 | To evaluate the safety and preliminary effectiveness of CD19-BCMA Targeted CAR-T therapy in refractory generalized Myasthenia Gravis and explore the maximum tolerated dose | NO |
| Functional reprogramming | NCT06359041 | CABA-201 | Generalized Myasthenia Gravis (gMG) | Active, not recruiting | Phase 1/2 | To evaluate the safety and efficacy of autologous CD19-specific chimeric antigen receptor T cells in participants with generalized myasthenia gravis | NO |
| Functional reprogramming | NCT06352281 | CAR-T cells | ITP - Immune Thrombocytopenia | Recruiting | Phase 1/2 | To evaluate the efficacy and safety of CAR-T cells therapy in patients with chronic or refractory primary immune thrombocytopenia. | NO |
| Functional reprogramming | NCT06349343 | CD19/BCMA CAR-T cell therapy | Systemic Lupus Erythematosus | Recruiting | Phase 1 | To investigate the safety, tolerability, preliminary efficacy, pharmacokinetics, pharmacodynamics, and feasibility of CD19/BCMA CAR-T cell therapy in patients with refractory/moderate-to-severe Systemic Lupus Erythematosus. | NO |
| Functional reprogramming | NCT06350110 | BH002 injection | Systemic Lupus Erythematosus\|Lupus Nephritis\|Autoimmune Diseases\|Anti-Neutrophil Cytoplasmic Antibody-Associated Vasculitis\|Granulomatous Polyangiitis\|Microscopic Polyangiitis\|Systemic Sclerosis\|Idiopathic Inflammatory Myopathies\|Sjogren's Syndrome | Recruiting | Phase 1/2 | To identify the highest tolerated dose of BH002 injection in patients with refractory or relapsed systemic lupus erythematosus | NO |
| Functional reprogramming | NCT06347718 | anti-CD19 CAR T cell therapy | Systemic Lupus Erythematosus\|Systemic Sclerosis\|Dermatomyositis\|Polymyositis | Recruiting | Phase 1/2 | To evaluate the safety and efficacy of anti-CD19 CAR T-cell therapy in systemic B cell-mediated autoimmune diseases. | NO |
| Functional reprogramming | NCT06342960 | KYV-101 | Lupus Nephritis\|Lupus Nephritis - WHO Class III\|Lupus Nephritis - WHO Class IV | Active, not recruiting | Phase 1/2 | To evaluate the safety and efficacy of KYV-101 anti-CD19 CAR-T cell therapy in subjects with refractory lupus nephritis | NO |
| Functional reprogramming | NCT06340750 | LMY-920 | Systemic Lupus Erythematosus | Recruiting | Phase 1 | To examine the safety and recommended phase 2 dose of LMY-920 in adult patients with refractory Systemic Lupus Erythematosus. | NO |
| Functional reprogramming | NCT06333483 | Obecabtagene autoleucel | Systemic Lupus Erythematosus | Recruiting | Phase 1 | To determine the safety, tolerability, preliminary efficacy, and pharmacokinetics of Obecabtagene autoleucel in patients with severe, refractory Systemic Lupus Erythematosus. | NO |
| Functional reprogramming | NCT06328777 | CABA-201 | Systemic Sclerosis\|Scleroderma | Recruiting | Phase 1/2 | To evaluate the safety and efficacy of CABA-201, a CD19-CAR T cell therapy, in subjects with systemic sclerosis. | NO |
| Functional reprogramming | NCT06318533 | KN5501 | Autoimmune Diseases | Recruiting | Early Phase 1 | To evaluate the safety, feasibility, and effectiveness of KN5501 in patients with relapsed/refractory B-cell related autoimmune diseases. | NO |
| Functional reprogramming | NCT06316076 | CD19-CAR-DNT cells | Systemic Lupus Erythematosus\|Anti-Neutrophil Cytoplasmic Antibody-Associated Vasculitis\|Idiopathic Inflammatory Myopathies\|Systemic Sclerosis | Recruiting | Phase 1 | To evaluate the maximum tolerated dose, safety, preliminary efficacy, and pharmacokinetic profile of CD19-CAR-DNT cells in subjects with relapsed/refractory autoimmune diseases. | NO |
| Functional reprogramming | NCT06316791 | CNCT19 | Lupus Erythematosus, Systemic | Recruiting | Early Phase 1 | To evaluate the safety and tolerability of CNCT19 in patients with refractory autoimmune diseases. | NO |
| Functional reprogramming | NCT06308978 | FT819 | Antineutrophilic Cytoplasmic Antibody (ANCA)- Associated Vasculitis (AAV)\|Idiopathic Inflammatory Myositis (IIM)\|Systemic Sclerosis (SSc)\|Systemic Lupus Erythematosus (SLE)\|Lupus Nephritis | Recruiting | Phase 1 | To evaluate the safety, pharmacokinetics, and anti-B-cell activity of FT819, with or without auxiliary medicinal product, in participants with B-cell mediated autoimmune diseases. | NO |
| Functional reprogramming | NCT06294236 | SC291 | Systemic Lupus Erythematosus\|ANCA-associated Vasculitis\|Granulomatosis with Polyangiitis\|Microscopic Polyangiitis | Active, not recruiting | Phase 1 | To evaluate the safety, tolerability, preliminary clinical response, cellular kinetics, and exploratory assessments of SC291 in subjects with severe relapsed or refractory autoimmune diseases. | NO |
| Functional reprogramming | NCT06285279 | FKC289 | Lupus Nephritis\|ANCA-associated Vasculitis\|Membranous Nephropathy - PLA2R Induced\|IgG4-Related Diseases | Recruiting | Phase 1 | To evaluate the safety and efficacy of BCMA/CD19 dual targeted CAR-T cells in participants with autoimmune kidney diseases. | NO |
| Functional reprogramming | NCT06279923 | CD19-BAFF Targeted CAR T-cells | Autoimmune Diseases | Recruiting | Early Phase 1 | To evaluate the safety and efficacy of CD19-BAFF CAR-T cell therapy for autoimmune diseases. | NO |
| Functional reprogramming | NCT06277427 | PRG-1801 | Lupus Nephritis\|ANCA Associated Vasculitis | Recruiting | NA | To evaluate the safety and efficacy of PRG-1801 BCMA-targeting CAR-T cells in patients with refractory Lupus Nephritis and ANCA-associated Vasculitis. | NO |
| Functional reprogramming | NCT06255028 | CNTY-101 | Systemic Lupus Erythematosus\|Lupus Nephritis\|Idiopathic Inflammatory Myopathies\|Diffuse Cutaneous Systemic Sclerosis | Active, not recruiting | Phase 1 | To evaluate the safety and efficacy of CNTY-101 in participants with refractory B cell-mediated autoimmune diseases. | NO |
| Functional reprogramming | NCT06249438 | C-CAR168 | Systemic Lupus Erythematosus (SLE)\|Immune-mediated Necrotizing Myopathy (IMNM)\|Neuromyelitis Optica Spectrum Disorders (NMOSD)\|Multiple Sclerosis (MS)\|Myasthenia Gravis\|Systemic Sclerosis (SSc) | Recruiting | Phase 1 | To evaluate the safety and efficacy of C-CAR168, an autologous bi-specific CD20/BCMA-directed CAR-T therapy, in adult patients with autoimmune diseases refractory to standard therapy. | NO |
| Functional reprogramming | NCT06231368 | CNCT19 CAR-T cell therapy | Autoimmune Hemolytic Anemia | Active, not recruiting | Phase 1 | To evaluate the safety, tolerability, and efficacy of CNCT19 CAR-T cell therapy in patients with autoimmune hemolytic anemia after failure of three or more lines of therapy. | NO |
| Functional reprogramming | NCT06222853 | anti-CD19-CAR-T cells | Systemic Lupus Erythematosus | Active, not recruiting | Phase 1 | To assess the safety and efficacy of anti-CD19 CAR-T cells in the treatment of refractory Systemic Lupus Erythematosus. | NO |
| Functional reprogramming | NCT06220201 | CC-97540 | Multiple Sclerosis\|Myasthenia Gravis | Recruiting | Phase 1 | To evaluate the safety, tolerability, efficacy, and drug levels of CC-97540 in participants with relapsing forms of Multiple Sclerosis, progressive forms of Multiple Sclerosis, or refractory Myasthenia Gravis. | NO |
| Functional reprogramming | NCT06193889 | KYV-101 | Myasthenia Gravis\|Generalized Myasthenia Gravis | Recruiting | Phase 2/3 | To evaluate KYV-101 versus ongoing standard-of-care immunosuppressive therapy in patients with generalized myasthenia gravis. | NO |
| Functional reprogramming | NCT06189157 | MB-CART19.1 | SLE - Systemic Lupus Erythematosus | Recruiting | Phase 1/2 | To evaluate the safety and efficacy of MB-CART19.1 in patients with refractory systemic lupus erythematosus. | NO |
| Functional reprogramming | NCT06154252 | CABA-201 | Idiopathic Inflammatory Myopathy\|Dermatomyositis\|Anti-Synthetase Syndrome\|Immune-Mediated Necrotizing Myopathy\|Juvenile Dermatomyositis\|Juvenile Polymyositis\|Juvenile Idiopathic Inflammatory Myopathy (JIIM)\|Juvenile Myositis | Recruiting | Phase 2/3 | To evaluate the safety and efficacy of autologous CD19-specific chimeric antigen receptor T cells in subjects with active idiopathic inflammatory myopathy or juvenile idiopathic inflammatory myopathy. | NO |
| Functional reprogramming | NCT06152172 | KYV-101 | Idiopathic Inflammatory Myopathies\|Diffuse Cutaneous Systemic Sclerosis\|SLE Nephritis\|ANCA Associated Vasculitis | Active, not recruiting | Phase 1 | To assess the safety, tolerability, and clinical activity of KYV-101 in adult subjects with B cell-driven autoimmune diseases. | NO |
| Functional reprogramming | NCT06138132 | KYV-101 | Multiple Sclerosis\|Multiple Sclerosis, Primary Progressive\|Multiple Sclerosis, Secondary Progressive | Recruiting | Phase 1 | To evaluate KYV-101, an autologous fully human anti-CD19 CAR-T therapy, in subjects with non-relapsing and progressive forms of Multiple Sclerosis. | NO |
| Functional reprogramming | NCT06106893 | CD19 Universal CAR-γδ T Cells | Systemic Lupus Erythematosus | Recruiting | Phase 1/2 | To investigate the safety, tolerability, preliminary efficacy, pharmacokinetics, and pharmacodynamics of CD19 Universal CAR-γδ T cells in active systemic lupus erythematosus. | NO |
| Functional reprogramming | NCT06106906 | CD19 CAR-T cell infusion | Systemic Lupus Erythematosus | Recruiting | Phase 1/2 | To investigate the safety, tolerability, preliminary efficacy, pharmacokinetics, and pharmacodynamics of CD19 CAR-T cell therapy in active systemic lupus erythematosus. | NO |
| Functional reprogramming | NCT06056921 | CD19 targeted CAR-T cells | SLE (Systemic Lupus)\|Sjogren's Syndrome\|Systemic Scleroderma\|Dermatomyositis\|Anti-Neutrophil Cytoplasmic Antibody-Associated Vasculitis | Recruiting | Phase 1 | To evaluate the efficacy and safety of CD19 targeted CAR-T cells in patients with refractory autoimmune diseases. | NO |
| Functional reprogramming | NCT06058078 | RY_SW01 cell injection | Lupus Nephritis | Recruiting | Phase 2 | To evaluate the safety, tolerability, and efficacy of RY_SW01 cell injection in patients with active lupus nephritis. | NO |
| Functional reprogramming | NCT06038474 | Descartes-08 | Systemic Lupus Erythematosus (SLE) | Active, not recruiting | Phase 2 | To evaluate the safety, tolerability, and manufacturing feasibility of Descartes-08 CAR T cells in patients with systemic lupus erythematosus. | NO |
| Functional reprogramming | NCT06010472 | KN5501 | Systemic Lupus Erythematosus (SLE) | Recruiting | Early Phase 1 | To evaluate the safety and effectiveness of allogeneic anti-CD19 CAR-NK cells in patients with moderate to severe refractory systemic lupus erythematosus | NO |
| Functional reprogramming | NCT05988216 | BRL-301 | Systemic Lupus Erythematosus (SLE) | Recruiting | NA | To assess the efficacy and safety of BRL-301 in refractory systemic lupus erythematosus | NO |
| Functional reprogramming | NCT05938725 | KYV-101 | Lupus Nephritis\|Lupus Nephritis - World Health Organization (WHO) Class III\|Lupus Nephritis - WHO Class IV | Active, not recruiting | Phase 1/2 | To evaluate the safety and efficacy of KYV-101 anti-CD19 CAR-T cell therapy in subjects with refractory lupus nephritis | NO |
| Functional reprogramming | NCT05869955 | CC-97540 | Systemic Lupus Erythematosus\|Idiopathic Inflammatory Myopathy\|Systemic Sclerosis\|Rheumatoid Arthritis | Recruiting | Phase 1 | To establish the tolerability, preliminary efficacy, and pharmacokinetics of CC-97540 in participants with severe, refractory autoimmune diseases. | NO |
| Functional reprogramming | NCT06121297 | CABA-201 | Systemic Lupus Erythematosus\|Lupus Nephritis | Recruiting | Phase 1/2 | To evaluate the safety and efficacy of CABA-201 in subjects with active systemic lupus erythematosus | NO |
| Functional reprogramming | NCT06106672 | CNP-106 | Myasthenia Gravis\|Generalized Myasthenia\|AChR Myasthenia Gravis\|MuSK MG | Active, not recruiting | Phase 1/2 | To evaluate the safety, tolerability, pharmacodynamics, and efficacy of CNP-106 in subjects with generalized Myasthenia Gravis | NO |
| Functional reprogramming | NCT05798117 | YTB323 | Systemic Lupus Erythematosus\|Lupus Nephritis | Active, not recruiting | Phase 1/2 | To assess the safety, efficacy, and cellular kinetics of YTB323 in participants with severe refractory systemic lupus erythematosus | NO |
| Functional reprogramming | NCT05757713 | teplizumab | Diabetes Mellitus, Type 1 | Active, not recruiting | Phase 4 | To assess the safety and pharmacokinetics of teplizumab in pediatric participants with Stage 2 Type 1 Diabetes who are <8 years of age. | NO |
| Functional reprogramming | NCT06292923 | Foralumab | Secondary Progressive Multiple Sclerosis | Recruiting | Phase 2 | To evaluate the safety, tolerability, and PET-based effects of intranasal foralumab compared with placebo after 12 weeks of treatment. | NO |
| Functional reprogramming | NCT06890923 | Foralumab | Non-Active Secondary Progressive Multiple Sclerosis | Recruiting | Phase 2 | To evaluate the 6-month safety and clinical effect of open-label nasal foralumab in patients with non-active SPMS. | NO |
| Functional reprogramming | NCT05683990 | Diamyd | Diabetes Mellitus, Type 1\|Autoimmune Diseases\|Prevention | Active, not recruiting | Phase 2 | To evaluate the safety and feasibility of intralymphatic administration of Diamyd in individuals at risk for type 1 diabetes carrying the HLA DR3-DQ2 haplotype | NO |
| Functional reprogramming | NCT05459870 | 4SCAR T cells | Autoimmune Diseases | Recruiting | Phase 1/2 | To assess the feasibility, safety, efficacy, function, and persistence of CAR-T cell therapy in patients with autoimmune disease | NO |
| Functional reprogramming | NCT05241444 | CD4^LVFOXP3 | IPEX | Recruiting | Phase 1 | To evaluate the manufacturing feasibility, safety, in vivo persistence, and early disease impact of CD4^LVFOXP3 in participants with IPEX syndrome. | NO |
| Functional reprogramming | NCT05018585 | Diamyd | Type 1 Diabetes Mellitus | Active, not recruiting | Phase 3 | To evaluate the efficacy and safety of Diamyd in preserving endogenous beta cell function in adolescents and adults with recently diagnosed Type 1 Diabetes carrying the HLA DR3-DQ2 haplotype. | NO |
| Functional reprogramming | NCT04590872 | PIpepTolDC | Type 1 Diabetes Mellitus | Active, not recruiting | Phase 1 | To evaluate the safety and feasibility of autologous tolerogenic dendritic cells loaded with proinsulin peptide C19-A3 in patients with Type 1 Diabetes. | NO |
| Functional reprogramming | NCT06801119 | HN2301 | SLE Scleroderma Rheumatoid Arthritis | Recruiting | Phase 1 | Evaluate the safety and preliminary efficacy of HN2301 in autoimmune diseases. | NO |
| Functional reprogramming | NCT06566261 | ABA-101 | Progressive Multiple Sclerosis | Active, not recruiting | Phase 1 | To evaluate the safety and effects of a single dose of ABA-101 in participants with progressive multiple sclerosis. | NO |
| Functional reprogramming | NCT07205315 | GT801 Injection | B-cell Acute Lymphoblastic Leukemia (B-ALL) Chronic Lymphocytic Leukemia (CLL)  B-cell Non-Hodgkin's Lymphoma (B-NHL) Autoimmune Hemolytic Anemia (AIHA) | Recruiting | Early Phase 1 | To evaluate the safety and efficacy of GT801 injection in adult patients with relapsed/refractory CD19-positive B-cell hematologic malignancies and autoimmune hemolytic anemia. | NO |
| Functional reprogramming | NCT06201416 | SBT777101 | RA | Recruiting | Phase 1 | To evaluate the safety, tolerability, pharmacokinetics, pharmacodynamics, and activity of single ascending doses of SBT777101 in subjects with rheumatoid arthritis. | NO |
| Functional reprogramming | NCT07477639 | TRX319 | Primary Progressive Multiple Sclerosis  Secondary Progressive Multiple Sclerosis | Recruiting | Phase 1/2 | To assess the safety, tolerability, optimal dose, and preliminary efficacy of TRX319 in PPMS/SPMS, including the need for bendamustine preconditioning. | NO |
| Functional reprogramming | NCT07526350 | MTS109 | Systemic Lupus Erythematosus Idiopathic Inflammatory Myopathies Systemic Sclerosis (SSc) ANCA-Associated Vasculitis (AAV) Sjogren's Syndrome (SS) | Recruiting | Early Phase 1 | To evaluate the safety, tolerability, and preliminary efficacy of MTS109 in patients with moderate to severe refractory autoimmune diseases. | NO |
| Functional reprogramming | NCT07413835 | HN2302 Injection | Refractory Myasthenia Gravis | Recruiting | NA | To evaluate the safety, tolerability, and preliminary efficacy of HN2302 in patients with refractory myasthenia gravis. | NO |
| Functional reprogramming | NCT06958939 | S103 | Myasthenia Gravis, Generalized | Active, not recruiting | Phase 1 | To evaluate the safety and preliminary efficacy of S103 CAR-T cells in patients with refractory generalized myasthenia gravis. | NO |
| Functional reprogramming | NCT06917742 | CPTX2309 | RA SLE | Recruiting | Phase 1 | To assess the safety, tolerability, PK, and PD of intravenous CPTX2309 in healthy subjects and patients with moderate-to-severe RA or SLE. | NO |
| Functional reprogramming | NCT07109986 | UB-VV410 | SLE Lupus Nephritis | Recruiting | Phase 1 | To assess the safety, efficacy, and PK/PD of UB-VV410 in adults with active refractory SLE or lupus nephritis | NO |
| Functional reprogramming | NCT07049081 | LUCAR-G79 T cells | Relapsed/Refractory Autoimmune Diseases | Recruiting | Phase 1 | To evaluate the safety, tolerability, pharmacokinetics, pharmacodynamics, and efficacy of LUCAR-G79 T cells in relapsed or refractory autoimmune diseases. | NO |
| Immunometabolism | NCT07400757 | Atorvastatin; Vitamin D | Thyroid Eye Disease, TED\|Graves Ophthalmopathy | Recruiting | NA | To assess whether statin treatment and vitamin D supplementation modify disease activity in active thyroid eye disease and to evaluate their safety. | NO |
| Immunometabolism | NCT07151157 | Sirolimus | Adult-Onset Still's Disease | Active, not recruiting | NA | To evaluate the therapeutic value and anti-inflammatory effects of sirolimus in refractory adult-onset Still’s disease, including its effects on ZFP36L2 expression, inflammatory cytokine mRNA degradation, inflammation, and disease activity. | NO |
| Immunometabolism | NCT07065968 | Teriflunomide; Dexamethasone | Immune Thrombocytopenia (ITP) | Recruiting | Phase 2 | To evaluate the efficacy and safety of teriflunomide plus high-dose dexamethasone versus high-dose dexamethasone alone as first-line therapy for newly diagnosed adult primary immune thrombocytopenia. | NO |
| Immunometabolism | NCT06776510 | nicotinamide adenine dinucleotide; nicotinamide mononucleotide | Immune Thrombocytopenia\|Treatment | Recruiting | Phase 1/2 | To evaluate the safety and efficacy of nicotinamide adenine dinucleotide and nicotinamide mononucleotide in primary immune thrombocytopenia. | NO |
| Immunometabolism | NCT06663618 | prednison; Mycophenolate mofetil | IgG4-related Disease | Recruiting | NA | To compare short-term glucocorticoid monotherapy with short-term glucocorticoids plus mycophenolate mofetil in IgG4-related disease. | NO |
| Immunometabolism | NCT06649136 | Methotrexate | Rheumatoid Arthritis | Recruiting | Phase 4 | To compare the efficacy and safety of 25 mg oral methotrexate versus 25 mg subcutaneous methotrexate in active rheumatoid arthritis. | NO |
| Immunometabolism | NCT06640309 | Nicotinamide Tablet; conventional synthetic antirheumatic drugs | Rheumatoid Arthritis | Recruiting | Phase 2 | To evaluate the efficacy and safety of nicotinamide supplementation in patients with rheumatoid arthritis receiving conventional synthetic disease-modifying antirheumatic drugs. | NO |
| Immunometabolism | NCT06468709 | Prednisone; Mycophenolate mofetil | Type 1 AIP | Recruiting | Phase 4 | To evaluate the effects of prednisone monotherapy versus prednisone plus mycophenolate mofetil maintenance therapy on relapse-free survival and disease remission in high-risk type 1 autoimmune pancreatitis. | NO |
| Immunometabolism | NCT06463587 | Cladribine | Generalized Myasthenia Gravis | Recruiting | Phase 3 | To evaluate the efficacy and safety of a novel oral cladribine formulation versus placebo in generalized myasthenia gravis. | NO |
| Immunometabolism | NCT06463743 | metformin | Multiple Sclerosis | Recruiting | Phase 2 | To evaluate the safety, tolerability, and potential neuroprotective effects of metformin as monotherapy or add-on therapy in older adults with multiple sclerosis. | NO |
| Immunometabolism | NCT06068179 | Mycophenolate Mofetil; Methimazole | Graves' Disease | Recruiting | Phase 2/3 | To evaluate the efficacy and safety of mycophenolate mofetil plus methimazole in newly diagnosed Graves’ disease. | NO |
| Immunometabolism | NCT05961644 | Cladribine | Multiple Sclerosis, Secondary Progressive\|Multiple Sclerosis | Recruiting | Phase 2/3 | To evaluate the efficacy and safety of subcutaneous cladribine versus placebo in nonrelapsing secondary progressive multiple sclerosis. | NO |
| Immunometabolism | NCT05946564 | Pioglitazone | ANCA Associated Vasculitis\|Rapidly Progressive Glomerulonephritis\|Crescentic Glomerulonephritis | Recruiting | Phase 3 | To evaluate the efficacy and safety of pioglitazone for improving renal outcomes in ANCA-associated vasculitis. | NO |
| Immunometabolism | NCT05925023 | Sirolimus | Warm Autoimmune Hemolytic Anemia | Recruiting | NA | To explore the efficacy and safety of sirolimus in refractory or relapsed warm antibody autoimmune hemolytic anemia. | NO |
| Immunometabolism | NCT05916781 | Mycophenolate Mofetil; Tacrolimus; Glucocorticoid | Systemic Lupus Erythematosus\|Lupus Nephritis | Recruiting | Phase 4 | To evaluate the efficacy, safety, and tolerability of mycophenolate mofetil plus tacrolimus for maintaining remission during glucocorticoid tapering in lupus nephritis. | NO |
| Immunometabolism | NCT05893225 | Metformin Hydrochloride | Multiple Sclerosis\|Primary Progressive Multiple Sclerosis\|Secondary-progressive Multiple Sclerosis | Active, not recruiting | Phase 2 | To evaluate the effects of adjunctive metformin hydrochloride on brain remyelination and neurodegeneration in multiple sclerosis. | NO |
| Immunometabolism | NCT05789030 | Prednisone；Iguratimod; Leflunomide | Autoimmune Diseases | Recruiting | NA | To compare the efficacy and safety of prednisone plus iguratimod versus prednisone plus leflunomide in IgG4-RD patients with superficial organ involvement. | NO |
| Immunometabolism | NCT05538208 | Mycophenolate Mofetil | Lupus Nephritis | Recruiting | Phase 2 | To evaluate the efficacy and safety of pharmacokinetics-guided precision dosing versus body surface area–based dosing of mycophenolate mofetil in pediatric proliferative lupus nephritis. | NO |
| Immunometabolism | NCT05353829 | Methotrexate | Rheumatoid Arthritis | Active, not recruiting | Phase 4 | To compare the efficacy, tolerability, and patient preference of oral versus subcutaneous methotrexate in early rheumatoid arthritis. | NO |
| Immunometabolism | NCT05349006 | Azathioprine | Central Nervous System Inflammation\|MOG-IgG Associated Disease | Recruiting | Phase 3 | To evaluate the efficacy of azathioprine in preventing relapse after a first attack of MOG-IgG–associated disease. | NO |
| Immunometabolism | NCT05201638 | IMU-838 | Multiple Sclerosis, Relapsing-Remitting | Active, not recruiting | Phase 3 | To evaluate the efficacy, safety, and tolerability of IMU-838 versus placebo in adults with relapsing multiple sclerosis. | NO |
| Immunometabolism | NCT05134441 | IMU-838 | Multiple Sclerosis | Active, not recruiting | Phase 3 | To evaluate the efficacy, safety, and tolerability of IMU-838 in adults with relapsing multiple sclerosis. | NO |
| Immunometabolism | NCT00775476 | N-acetylcysteine | Systemic Lupus Erythematosus | Active, not recruiting | Phase 2 | To evaluate the efficacy, tolerability, and safety of N-acetylcysteine in systemic lupus erythematosus. | NO |
| Immunometabolism | NCT05180591 | Bacillus Calmette-Guérin | Diabetes Mellitus, Type 1\|Diabetes type1\|Autoimmune Diabetes | Recruiting | Phase 2 | To investigate whether repeat BCG vaccinations can confer beneficial immune and metabolic effects in pediatric type 1 diabetes | NO |
| Immunometabolism | NCT07282574 | RO7268489 | Progressive Multiple Sclerosis | Recruiting | Phase 2 | To evaluate the safety, PK/PD, and efficacy of RO7268489 as add-on therapy to ocrelizumab in progressive MS. | NO |
| Immunometabolism | NCT05866536 | Bacillus Calmette-Guérin | Diabetes Mellitus\|Type 1 Diabetes\|Diabetes type1\|Autoimmune Diabetes | Recruiting | Phase 2 | To investigate whether repeat Bacillus Calmette-Guérin vaccinations can confer beneficial immune and metabolic effects in new-onset pediatric Type 1 Diabetes. | NO |

The screening process was performed as follows. The search was conducted in ClinicalTrials.gov on June 11, 2026, using “Autoimmune Diseases” as the search term. Study status was restricted to “Recruiting” and “Active, not recruiting,” and the search period was limited to June 11, 2021, to June 11, 2026. This initial search yielded 1,953 potentially relevant clinical trials.

The retrieved records were then manually screened according to disease type, intervention, study objective, mechanistic description, and primary and secondary outcome measures. Trials that were not directly related to immune-regulatory mechanisms in autoimmune diseases were excluded. The remaining trials were further categorized according to their putative therapeutic mechanisms. Ultimately, 245 ongoing clinical trials were included, comprising 26 trials related to immunometabolism-targeted therapies and 219 trials related to functional reprogramming-based therapies. No eligible ongoing trials targeting post-transcriptional regulation or epigenetic regulation were identified according to our screening criteria.
